# Regulatory network established by transcription factors transmits drought stress signals in plant

**DOI:** 10.1007/s44154-022-00048-z

**Published:** 2022-07-14

**Authors:** Yongfeng Hu, Xiaoliang Chen, Xiangling Shen

**Affiliations:** Key Laboratory of Three Gorges Regional Plant Genetics and Germplasm Enhancement, Biotechnology Research Center, China Three Gorges University, Yichang, 443002 Hubei China

**Keywords:** Plant, Drought tolerance, Transcription factor, Regulatory network, Direct target

## Abstract

Plants are sessile organisms that evolve with a flexible signal transduction system in order to rapidly respond to environmental changes. Drought, a common abiotic stress, affects multiple plant developmental processes especially growth. In response to drought stress, an intricate hierarchical regulatory network is established in plant to survive from the extreme environment. The transcriptional regulation carried out by transcription factors (TFs) is the most important step for the establishment of the network. In this review, we summarized almost all the TFs that have been reported to participate in drought tolerance (DT) in plant. Totally 466 TFs from 86 plant species that mostly belong to 11 families are collected here. This demonstrates that TFs in these 11 families are the main transcriptional regulators of plant DT. The regulatory network is built by direct protein-protein interaction or mutual regulation of TFs. TFs receive upstream signals possibly via post-transcriptional regulation and output signals to downstream targets via direct binding to their promoters to regulate gene expression.

**Supplementary Information:**

The online version contains supplementary material available at 10.1007/s44154-022-00048-z.

## Introduction

Sessile plants constantly deal with adverse environmental changes in their entire lifetime through a dynamic responsive system. Perceiving various stresses by specific sensors immediately triggers intracellular signals, which are transmitted into the nucleus to induce transcriptional reprogramming for cellular and physiological reactions. Drought is one of the most detrimental abiotic stresses for plant growth. A few review papers focusing on general mechanism of drought resistance, drought-responding long-distance signaling, ABA-dependent and -independent phosphorylation networks in cellular signal transduction have been published (Fang and Xiong 2015; Gong et al. 2020b; Takahashi et al. 2018; Yoshida et al. 2014). Transcriptional regulators including transcription factors (TFs), Mediators and chromatin regulators that are involved in drought tolerance (DT) have also been summarized (Chang et al. 2020; Chong et al. 2020; Han and Wagner 2014; Kim et al. 2010; Takahashi et al. 2018). However, only a small proportion of reported TFs that are related to DT were mentioned in the reviews. Decades of research has revealed hundreds of DT-related TFs in different plant species, which will be discussed in this paper.

TFs, binding specific DNA elements, regulate gene expression by directly affecting binding affinity of RNA polymerase II (Pol II) to core promoters or recruiting chromatin regulators to change local chromatin accessibility (Spitz and Furlong 2012). There are at least 56 families of TFs in plants (Jin et al. 2017b), many of which are plant-specific, implying that the distinctive regulatory networks have been established in plants to transmit cellular signal for development and stress response. Some of plant TF families are large families containing more than one hundred members such as, MYB, bZIP, ERF, WRKY, bZIP, ZnF and NAC (Jin et al. 2017b). However, it is not clear whether all of plant TF families are involved in stress response. In other words, is there a possibility that only some specific TF families or subfamilies are responsible? To address this question, we collected nearly all the reported TFs (466 TFs from 86 plant species), whose roles in DT have been functionally studied (Table S1) by forward or reverse genetic methods. We found that these TFs mainly fell into 11 families including NAC, ERF, WRKY, bZIP, MYB, HD-ZIP, ZnF, bHLH, ASR, NF-Y and HSF. They act as either positive or negative regulators of DT. Overexpression of positive regulators enhances drought resistance, and mutation or silencing of these genes decreases drought resistance. By contrast, negative regulators influence DT in an opposite way. It is worth noting that a number of genes from some species were functionally analyzed by ectopic expression in the model organisms like Arabidopsis, rice, tobacco, etc. This may not truly reflect the intrinsic DT mechanism in these species. The post-transcriptional regulations of TFs, which could possibly serve as the approaches to receive upstream signals, and the direct downstream target genes of TFs are also reviewed in this paper.

### DT-related TFs

Most TF families are divided into several classes or groups based on phylogenetic analysis. In order to see if specific classes of each family TFs would be responsible for drought response, we sorted out the DT-related TFs and found that more TFs in some classes have been reported to be involved in DT than the others, which is exemplified by ATAF subgroup of NAC family and group A of bZIP family (Table S1).

#### NAC family

**Fig. 1:**
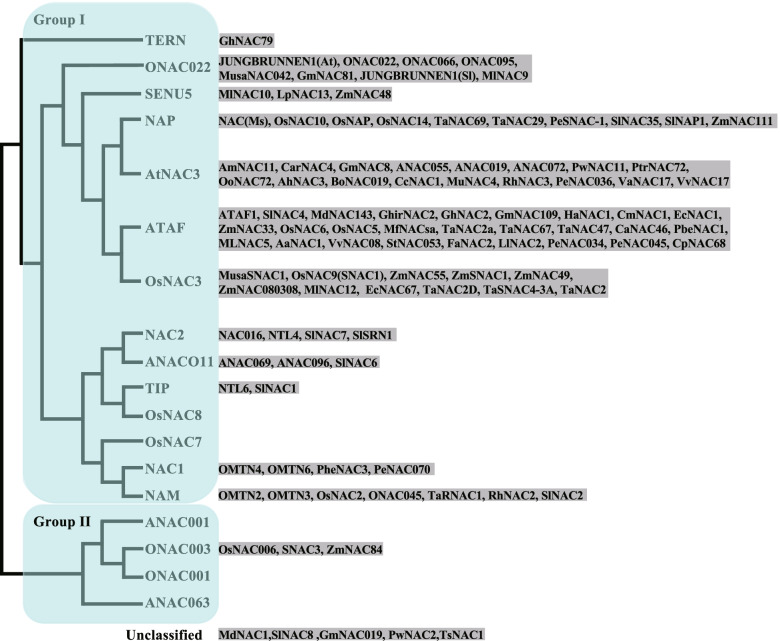
NAC family TFs involved in drought tolerance. The phylogenetic tree was drawn according to (Ooka et al. 2003). 105 NAC genes belonging to 14 subgroups (including unclassified) have been studied to regulate drought tolerance

Typical NAC [No apical meristem (NAM), Arabidopsis transcription activation factor (ATAF), Cup-shaped cotyledon (CUC)] family TFs contain a conserved N-terminal NAC domain that is involved in DNA binding and dimerization, and a potential C-terminal transcriptional regulatory (TR) domain which has either activator or repressor function (Puranik et al. 2012). NAC proteins bind to various stress responsive and non-responsive NACRS (NAC recognition sequence) to regulate downstream gene expression. NAC TFs can be classified into two large groups which are further divided into 18 subgroups (Ooka et al. 2003). Members in subgroups NAP, AtNAC3, ATAF, and OsNAC3 are designated as Stress-associated NAC (SNAC). Indeed, we found that the reported NAC TFs related to DT are more enriched in these four subgroups than the others (Fig. 1). One hundred five *NAC* genes belonging to 14 subgroups (including unclassified) were collected in this paper and 64 of them fall into four SNAC subgroups (Fig. 1, Table S1). Specifically, 27 *NAC* genes from 22 plant species are included in the ATAF subgroup (Table S1), suggesting functional conservation of this subgroup genes in drought response across the plant kingdom. Seventeen of the one hundred four *NAC* genes negatively regulate DT while the others are positive regulators. In addition, *NAC* genes in group II containing four subgroups are unlikely to regulate DT as few of them have been characterized to exert the function.

#### ERF family

**Fig. 2:**
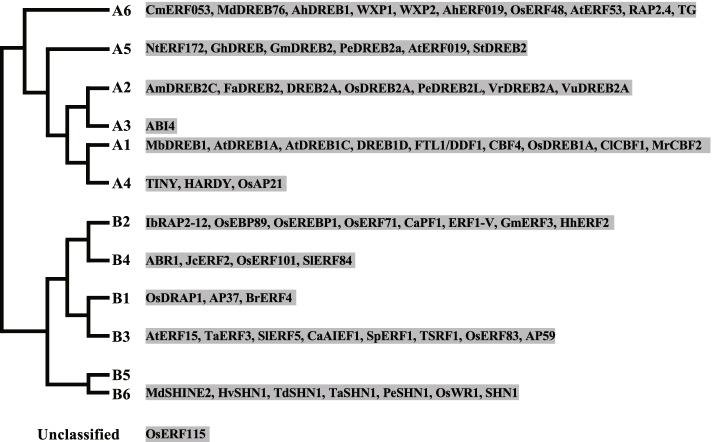
ERF family TFs involved in drought tolerance. The phylogenetic tree was drawn according to (Sakuma et al. 2002). 67 ERF genes assigned to all the subgroups except B-5 were discovered to play important roles in drought tolerance

ERF family TFs belong to AP2/ERF (APETALA2/ethylene-responsive element binding factors) superfamily which contain an AP2/ERF domain (Nakano et al. 2006). The ERF family is classified into two groups: group A (CBF/DREB proteins) and group B (ERF proteins) (Sakuma et al. 2002). Each group can be divided into six subgroups (A-1 to A-6 and B-1 to B-6). Group A proteins recognize A/GCCGAC (DRE/CRT; Dehydration-Responsive or C-Repeat element) whereas Group B proteins bind to the GCC-Box. 67 *ERF* genes assigned to all the subgroups except B-5 were discovered to play important roles in DT (Fig. 2, Table S1). It has long been considered that subgroup A-1 (CBF/DREB1) and subgroup A-2 (DREB2) ERF proteins are conserved regulators for improving abiotic stress tolerance (Agarwal et al. 2006). The sole member of subgroup A-3 ABI4 is an ABA-dependent transcriptional regulator which is associated with the specific CE1 element [CACC(G)], acting as either an activator or a repressor of gene expression (Wind et al. 2013). However, mutation of *ABI4* reduces plant resistance to drought (Khan et al. 2020). In addition to these prominent DT genes, the other ERF TFs including group B proteins could equally contribute to DT (Fig. 2). Interestingly, overexpression of the homologs of *SHN1* from seven plant species could confer DT in these plants, suggesting this gene to be a common potential DT regulator in plants (Table S1). Besides, only 1 of 67 ERF proteins, OsEBP89 in rice, has been proved to be a negative regulator of DT, as mutation of *OsEBP89* leads to enhanced plant resistance to drought (Table S1) (Zhang et al. 2020b).

#### WRKY family

**Fig. 3:**
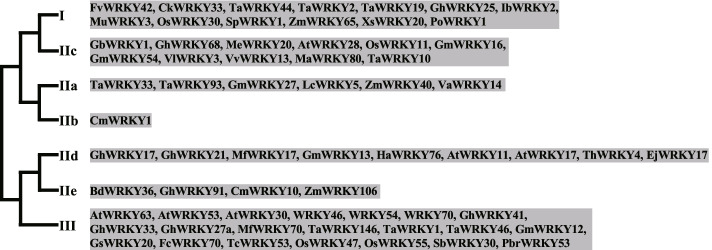
WRKY family TFs involved in drought tolerance. The phylogenetic tree was drawn according to (Rushton et al. 2010). 65 WRKY genes covering all the seven WRKY subfamilies have been revealed to participate in drought tolerance

WRKY family TFs possess one or two WRKY domains, about 60 amino acid residues with the WRKYGQK sequence followed by a C2H2 or C2HC zinc finger motif (Wu et al. 2005). The cognate binding site of WRKY domain is the W box (TTGACC/T) which could be recognized by many WRKY TFs. However, a few studies reported that some WRKY proteins also bound to non-W box cis-elements (Rushton et al. 2010). Sixty-five WRKY genes covering all the seven WRKY subfamilies have been revealed to participate in DT, and relatively more genes in subgroup I, IIc and III have been characterized (Fig. 3, Table S1). Thirteen WRKY genes in subgroup I, IIc, IId and II are negatively related to DT.

#### bZIP family

**Fig. 4:**
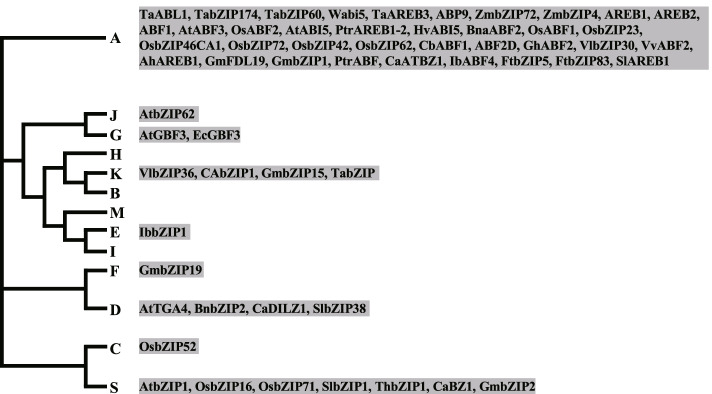
bZIP family TFs involved in drought tolerance. The phylogenetic tree was drawn according to (Droge-Laser et al. 2018). 58 bZIP proteins identified to play roles in drought tolerance fall in nine groups

bZIP (the basic leucine zipper) TFs are defined by a basic region for DNA-binding and a leucine zipper motif for dimerization (Jakoby et al. 2002). They preferentially bind to DNA sequences with an ACGT core such as the A-box (TACGTA), C-box (GACGTC) and G-box (CACGTG) (Jakoby et al. 2002). According to the basic region and additional conserved motifs, 13 groups of bZIP proteins were defined (Droge-Laser et al. 2018). The ABA signaling-engaged ABI5, ABF1, ABF2/AREB1, ABF3, ABF4/AREB2 and AREB3 belong to group A. Notably, 37 out of 58 bZIP proteins that have been identified to play important roles in DT fall in this group (Fig. 4, Table S1). Overexpression of *AtABF3* in Arabidopsis, Medicago and cotton increases plant DT (Kerr et al. 2018; Wang et al. 2016b; Yoshida et al. 2010), demonstrating conserved function of this group genes in plant response to drought. However, the homologs of ABI5 seem to function distinctly in different plant species for DT. Both overexpression of wheat *Wabi5* in tobacco and ectopic expression of Arabidopsis *AtABI5* in cotton could enhance plant resistance to drought (Kobayashi et al. 2008; Mittal et al. 2014), whereas drought-tolerant phenotype was observed in barley *hvabi5.d* mutant carrying G1751A transition (Collin et al. 2020). The negative role of HvABI5 in barley DT was explained by the proposal issued by authors that it might be involved in the feedback regulation of ABA biosynthesis. Additional six bZIP genes were testified to be negative regulators of DT, as plants with overexpression of these genes is more sensitive to drought treatment (Table S1).

#### MYB family

**Fig. 5:**
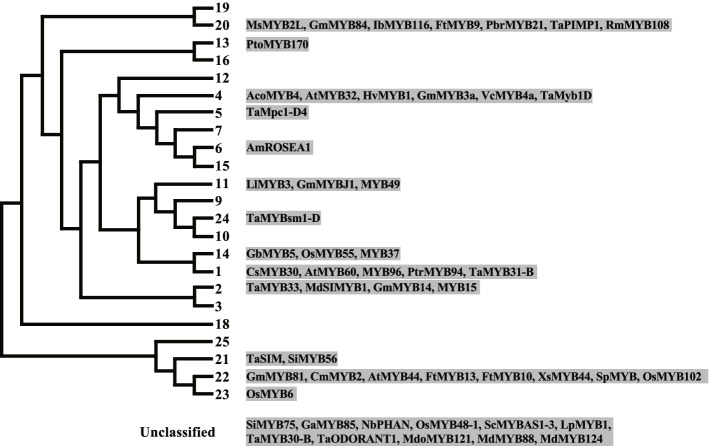
MYB family TFs involved in drought tolerance. The phylogenetic tree was drawn according to (Dubos et al. 2010). Functional studies on 54 R2R3-MYBs assigned to 13 subgroups as well as unidentified group have been performed for drought tolerance

MYB (myeloblastosis) proteins are identified through a highly conserved DNA-binding domain: the MYB domain, which consists of up to four imperfect amino acid sequence repeats (R) of about 52 amino acids (Dubos et al. 2010). They can be divided into four classes based on the number of adjacent repeats: 4R-MYB, R1R2R3-type MYB (3R-MYB), R2R3-MYB and MYB-related. Plant MYB genes mostly encode R2R3-MYB, which bind to MYB-core [C/T]NGTT[G/T] and AC-rich elements (Millard et al. 2019). Plant R2R3-MYB can be divided into 25 subgroups according to MYB domain and C-terminal motifs (Millard et al. 2019). To date, functional studies on 54 R2R3-MYBs assigned to 13 subgroups as well as unidentified group have been performed for DT (Fig. 5, Table S1). These studies unraveled that seven *R2R3-MYB* genes were negatively engaged in plant tolerance to drought.

#### HD-zip

HD-Zip (homeodomain-leucine zipper) protein is composed of a homeodomain (HD) and an immediately downstream leucine zipper motif (LZ), and classified into four subfamilies on the basis of HD-Zip domain conservation and additional conserved motifs (Ariel et al. 2007). It seems that HD-Zip TFs in each subfamily have distinct DNA-binding specificity (Ariel et al. 2007). HD-Zip I and HD-Zip II proteins forming dimers recognize CAAT(A/T)ATTG and CAAT(C/G)ATTG respectively. The binding sites of HD-Zip IV proteins are more divergent despite TAAA core is present in their target sequences. HD-Zip III protein binding site is less characterized than the other subfamilies. Eighteen HD-Zip genes encoding 11 HD-Zip I, four HD-Zip II and three HD-Zip IV proteins are implicated in plant DT (Table S1), echoing the importance of HD-Zip I ZFs in the regulation of plant drought stress response. Among these genes, rice *Oshox22* and Arabidopsis *ABIG1*, *HAT1* and *HAT3* negatively regulate drought response (Zhang et al. 2012; Tan et al. 2018; Liu et al. 2016). Furthermore, *HAT1* and *HTA3* function redundantly since plant DT is not affected by single mutation of them while dramatically increased by double mutation (Tan et al. 2018).

#### ZnF family

ZnF (zinc finger) proteins are classified into several different types based on the number and order of the Cys and His residues that bind the Zinc ion in the secondary structure of the finger (Ciftci-Yilmaz and Mittler 2008). C2H2-type is one of the most abundant ZnF proteins in eukaryotes, which is mainly classified into three sets (A, B and C) (Ciftci-Yilmaz and Mittler 2008). Plant-specific Set C can be further divided into three subsets (C1, C2 and C3). We found 15 C2H2 zinc finger proteins involved in DT, all of which belong to C1 subset and most are concentrated in subclass C1–2i (Table S1). Two genes, Rice *DST* and soybean *GmZFP3*, are negative regulators of DT (Cui et al. 2015; Huang et al. 2009; Zhang et al. 2016). In addition to C2H2 zinc finger proteins, seven C3H type zinc finger proteins, three Di-19 family zinc finger proteins, two BBX family proteins and four others are also implicated in the regulation of DT (Table S1).

#### bHLH family

bHLH (basic/helix-loop-helix) family proteins contain the conserved bHLH domain, which consists of N-terminal basic region (15 to 20 residues rich in basic amino acids) for DNA-binding and HLH region (two amphipathic a-helices linked by a loop region) for protein-protein interaction (Toledo-Ortiz et al. 2003). The so-called core E-box hexanucleotide consensus sequence 5′-CANNTG-3′ is recognized by the bHLH proteins (Toledo-Ortiz et al. 2003). Based on phylogenetic analysis the plant bHLH proteins could be classified into 32 subfamilies (Carretero-Paulet et al. 2010). Twenty-four bHLH genes belonging to 13 subfamilies have been functionally characterized for DT (Table S1). Only three *BEE* genes in Arabidopsis play negative roles in DT, mutation of which simultaneously (triple mutant) enhanced drought resistance.

#### ASR family

The ASR (abscisic acid, stress, ripening induced) protein, albeit absent in Arabidopsis, was initially screened from tomato leaves under water-stress conditions (Gonzalez and Iusem 2014). It might have either chaperone-like function or transcription factor activity (Gonzalez and Iusem 2014). The latter has been proved by its ability to bind DNA directly. Several downstream target genes regulated by ASR proteins in some species have also been identified in vivo, further supporting its regulatory role in gene transcription. The ASR-binding DNA motif identified in rice and tomato is conserved with consensus core sequence GCCCA (Arenhart et al. 2014; Ricardi et al. 2014). Seventeen ASR proteins have been reported to be involved in DT regulation, all of which are positive regulators (Table S1).

#### NF-Y family

Nuclear factor Y (NF-Y) TF is formed by three subunits: NF-YA, NF-YB and NF-YC (Chaves-Sanjuan et al. 2021). The histone fold domain (HFD) of NF-YB and NF-YC mediates their heterodimerization, which produces a molecular scaffold for NF-YA interaction. The NF-Y DNA target is the CCAAT box, recognized by NF-YA. The heterodimer of NF-YB and NF-YC can also bind DNA but in a non-sequence-specific manner (Chaves-Sanjuan et al. 2021). All of the studied NF-Y TFs including seven NF-YAs, four NF-YBs and one NF-YC are positive regulators of DT (Table S1).

#### HSF family

HSF (Heat shock factor) proteins, which bind the conserved cis-acting (5′-nGAAn-3′) heat shock elements (HSE), transcriptionally regulate heat shock protein (Hsp) genes to play a central role in the heat stress response (Nagaraju et al. 2015). Numerous publications document that HSP also affects other abiotic stresses as well as biotic stress. HSF genes can be grouped into three classes: A, B and C. Nine class A HSF proteins participate in the regulation of DT (Table S1). Simultaneous mutation of Arabidopsis *HSFA6a* and *HSFA6b* leads to the enhancement of drought resistance while single mutation does not, suggesting cooperative and negative effect of these two genes on DT regulation (Wenjing et al. 2020).

#### Others

Genes in some families, whose members are always considered as important developmental regulators like *WOX*, *KNOX*, *GT2*, *BES/BZR* and *GRAS*, also function as DT regulators (Table S1). Some of them directly regulate the expression of genes involved in drought response or genes encoding enzymes for scavenging reactive oxygen species (ROS). And the others developmentally control plant architecture to influence DT. For example, repression of *SDD1* by Arabidopsis *GTL1* contributes to high abaxial stomatal density resulting in lower water use efficiency (Yoo et al. 2010). Overexpression of *PagKNAT2/6b* causes shorter internode length and smaller leaf size by inhibiting GA biosynthesis (Song et al. 2021) but improves drought resistance.

#### Regulatory network established by TFs

In response to drought, the regulatory network could be built by direct interaction of TFs to regulate common targets and mutual regulation of TFs to amplify or compromise drought signal. The direct interaction or mutual regulation summarized here has been experimentally evidenced by protein-protein interaction assays or protein-DNA binding assays in vitro or in vivo. The NAC family proteins are the most reported TFs to associate with other factors, involving both hetero-dimerization within family and interaction with other family TFs such as ERF family (LlDREB1, PbeDREB1, PbeDREB2A and DREB2A in *Picea (P.) wilsonii*), bZIP family (ABF2, ABF3 and ABF4), ZnF family (GmDi19–3), ZFHD family (TsHD1 and ZFHD4) (Table 1). Interestingly, by binding to each other GmNAC81 and GmNAC30 synergistically either activate or repress common target genes expression, which would depend on the conformational assembly of the two TFs at their binding site (Mendes et al. 2013). Majority of TFs interaction mediate their cooperative regulation of target genes except TINY and BES1 in Arabidopsis. Although interacting with each other, TINY and BES1 oppositely regulate a significant set of drought-induced and growth-related genes by inhibiting each other’s activities under different conditions (Xie et al. 2019). Under normal condition, BES1 promotes growth-related genes and represses drought responsive genes while TINY’s activity is inhibited. Under drought condition, TINY is induced to activate drought response and inhibit plant growth by counteracting BES1 functions. Three WRKY proteins (WRKY46, WRKY54 and WRKY70) also interact with BES1 but in a cooperative way to regulate BR-mediated plant growth and drought response (Chen et al. 2017).

**Table 1:** The interaction proteins of drought-responsive transcription factors

Function of interaction protein	TF name	Species	Interaction protein	References
TFs	ANAC096	Arabidopsis	ABF2 and ABF4	(Xu et al. 2013)
	GmNAC81	Soybean	GmNAC30	(Mendes et al. 2013)
	PeSNAC-1	Moso bamboo	PeSNAC-2/4 and PeNAP-1/4/5	(Hou et al. 2020)
	GmNAC8	Soybean	GmDi19–3	(Yang et al. 2020a)
	PwNAC11	*Picea wilsonii*	ABF3, DREB2A	(Yu et al. 2021)
	HaNAC1	*Haloxylon ammodendron*	AtNAC32	(Gong et al. 2020a)
	PbeNAC1	Pyrus	PbeDREB1, PbeDREB2A	(Jin et al. 2017a)
	LlNAC2	Lily	LlDREB1, ZFHD4	(Yong et al. 2019b)
	TsNAC1	*Thellungiella halophile*	TsHD1	(Liu et al. 2019a)
	TINY	Arabidopsis	BES1	(Xie et al. 2019)
	GmWRKY27	Soybean	GmMYB174	(Wang et al. 2015)
	WRKY46	Arabidopsis	BES1	(Chen et al. 2017)
	WRKY54	Arabidopsis	BES1	(Chen et al. 2017)
	WRKY70	Arabidopsis	BES1	(Chen et al. 2017)
	TaHDZipI-5	Wheat	TaHDZipI-3	(Yang et al. 2018)
	DST	Rice	DCA1	(Cui et al. 2015)
	CmBBX19	Chrysanthemum	CmABF3	(Xu et al. 2020)
	PdNF-YB21	Poplar	PdFUS3	(Zhou et al. 2020)
Kinases	NTL6	Arabidopsis	SnRK2.8	(Kim et al. 2012)
	ZmNAC84	Maize	ZmCCaMK	(Zhu et al. 2016)
	AtERF7	Arabidopsis	PKS3, AtSin3	(Song et al. 2005)
	OsEBP89	Rice	SnRK1alph	(Zhang et al. 2020b)
	RAP2.6	Arabidopsis	CDK8 and SnRK2.6	(Zhu et al. 2020b)
	OsWRKY30	Rice	OsMPK3, OsMPK4, OsMPK7, OsMPK14, OsMPK20–4, and OsMPK20–5,	(Shen et al. 2012)
	GhWRKY59	Cotton	GhMAP3K15-GhMKK4-GhMPK6	(Li et al. 2017a)
	OsWRKY55	Rice	OsMPK7, OsMPK9, OsMPK20–1, and OsMPK20–4	(Huang et al. 2021)
	ABF1	Arabidopsis	AtCPK4, AtCPK11	(Zhu et al. 2007)
	AREB1	Arabidopsis	SRK2D/SnRK2.2	(Yoshida et al. 2015)
	AREB2	Arabidopsis	SRK2D/SnRK2.2, AtCPK4, AtCPK11	(Yoshida et al. 2015; Zhu et al. 2007)
	ABF3	Arabidopsis	SRK2D/SnRK2.2, AtCPK6	(Yoshida et al. 2015; Zhang et al. 2020a)
	ABI5	Arabidopsis	AtCPK6	(Zhang et al. 2020a)
	OsbZIP23	Rice	SAPK2	(Zong et al. 2016)
	OsbZIP46CA1	Rice	SAPK6	(Chang et al. 2017)
	CbABF1	Cryophyte	CbSnRK2.6	(Yue et al. 2019)
	OsbZIP62	Rice	SAPKs	(Yang et al. 2019)
	FtbZIP5	Buckwheat	FtSnRK2.6	(Li et al. 2020)
	MYB44	Arabidopsis	MPK3	(Persak and Pitzschke 2014)
	HAT1	Arabidopsis	SnRK2.3	(Tan et al. 2018)
	Di19	Arabidopsis	CPK11	(Liu et al. 2013)
	TINY	Arabidopsis	BIN2	(Xie et al. 2019)
	WRKY46	Arabidopsis	BIN2	(Chen et al. 2017)
	WRKY54	Arabidopsis	BIN2	(Chen et al. 2017)
	WRKY70	Arabidopsis	BIN2	(Chen et al. 2017)
	SlVOZ1	Tomato	SlOST1	(Chong et al. 2022)
UPS	ABI5	Arabidopsis	DWA1/DWA2, KEG, ABD1	(Chen et al. 2013; Lee et al. 2010; Seo et al. 2014)
	ABF1	Arabidopsis	KEG	(Chen et al. 2013)
	ABF3	Arabidopsis	KEG	(Chen et al. 2013)
	DREB2A	Arabidopsis	DRIP1	(Qin et al. 2008)
	MdNAC143	Apple	MdBT2	(Ji et al. 2020)
	AtERF53	Arabidopsis	RGLG2	(Cheng et al. 2012)
	OsWRKY11	Rice	ubiquitin-proteasome	(Lee et al. 2018)
	CaATBZ1	*Capsicum Annuum*	CaASRF1	(Joo et al. 2019b)
	CaDILZ1	*Capsicum Annuum*	CaDSR1	(Lim et al. 2018)
	ROC4	Rice	DHS	(Wang et al. 2018)
Others	CcNAC1	Jute	KCS	(Zhang et al. 2021)
	PwNAC2	*Picea wilsonii*	PwRFCP1	(Zhang et al. 2018)
	ABI4	Arabidopsis	PWR, HDA9	(Khan et al. 2020; Baek et al. 2020)
	OsDRAP1	Rice	OsCBSX3	(Huang et al. 2018)
	MeWRKY20	Cassava	MeHSP90s	(Wei et al. 2020)
	PtrAREB1–2	Poplar	ADA2b-GCN5	(Li et al. 2019a)
	GmMYB81	Soybean	GmSGF14l	(Bian et al. 2020)
	ZFP182	Rice	ZIURP1	(Huang et al. 2012)
	IbC3H18	Sweetpotato	IbPR5	(Zhang et al. 2019)
	MfNACsa	Medicago	APT1	(Duan et al. 2017)

Although DREB proteins including DREB1 and DREB2 are considered as master regulators of both drought and cold response, their direct downstream genes proved experimentally have seldom been reported. By contrast, DREB proteins are likely to serve as common targets regulated by multiple family TFs like NAC (JUNGBRUNNEN1 and ONAC066), WRKY (TaWRKY19 and GhWRKY59), bZIP (AREB1, AREB2 and AtABF3), ZnF (Os12g38960， Os03g32230 and Os11g47630), MYB (AtMYB32), bHLH (ZjICE2, ZmbHLH124 and ZmPTF1), NF-Y (GmNFYA5) and WOX (OsWOX13) (Table 2). Among these TFs, only three ZnF family proteins and AtMYB32 are negative regulators of *DREB* genes (Figueiredo et al. 2012; Li et al. 2021b). The activation of *DREB* genes by AREB1, AREB2 and AtABF3 couples ABA-dependent and ABA-independent pathways in response to drought (Kim et al. 2011). In addition, ABA-dependent bZIP family regulators could be regulated by various TFs. For example, *AREB1* is activated by AtWRKY63 while repressed by drought-responsive NAC016 to form a feed-forward loop (Ren et al. 2010; Sakuraba et al. 2015). AtMYB32 is responsible for the activation of *ABI5* while AtWRKY40 and AtRAV1 are the negative regulators of *ABI5* (Li et al. 2021b; Liu et al. 2012; Feng et al. 2014).

**Table 2:** The direct downstream genes of drought-responsive transcription factors

Function of downstream genes	Protein name	Species	Direct target genes	Transcriptional activity	References
TFs	JUNGBRUNNEN1	Tomato	*DREB1, DREB2*	Activation	(Thirumalaikumar et al. 2018)
	ONAC066	Rice	*OsDREB2A*	Activation	(Yuan et al. 2019)
	NAC016	Arabidopsis	*AREB1*	Repression	(Sakuraba et al. 2015)
	TaWRKY2	Wheat	*STZ*	Activation	(Niu et al. 2012)
	TaWRKY19	Wheat	*DREB2A*	Activation	(Niu et al. 2012)
	GmWRKY27	Soybean	*GmNAC29*	Repression	(Wang et al. 2015)
	MdWRKY31	Apple	*MdRAV1*	Repression	(Zhao et al. 2019)
	GhWRKY91	Cotton	*GhWRKY17*	Activation	(Gu et al. 2019)
	AtWRKY63	Arabidopsis	*ABF2*	Activation	(Ren et al. 2010)
	GhWRKY59	Cotton	*GhDREB2*	Activation	(Li et al. 2017a)
	OsWRKY55	Rice	*OsAP2–39*	Activation	(Huang et al. 2021)
	CaWRKY70	Chickpea	*CaHDZ12*	Repression	(Sen et al. 2017)
	AREB1	Arabidopsis	*DREB2A*	Activation	(Kim et al. 2011)
	AREB2	Arabidopsis	*DREB2A*	Activation	(Kim et al. 2011)
	AtABF3	Arabidopsis	*DREB2A*	Activation	(Kim et al. 2011)
	PtrAREB1–2	Poplar	*PtrNAC006, PtrNAC007, PtrNAC120*	Activation	(Li et al. 2019a)
	OsABF1	Rice	*OsbZIP23, OsbZIP46, OsbZIP72*	Activation	(Zhang et al. 2017)
	AtMYB32	Arabidopsis	*ABI3, ABI4, ABI5, CBF4*	Activation/ Repression	(Li et al. 2021b)
	AtWRKY40	Arabidopsis	*ABI5*	Repression	(Liu et al. 2012)
	AtHB13	Arabidopsis	*JUNGBRUNNEN1*	Activation	(Ebrahimian-Motlagh et al. 2017)
	Os12g38960	Rice	*OsDREB1B*	Repression	(Figueiredo et al. 2012)
	Os03g32230	Rice	*OsDREB1B*	Repression	(Figueiredo et al. 2012)
	Os11g47630	Rice	*OsDREB1B*	Repression	(Figueiredo et al. 2012)
	ZjICE2	*Zoysia Japonica*	*ZjDREB1*	Activation	(Zuo et al. 2020)
	ZmbHLH124	Maize	*ZmDREB2A*	Activation	(Wei et al. 2021)
	*ThMYC6*	Tamarix Hispida	*ThbZIP1*	Activation	(Ji et al. 2013)
	ZmPTF1	Maize	*CBF4, ATAF2/NAC081, NAC30*	Activation	(Li et al. 2019b)
	ZmNF-YA3	Maize	*bHLH92*	Repression	(Su et al. 2018)
	GmNFYA5	Soybean	*GmDREB2, GmbZIP1*	Activation	(Ma et al. 2020)
	SiARDP	Foxtail Millet	*SiASR4*	Activation	(Li et al. 2016)
	OsWOX13	Rice	*OsDREB1A, OsDREB1F*	Activation	(Minh-Thu et al. 2018)
ABA metabolism and signaling	ATAF1	Arabidopsis	*NCED3*	Activation	(Jensen et al. 2013)
	GhirNAC2	Cotton	*GhNCED3a/3c*	Activation	(Shang et al. 2020)
	WRKY57	Arabidopsis	*NCED3*	Activation	(Jiang et al. 2012)
	MeWRKY20	Cassava	*MeNCED5*	Activation	(Wei et al. 2020)
	MaWRKY80	Banana	*MaNCED*	Activation	(Liu et al. 2020a)
	PbrWRKY53	Pyrus	*PbrNCED1*	Activation	(Liu et al. 2019b)
	OsbZIP23	Rice	*OsPP2C49, OsNCED4*	Activation	(Zong et al. 2016)
	MdMYB88	Apple	*NCED3*	Activation	(Xie et al. 2021)
	HAT1	Arabidopsis	*ABA3 and NCED3*	Repression	(Tan et al. 2018)
	ZmPTF1	Maize	*NCED*	Activation	(Li et al. 2019b)
	PdNF-YB21	Poplar	*PdNCED3*	Activation	(Zhou et al. 2020)
	GmWRKY54	Soybean	*PYL8, SRK2A*	Activation	(Wei et al. 2019)
	GhWRKY21	Cotton	*GhHAB*	Activation	(Wang et al. 2020)
	ATHB7	Arabidopsis	*PP2C, PYL5, PYL8*	Activation	(Valdes et al. 2012)
	ATHB12	Arabidopsis	*PP2C, PYL5, PYL8*	Activation	(Valdes et al. 2012)
	OsABF1	Rice	*OsPP48, OsPP108*	Activation	(Zhang et al. 2017)
	bHLH122	Arabidopsis	*CYP707A3*	Repression	(Liu et al. 2014b)
	OsNAC2	Rice	*OsSAPK1*	Repression	(Shen et al. 2017)
ROS-related	NTL4	Arabidopsis	*AtrbohC, AtrbohE*	Activation	(Lee et al. 2012)
	ZmNAC84	Maize	*SOD2*	Activation	(Han et al. 2021)
	NtERF172	tobacco	*NtCAT*	Activation	(Zhao et al. 2020)
	TaBZR2	Wheat	*TaGST1*	Activation	(Cui et al. 2019)
	GmMYB84	Soybean	*GmRBOHB-1 and GmRBOHB-2*	Activation	(Wang et al. 2017)
Aquaporins	TG	Arabidopsis	*AtTIP1;1, AtTIP2;3, AtPIP2;2*	Activation	(Zhu et al. 2014)
	ASR1	Tomato	*Solyc10g054820*	Activation	(Ricardi et al. 2014)
LEA proteins	OsNAC2	Rice	*OsLEA3*	Repression	(Shen et al. 2017)
	OsWRKY11	Rice	*RAB21*	Activation	(Lee et al. 2018)
	TabHLH49	Wheat	*WZY2*	Activation	(Liu et al. 2020b)
Wax biosynthesis	OsWR1	Rice	*OsLACS2, OsFAE1’-L*	Activation	(Wang et al. 2012)
	PeSHN1	Poplar	*LACS2*	Activation	(Meng et al. 2019)
	MYB94	Arabidopsis	*wax biosynthetic genes*	Activation	(Lee et al. 2016b)
	MYB96	Arabidopsis	*wax biosynthetic genes*	Activation	(Lee et al. 2016b; Seo et al. 2011)
Polyamine biosynthesis	PtrNAC72	*Poncirus trifoliata*	*PtADC*	Repression	(Wu et al. 2016)
	FcWRKY70	*Fortunella Crassifolia*	*FcADC*	Activation	(Gong et al. 2015)
	PbrMYB21	Pyrus	*PbrADC*	Activation	(Li et al. 2017b)
	OsHSFA3	Rice	*OsADC*	Activation	(Zhu et al. 2020a)
Others	TaNAC69	Wheat	*chitinase, ZIM, glyoxalase I*	Activation	(Xue et al. 2011)
	ANAC019	Arabidopsis	*ERD1*	Activation	(Tran et al. 2004)
	ANAC055	Arabidopsis	*ERD1*	Activation	(Tran et al. 2004)
	ANAC072	Arabidopsis	*ERD1*	Activation	(Tran et al. 2004)
	PwNAC11	*Picea wilsonii*	*ERD1*	Activation	(Yu et al. 2021)
	OsNAC6	Rice	*NICOTIANAMINE SYNTHASE*	Activation	(Lee et al. 2017)
	ZmNAC49	Maize	*ZmMUTE*	Repression	(Xiang et al. 2021)
	RhNAC2	Rose	*RhEXPA4*	Activation	(Dai et al. 2012)
	OsERF48	Rice	*OsCML16*	Activation	(Jung et al. 2017)
	OsERF71	Rice	*OsCINNAMOYL-COENZYME A REDUCTASE1*	Activation	(Lee et al. 2016a)
	RAP2.6	Arabidopsis	*RD29A, COR15A*	Activation	(Zhu et al. 2020b)
	AtWRKY53	Arabidopsis	*QQS*	Activation	(Sun and Yu 2015)
	SbWRKY30	Sorghum	*SbRD19*	Activation	(Yang et al. 2020b)
	TaAREB3	Wheat	*RD29A, RD29B, COR15A, COR47*	Activation	(Wang et al. 2016a)
	OsbZIP71	Rice	*OsNHX1, COR413-TM1*	Activation	(Liu et al. 2014a)
	GmMYB14	Soybean	*GmBEN1*	Activation	(Chen et al. 2021)
	LlMYB3	Lily	*LlCHS2*	Activation	(Yong et al. 2019a)
	OsTF1L	Rice	*poxN/PRX38, Nodulin protein, DHHC4, CASPL5B1, AAA-type ATPase.*	Activation	(Bang et al. 2019)
	Di19	Arabidopsis	*PR1, PR2, PR5*	Activation	(Liu et al. 2013)
	PagKNAT2/6b	Poplar	*PagGA20ox1*	Repression	(Song et al. 2021)
	GTL1	Arabidopsis	*SDD1*	Repression	(Yoo et al. 2010)
	ANAC096	Arabidopsis	*RD29A*	Activation	(Xu et al. 2013)
	TaWRKY2	Wheat	*RD29B*	Activation	(Niu et al. 2012)
	TaWRKY19	Wheat	*Cor6.6*	Activation	(Niu et al. 2012)
	AREB1	Arabidopsis	*RD29B*	Activation	(Uno et al. 2000)
	AREB2	Arabidopsis	*RD29B*	Activation	(Uno et al. 2000)
	GmWRKY54	Soybean	*CIPK11, CPK3*	Activation	(Wei et al. 2019)
	SlVOZ1	Tomato	*SFT*	Activation	(Chong et al. 2022)

### Post-transcriptional regulation of TFs

In addition to transcriptional regulation by each other, TFs could subject post-transcriptional regulation including phosphorylation carried out by different kinds of kinases, degradation of TFs by ubiquitin-proteasome system (UPS) (Table 1), translocation of TFs from the cytoplasm to the nucleus, and post-transcriptional regulation by miRNA.

#### Phosphorylation of TFs

In ABA-dependent pathway ABA signal is transmitted to TFs like AREB1, AREB2 and ABF3 through phosphorylation of them by SNF1-Related Protein Kinase (SnRK)2 Protein Kinases (Yoshida et al. 2015). The phosphorylation of TFs contributes to full activation of their transcriptional activities. Calcium dependent protein kinases, CPK4 and CPK11 or CPK6, were also reported to mediate ABA responsive phosphorylation of ABFs and/or ABI5 to enhance transcriptional activities (Zhang et al. 2020a; Zhu et al. 2007). The phosphorylation of bZIP TFs by SnRK2 kinases has been observed in rice (OsbZIP23，OsbZIP46CA1，OsbZIP62) (Chang et al. 2017; Yang et al. 2019; Zong et al. 2016), Cryophyte (CbABF1) (Yue et al. 2019) and buckwheat (FtbZIP5) (Li et al. 2020). Most of these bZIP proteins belong to the same group as ABFs, suggesting the conserved mechanism for signal transmission in plants. Besides, some TFs in other families could also be substrates of the SnRK2 kinases although the effect of phosphorylation of these TFs seems to be different. The NAC TF NTL6 in Arabidopsis is phosphorylated by SnRK2.8, which however is required for the entrance of NTL6 into the nucleus (Kim et al. 2012). Phosphorylation of the Arabidopsis HD-Zip TF HAT1 by SnRK2.3 both destabilize and repress promoter-binding activity of HAT1 for negatively regulating HAT1 in response to drought (Tan et al. 2018). In rice, the fact that OsSnRK1α interacts and phosphorylates ERF family TF OsEBP89 has also been observed, despite that the effect of phosphorylation remains to be determined (Zhang et al. 2020b). Recently, it was demonstrated that phosphorylation of SlVOZ1 by SlOST1 promoted both stability and nuclear translocation of SlVOZ1 in tomato (Chong et al. 2022). In addition to SnRK2 and CPK, other kinases such as MAK kinases, GSK3-like kinases (BIN2), could be responsible for phosphorylation of TFs to affect their stability and transcriptional activity (Table 1).

#### Degradation of TFs

UPS is an important pathway for post-transcriptional regulation of gene expression and involved in several hormone signal transductions like GA, auxin, Brassinosteroids, strigolactone by degrading transcriptional repressors that are also negative regulators of signaling. The specificity of UPS is determined by E3 ligases which interact with and attach ubiquitins to target proteins. We found that most TFs regulated by UPS are positive regulators of DT and accordingly the corresponding E3 ligases are negatively engaged in DT (Table 1, Table S1). One exception is that a DT repressor CaAIBZ1 that is a bZIP TF is ubiquitinated by the E3 ligase CaASRF1 (Joo et al. 2019a). *CaASRF1* is induced by drought and targets CaAIBZ1 for ubiquitination, thus it positively modulates ABA signaling and drought response. However, two genes encoding E3 ligases, MdBT2 and CaDSR1, are also induced by drought although acting as negative regulators of DT (Ji et al. 2020; Lim et al. 2018). The induction of these genes might be dedicated for attenuating drought response by destabilizing TFs to generate a feedback loop. A feed-forward loop could also be established by translocation of E3 ligase, represented by RGLG2, from the plasma membrane to the nucleus induced by stress (Cheng et al. 2012).

#### Translocation

Translocation of TFs from the plasma membrane to the nucleus in response to drought also occurs. Under unstressed conditions, MfNACsa is targeted to the plasma membrane through S-palmitoylation at Cys26 in the endoplasmic reticulum/Golgi (Duan et al. 2017). Under drought stress, MfNACsa translocates to the nucleus through de-S-palmitoylation mediated by the thioesterase MtAPT1, whose encoding gene is rapidly induced by dehydration stress. NTL6 contain strong α-helical transmembrane motifs (TMs) in their C-terminal regions and are predicted to be membrane-associated (Kim et al. 2007). ABA and cold treatment induce NTL6 release from membrane, suggesting that both cleavage and phosphorylation of NTL6 are prerequisite for NTL6 to enter the nucleus to exert its regulatory function (Kim et al. 2007). Indeed, in Arabidopsis more than 190 TFs are predicted to be membrane-bound transcription factors (MTFs) (Seo et al. 2008). They are dormant by associated with the intracellular membranes and activated by proteolytic cleavage that might be stress responsive. This strategy could potentially transmitted stress signals from outside into the nucleus, but more evidences are required to explain how stress triggers proteolytic cleavage.

#### Post-transcriptional regulation by microRNAs

MicroRNAs (miRNAs) are encoded by endogenous genes, whose initial products are designated primary miRNAs (pri-miRNAs) (Yu et al. 2017). Pri-miRNAs are then cleaved to generate precursor-miRNAs (pre-miRNAs), which are finally processed to mature miRNAs. miRNAs repress gene expression by cleavage of RNAs or inhibition of translation, playing important roles in plant development and stress response. Genome-wide identification of differentially expressed miRNAs in response to drought has been performed in various plant species (Ren et al. 2021; Yu et al. 2020; Fan et al. 2020; Pegler et al. 2019; Liu et al. 2018; Ferdous et al. 2017; Hamza et al. 2016; Xie et al. 2015; Yin et al. 2014; Xie et al. 2014; Wang et al. 2014; Zhou et al. 2010), which would not be discussed here. Functional analysis of miRNAs targeting TFs involved in DT mainly focus on miR164 and miR169. The targets of miR164 are NAC family TFs belonging to NAC1 and NAM subgroup (Fang et al. 2014). Their targets seem to play similar roles in drought resistance. Overexpression of *OMTN2*, *OMTN3*, *OMTN4*, and *OMTN6* in rice leads to enhanced drought sensitivity at the reproductive stage, suggesting their negative effects on drought resistance. The contradicting conclusion concerning the function of *OsNAC2* were drawn by two groups. One group showed that *OsNAC2*-overexpressing transgenic plants exhibited drought sensitive phenotype while silencing of *OsNAC2* enhanced tolerance to drought (Shen et al. 2017). The other group revealed that overexpression of a miR164b-resistant *OsNAC2* mutant gene improved DT (Jiang et al. 2019). The negative effect on DT has also been observed for *PeNAC070*, a miR164 target in *Populus euphratica* (Lu et al. 2017). The miR169 or its targets, NF-YA genes, have been characterized in Arabidopsis, tomato, rapeseed and soybean (Ni et al. 2013; Li et al. 2008; Li et al. 2021a; Zhang et al. 2011). Arabidopsis *NFYA5*, soybean *GmNFYA3* and rapeseed *NF-YA8* are positive regulators of plant tolerance to drought stress. However, overexpression of tomato miR169 and soybean miR169c also enhanced DT (Yu et al. 2019; Zhang et al. 2011), demonstrating complicated roles of miR169 in the regulation of drought stress response.

### Downstream targets of TFs

Regulatory hub established by TFs receive input signals triggered by drought from outside of the nucleus and transmit output signals by regulating downstream gene expression for reaction. Here we summarized the direct downstream genes of TFs, whose promoter could be bound by drought-induced TFs in vitro or in vivo, and the genes which were only examined to transcriptionally change responding to mutation or altered expression of TF genes are not included. The function of the direct downstream genes entails ABA biosynthesis and signaling, ROS-related, aquaporin, LEA proteins, wax biosynthesis and others (Table 2). In particular, osmotic adjustment (OA) is an important biochemical mechanism helping plants to acclimate to drought conditions. Several organic compounds like sugars, proline, betaines and inorganic ions contributes to OA (Turner 2018). However, the TFs that have been experimentally proved to direct regulate the genes related to accumulation of these solutes in response to drought cannot be found out. The direct regulators of *P5CS1*, encoding a rate-limiting enzyme in proline biosynthesis, have been reported for their roles in salt tolerance (Dai et al. 2018; Verma et al. 2020).

#### ABA metabolism and signaling

The biosynthesis of ABA is rapidly induced by drought stress to elicit series of responses such as stomatal closure. It is generally accepted that in higher plants ABA is synthesized through carotenoids pathway by multi-step enzymatic reactions, in which NCEDs function as the key rate-limiting enzymes. ABA can be degraded by hydroxylation which is mediated by CYP707A subfamily monooxygenases (Ma et al. 2018). Until now various families TFs in different plant species have been reported to directly activate expression of *NCED* genes to increase ABA level, which are represented by NAC (ATAF1, GhirNAC2), WRKY (WRKY57, MeWRKY20, MaWRKY80, PbrWRKY53), MYB (MdMYB88), bZIP (OsbZIP23), bHLH (ZmPTF1) and NF-Y (PdNF-YB21) (Table 2). The bZIP TF, HAT1, was identified to be the repressor of *NCED3* in Arabidopsis (Tan et al. 2018). The fact that mutation or over-expression of *HAT1* affects plant sensitivity to ABA indicates that it also negatively regulates ABA signaling although the target gene is not clear. Induction of elevated ABA level by stresses could also be achieved via repressing expression of *CYP707A*. bHLH122, strongly induced by drought, NaCl and osmotic stresses but not ABA treatment, directly binds to the promoter of *CYP707A3* to repress its expression (Liu et al. 2014b).

ABA signaling is transmitted by ABA receptors RCARs/PYR1/PYLs, PP2Cs, SnRK2s and specific TFs (Zhu 2016). In the absence of ABA, PP2Cs interact with SnRK2s to inhibit their activity by dephosphorylation of multiple Ser/Thr residues in the activation loop. ABA perception by the RCAR/PYR1/PYL proteins releases the PP2C-mediated inhibition of SnRKs’ activity The released SnRK2s are activated through autophosphorylation and subsequently phosphorylate downstream substrates including TFs as described above. GmWRKY54, AtHB7 and AtHB12 are directly involved in the activation of *PYL* genes. The *SnRK2* gene is activated by GmWRKY54 in soybean and repressed by OsNAC2 in rice. Although PP2C genes are negative regulators of ABA signaling, they can be transcriptionally stimulated by multiple TFs induced by drought or ABA such as OsbZIP23, OsABF1, GhWRKY21, AtHB7 and AtHB12 (Table 2). The stimulation of PP2C genes was proposed to form a feedback loop for mitigating the intensity of ABA signal.

#### ROS-related

Under normal conditions, the basal level of Reactive oxygen species (ROS), which induces redox signals that regulates numerous cellular processes, is balanced by generation in various subcellular organelles such as chloroplasts, mitochondria, peroxisomes and apoplast and scavenging by antioxidative systems involving enzymes like SOD, CAT, APX and non-enzymatic antioxidants such as ascorbate (ASC), glutathione (GSH) and carotenoids (Farooq et al. 2019; Waszczak et al. 2018). Under stress conditions, increased ROS concentrations incurs cellular damage by oxidizing macromolecular such as proteins, DNA and lipids (Farooq et al. 2019). The plasma membrane-localized NADPH oxidases termed respiratory burst oxidase homologs (RBOHs) are the major ROS producers in apoplast (Waszczak et al. 2018). In Arabidopsis, NTL4, a NAC TFs, is responsible for coupling ROS production to drought-induced leaf senescence via facilitating expression of *AtrbohC* and *AtrbohE* directly (Lee et al. 2012). However, in soybean a hypothesis was issued that up-regulation of *GmRBOHB-1* and *GmRBOHB-2* by GmMYB84 improves DT by inducing SOD/POD/CAT activity due to the increased H2O2 contents (Wang et al. 2017). The elevated production of ROS scavenging enzymes SOD and CAT can be induced by drought stress through transcriptional activation of TFs. For example, ZmNAC84 directly promotes *ZmSOD2* expression to enhance maize DT (Han et al. 2021). NtERF172 positively regulates *NtCAT* expression to confer tobacco drought resistance (Zhao et al. 2020). In addition, *T. aestivum glutathione s-transferase-1* (*TaGST1)*, which functions positively in scavenging drought-induced ROS, is activated by TaBZR2, a positive regulator of BR signaling (Cui et al. 2019).

#### Aquaporins

Aquaporins (AQPs) are transmembrane channels for water and some solutes transportation. Based on their intracellular locations and sequence similarities, AQPs can be divided into seven subfamilies, among which the plasma membrane intrinsic proteins (PIPs) and tonoplast intrinsic proteins (TIPs) are considered to be the major AQPs mediated water uptake in roots (Afzal et al. 2016). However, the role of PIPs and TIPs in DT is intricate. Although most of *PIP* genes are down-regulated in response to drought, silencing of *PIP* genes in different plant species decreased drought resistance possibly resulted from decreased root hydraulic conductivity (Lpr) or transpiration rates (Afzal et al. 2016). This is supported by the evidence that overexpression of *AQP* genes enhances drought resistance. Three Aquaporin Genes: *AtTIP1;1*, *AtTIP2;3*, and *AtPIP2;2* are directly activated by a *ERF* gene, *TG* (Zhu et al. 2014). The vitrified leaf phenotype was observed in both *35S:TG* and *35S:AtTIP1;1* plants possibly as a result of excess water accumulated in the intercellular spaces. Whether the vitrified leaf phenotype is related to enhanced DT is yet to be clarified. The aquaporin gene, *Solyc10g054820.1*, was screened as a direct target of tomato ASR1 by ChIP-seq experiment. Expression of this gene in ASR1-silenced lines is reduced (Ricardi et al. 2014).

#### LEA proteins

Late embryogenesis abundant (LEA) proteins were first identified in cotton (*Gossypium hirsutum*) seeds and were also found to be accumulated under stress conditions. Their precise roles in DT remain unknown, but they could serve as chaperones to protect macromolecules or as hydrophilic proteins to retain water (Bies-Etheve et al. 2008). LEA proteins were classified into nine groups based on the phylogenetic analysis in Arabidopsis (Hundertmark and Hincha 2008). Two dehydrin group genes, *RAB21* and *WZY2*, are directly activated by OsWRKY11 and TabHLH49 in rice and wheat respectively (Table 2). OsNAC5 and OsNAC2 antagonistically regulate expression of *OsLEA3*, which belongs to LEA_4 group (Table 2). Overexpression of *OsLEA3–1* and *OsLEA3–2*, also LEA_4 members, improved drought resistance, suggesting the functional importance of these proteins in plant DT (Duan and Cai 2012; Xiao et al. 2007).

#### Wax biosynthesis

The cuticle on the surface of plant, an extracellular hydrophobic layer, mainly contains cutin and cuticular wax (Lewandowska et al. 2020). Under drought conditions, it becomes thicker to reduce water loss accompanied with greater wax deposition. Cuticular wax is composed of very-long-chain fatty acids (VLCFAs), their esters, alcohols, aldehydes, alkanes and ketones. Wax biosynthesis consists of de novo biosynthesis of C16 and C18 fatty acids, fatty acid elongation (FAE) and wax production including alcohol-forming pathway and alkane-forming pathway (Yeats and Rose 2013; Lewandowska et al. 2020). In Arabidopsis, MYB96 and MYB94 redundantly regulate wax biosynthesis by targeting multiple wax biosynthetic genes such as *KCS1*, *KCS2*, *CER2*, *CER6*, *CER10*, *KCR1* for FAE, *CER3* and *WSD1* in alcohol-forming pathway and *CER4* in alkane-forming pathway (Lee et al. 2016b). LACS2, catalyzing formation of long-chain-acyl-CoA, is conservatively activated by SHN1 homologs in rice (OsWR1) and *Populus x euramericana* (PeSHN1) (Table 2).

#### Polyamine biosynthesis

Polyamines, including putrescine (Put), spermidine (Spd), and spermine (Spm), play important roles in plant development and stress tolerance (Shi and Chan 2014). The function of polyamines in abiotic stress tolerance may involve ion homeostasis, keeping water status, ROS homeostasis, Osmotic balance and other responses. The genes encoding enzymes involved in polyamine biosynthetic pathway are induced by various stresses. The arginine decarboxylase (ADC) catalyzes the first step of polyamine biosynthesis. Expression of *ADC* genes in *Fortunella Crassifolia*，*Pyrus Betulaefolia* and rice is promoted by FcWRKY70，PbrMYB21，OsHSFA3 respectively (Table 2). PtrNAC72 acts as a transcriptional repressor of *PtADC* to negatively regulate polyamine level in response to drought (Table 2).

#### Others

The other direct targets of drought-induced TFs are listed in Table 2, mainly including genes encoding lignin biosynthetic enzymes, proteases, GA biosynthetic enzyme, BR catabolic enzyme, expansin, stomatal development regulator, RD29A, RD29B and so on. Most of these regulations are found only in one species although how they are related to DT are elucidated. More evidences are required to prove whether they are common targets regulated by drought in different plant species. Interestingly, recent studies revealed that direct activation of *SINGLE FLOWER TRUSS* (*SFT*) by SlVOZ1 is essential for drought-accelerated flowering, known as a drought escape (DE) response (Chong et al. 2022).

### Concluding remarks

**Fig. 6:**
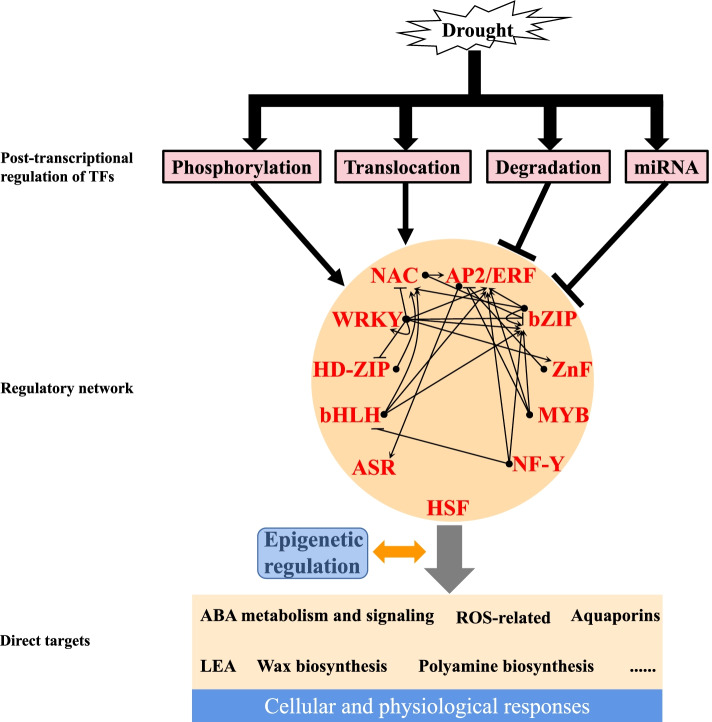
The diagram of drought signaling transmitted by regulatory network. Drought-induced post-transcriptional regulation of transcription factors (TFs) may function to transmit the signal to TFs by activating TFs such as phosphorylation and translocation of TFs, or to mitigate intensity of the signal by compromising TFs such as ubiquitin-proteasome system (UPS) mediated protein degradation and microRNA (miRNA). The regulatory network is built by mutual regulation of TFs (activation or repression) and interaction of TFs that is not shown in the figure to trigger cellular and physiological responses by directly regulating related genes. The regulation of TFs and downstream genes here refers to direct binding of TFs to promoters of target genes which has been experimentally proved to regulate their expression. How TFs and epigenetic regulation that mediates stress memory cooperate for fine tuning of drought-responsive genes will be of great interest in the future

In this review, we summarized the reported TFs that have been functionally analyzed for their roles in DT in multiple higher plants. These TFs are mainly distributed in 11 families, which form the regulatory network to receive signals possibly via post-transcriptional regulation and trigger cellular and physiological reactions by direct regulating expression of downstream genes (Fig. 6). There is still a long way to go for completing the interactive map of TFs. For the role of TFs in the transmission of drought signal, the key question is how they receive the signal from outside the nucleus. The phosphorylation of TFs could serve as one way but definitely not the only way. Besides, whether or not the drought-responsive pioneer TFs, which have the ability to recognize DNA sequence motifs exposed on the surface of a nucleosome (Zaret 2020), exist in plant is yet to be determined. As a matter of fact, the drought-responsive TF has been reported to interact with histone acetyltransferase (HAT) to regulate chromatin state of target genes for activation of these genes (Li et al. 2019a). The priming chromatin state is crucial for reactivation of genes during repeated stress, thus serving as epigenetic stress memory (Baurle and Trindade 2020). It is of great importance to understand how TFs and epigenetic regulators cooperated to mediate drought stress memory. Finally, identification and characterization of the TF genes that specifically induced by drought and could confer DT but do not affect plant development would provide theoretical basis for genetic improvement of crop drought resistance.

## Supplementary Information

**Additional file 1: Table S1.** The list of transcription factors (TFs) functionally involved in drought tolerance.

## Data Availability

Not applicable.

## Abbreviations

TF: Transcription factor
DT: Drought tolerance
NAC: No apical meristem (NAM), Arabidopsis transcription activation factor (ATAF), Cup-shaped cotyledon (CUC)
ERF: Ethylene-responsive element binding factor
bZIP: Basic leucine zipper
HD-Zip: Homeodomain-leucine zipper
ZnF: Zinc finger
bHLH: Basic/helix-loop-helix
ASR: Abscisic acid, stress, ripening induced
NF-Y: Nuclear factor Y
HSF: Heat shock factor
