# Supplementary material for: Regulatory network established by transcription factors transmits drought stress signals in plant
Source: Stress Biol. 2022 Jul 14;2(1):26. doi: 10.1007/s44154-022-00048-z (PMC10442052; doi:10.1007/s44154-022-00048-z)
Supplement: Supplementary file 1 — Additional file 1: Table S1. The list of transcription factors (TFs) functionally involved in drought tolerance. [file 44154_2022_48_MOESM1_ESM.docx]

**Table S1 The list of transcription factors (TFs) functionally involved in drought tolerance**

| **TF family** | **Group** | **TF name** | **Species** | **Gene manipulation** | **Recipient species** | **Effect on drought tolerance** | **Reference** |
| --- | --- | --- | --- | --- | --- | --- | --- |
| **NAC** | TERN | GhNAC79 | Cotton | Overexpression/Silence | Cotton | Enhanced/Reduced | (Guo et al. 2017) |
|  | ONAC022 | JUNGBRUNNEN1 | Arabidopsis | Overexpression | Arabidopsis | Enhanced | (Ebrahimian-Motlagh et al. 2017) |
|  |  | ONAC022 | Rice | Overexpression | Rice | Enhanced | (Hong et al. 2016) |
|  |  | ONAC066 | Rice | Overexpression/Silence | Rice | Enhanced/Reduced | (Yuan et al. 2019) |
|  |  | ONAC095 | Rice | Silence | Rice | Enhanced | (Huang et al. 2016b) |
|  |  | MusaNAC042 | Banana | Overexpression | Banana | Enhanced | (Tak et al. 2017) |
|  |  | GmNAC81 | Soybean | Overexpression | Soybean | Reduced | (Ferreira et al. 2020) |
|  |  | JUNGBRUNNEN1 | Tomato | Silence | Tomato | Reduced | (Thirumalaikumar et al. 2018) |
|  |  | MlNAC9 | Miscanthus | Overexpression | Arabidopsis | Enhanced | (Zhao et al. 2016b) |
|  | SENU5 | MlNAC10 | Miscanthus | Overexpression | Arabidopsis | Enhanced | (He et al. 2019) |
|  |  | LpNAC13 | Lily | Overexpression | Tobacco | Reduced | (Wang et al. 2020e) |
|  |  | ZmNAC48 | Maize | Overexpression | Arabidopsis | Enhanced | (Mao et al. 2021) |
|  | NAP | NAC | Medicago | Overexpression | Arabidopsis | Enhanced | (Wang 2013) |
|  |  | OsNAC10 | Rice | Overexpression | Rice | Enhanced | (Jeong et al. 2010) |
|  |  | OsNAP | Rice | Overexpression | Rice | Enhanced | (Chen et al. 2014) |
|  |  | OsNAC14 | Rice | Overexpression | Rice | Enhanced | (Shim et al. 2018) |
|  |  | TaNAC69 | Wheat | Overexpression | Wheat | Enhanced | (Xue et al. 2011) |
|  |  | TaNAC29 | Wheat | Overexpression | Arabidopsis | Enhanced | (Huang et al. 2015) |
|  |  | PeSNAC-1 | Moso bamboo | Overexpression | Rice | Enhanced | (Hou et al. 2020a) |
|  |  | SlNAC35 | Tomato | Overexpression | Tobacco | Enhanced | (Wang et al. 2016c) |
|  |  | SlNAP1 | Tomato | Overexpression | Tomato | Enhanced | (Wang et al. 2020c) |
|  |  | ZmNAC111 | Maize | Overexpression/Silence | Maize | Enhanced/Reduced | (Mao et al. 2015) |
|  | AtNAC3 | AmNAC11 | Ammopiptanthus mongolicus | Overexpression | Arabidopsis | Enhanced | (Pang et al. 2019) |
|  |  | CarNAC4 | Chickpea | Overexpression | Arabidopsis | Enhanced | (Yu et al. 2016b) |
|  |  | GmNAC8 | Soybean | Overexpression/Mutation | Soybean | Enhanced/Reduced | (Yang et al. 2020a) |
|  |  | ANAC055 | Arabidopsis | Overexpression/Mutation | Arabidopsis | Enhanced/Reduced | (Fu et al. 2018) |
|  |  | ANAC019 | Arabidopsis | Overexpression | Arabidopsis | Enhanced | (Tran et al. 2004) |
|  |  | ANAC072 | Arabidopsis | Overexpression | Arabidopsis | Enhanced | (Tran et al. 2004) |
|  |  | PwNAC11 | Picea wilsonii | Overexpression | Arabidopsis | Enhanced | (Yu et al. 2021) |
|  |  | PtrNAC72 | Poncirus trifoliata | Overexpression | Tobacco | Reduced | (Wu et al. 2016) |
|  |  | OoNAC72 | Oxytropis ochrocephala | Overexpression | Arabidopsis | Enhanced | (Guan et al. 2019) |
|  |  | AhNAC3 | Peanut | Overexpression | Tobacco | Enhanced | (Liu et al. 2013c) |
|  |  | BoNAC019 | Cabbage | Overexpression | Arabidopsis | Reduced | (Wang et al. 2018c) |
|  |  | CcNAC1 | Jute | Overexpression | Jute | Enhanced | (Zhang et al. 2021) |
|  |  | MuNAC4 | Horsegram | Overexpression | Groundnut | Enhanced | (Pandurangaiah et al. 2014) |
|  |  | RhNAC3 | Rose | Overexpression | Arabidopsis | Enhanced | (Jiang et al. 2014) |
|  |  | PeNAC036 | Poplar | Overexpression | Arabidopsis | Enhanced | (Lu et al. 2018) |
|  |  | VaNAC17 | Grape | Overexpression | Arabidopsis | Enhanced | (Su et al. 2020) |
|  |  | VvNAC17 | Grape | Overexpression | Arabidopsis | Enhanced | (Ju et al. 2020b) |
|  | ATAF | ATAF1 | Arabidopsis | Overexpression | Arabidopsis | Enhanced | (Wu et al. 2009b) |
|  |  | SlNAC4 | Tomato | Silence | Tomato | Reduced | (Zhu et al. 2014b) |
|  |  | MdNAC143 | Apple | Overexpression | Arabidopsis | Enhanced | (Ji et al. 2020) |
|  |  | GhirNAC2 | Cotton | Silence | Cotton | Reduced | (Shang et al. 2020) |
|  |  | GhNAC2 | Cotton | Overexpression | Arabidopsis/Cotton | Enhanced | (Gunapati et al. 2016) |
|  |  | GmNAC109 | Soybean | Overexpression | Arabidopsis | Enhanced | (Nguyen et al. 2019; Yang et al. 2019b) |
|  |  | HaNAC1 | Haloxylon ammodendron | Overexpression | Arabidopsis | Enhanced | (Gong et al. 2020) |
|  |  | CmNAC1 | Pumpkin | Overexpression | Arabidopsis | Enhanced | (Cao et al. 2017) |
|  |  | EcNAC1 | Finger millet | Overexpression | Tobacco | Enhanced | (Ramegowda et al. 2012) |
|  |  | ZmNAC33 | Maize | Overexpression | Arabidopsis | Enhanced | (Liu et al. 2019c) |
|  |  | OsNAC6 | Rice | Overexpression/Mutation | Rice | Enhanced/Reduced | (Nakashima et al. 2007; Lee et al. 2017) |
|  |  | OsNAC5 | Rice | Overexpression | Rice | Enhanced | (Chung et al. 2018) |
|  |  | MfNACsa | Medicago | Overexpression/Mutation | Medicago | Enhanced/Reduced | (Duan et al. 2017) |
|  |  | TaNAC2a | Wheat | Overexpression | Tobacco | Enhanced | (Tang et al. 2012b) |
|  |  | TaNAC67 | Wheat | Overexpression | Arabidopsis | Enhanced | (Mao et al. 2014) |
|  |  | TaNAC47 | Wheat | Overexpression | Arabidopsis | Enhanced | (Zhang et al. 2015b) |
|  |  | CaNAC46 | Pepper | Overexpression | Arabidopsis | Enhanced | (Ma et al. 2021) |
|  |  | PbeNAC1 | Pyrus | Overexpression | Tobacco | Enhanced | (Jin et al. 2017) |
|  |  | MlNAC5 | Miscanthus | Overexpression | Arabidopsis | Enhanced | (Yang et al. 2015) |
|  |  | AaNAC1 | Artemisia annua | Overexpression | Artemisia annua | Enhanced | (Lv et al. 2016) |
|  |  | VvNAC08 | Grape | Overexpression | Arabidopsis | Enhanced | (Ju et al. 2020a) |
|  |  | StNAC053 | Potato | Overexpression | Arabidopsis | Enhanced | (Wang et al. 2021d) |
|  |  | FaNAC2 | Strawberry | Overexpression | Tobacco | Enhanced | (Liang et al. 2020) |
|  |  | LlNAC2 | Lily | Overexpression | Arabidopsis | Enhanced | (Yong et al. 2019b) |
|  |  | PeNAC034 | Poplar | Overexpression | Arabidopsis | Reduced | (Lu et al. 2018) |
|  |  | PeNAC045 | Poplar | Overexpression | Poplar | Reduced | (Lu et al. 2018) |
|  |  | CpNAC68 | Wintersweet | Overexpression | Arabidopsis | Enhanced | (Lin et al. 2021) |
|  | OsNAC3 | MusaSNAC1 | Banana | Overexpression | Banana | Enhanced | (Negi et al. 2018) |
|  |  | OsNAC9(SNAC1) | Rice | Overexpression | Rice, wheat, Cotton | Enhanced | (Redillas et al. 2012; Hu et al. 2006; Liu et al. 2014c; Saad et al. 2013) |
|  |  | ZmNAC55 | Maize | Overexpression | Arabidopsis | Enhanced | (Mao et al. 2016) |
|  |  | ZmSNAC1 | Maize | Overexpression | Arabidopsis | Enhanced | (Lu et al. 2012) |
|  |  | ZmNAC49 | Maize | Overexpression | Maize | Enhanced | (Xiang et al. 2021c) |
|  |  | ZmNAC080308 | Maize | Overexpression | Arabidopsis | Enhanced | (Wang et al. 2021c) |
|  |  | MlNAC12 | Miscanthus | Overexpression | Arabidopsis | Enhanced | (Yang et al. 2018a) |
|  |  | EcNAC67 | Finger millet | Overexpression | Rice | Enhanced | (Rahman et al. 2016) |
|  |  | TaNAC2D | Wheat | Overexpression | Arabidopsis | Enhanced or Reduced | (Huang and Wang 2016) |
|  |  | TaSNAC4-3A | Wheat | Overexpression | Arabidopsis | Enhanced | (Mei et al. 2021) |
|  |  | TaNAC2 | Wheat | Overexpression | Arabidopsis | Enhanced | (Mao et al. 2012) |
|  | NAC2 | NAC016 | Arabidopsis | Overexpression/Mutation | Arabidopsis | Reduced/Enhanced | (Sakuraba et al. 2015) |
|  |  | NTL4 | Arabidopsis | Overexpression/Mutation | Arabidopsis | Reduced/Enhanced | (Lee et al. 2012) |
|  |  | SlNAC7 | Tomato | Overexpression | Arabidopsis | Enhanced | (Wang et al. 2021b) |
|  |  | SlSRN1 | Tomato | Silence | Tomato | Enhanced | (Liu et al. 2014a) |
|  | ANACO11 | ANAC096 | Arabidopsis | Overexpression/mutation | Arabidopsis | Enhanced/Reduced | (Xu et al. 2013) |
|  |  | ANAC069 | Arabidopsis | Overexpression/Silence | Arabidopsis | Reduced/Enhanced | (He et al. 2017) |
|  |  | SlNAC6 | Tomato | Overexpression/Silence | Tomato | Enhanced/Reduced | (Jian et al. 2021) |
|  | TIP | NTL6 | Arabidopsis | Overexpression/Silence | Arabidopsis | Enhanced/Reduced | (Kim et al. 2012) |
|  |  | SlNAC1 | Suaeda liaotungensis | Overexpression | Arabidopsis | Enhanced | (Li et al. 2014c) |
|  | OsNAC8 | - | - | - | - | - |  |
|  | OsNAC7 | - | - | - | - | - |  |
|  | NAC1 | OMTN4 | Rice | Overexpression | Rice | Reduced | (Fang et al. 2014) |
|  |  | OMTN6 | Rice | Overexpression | Rice | Reduced | (Fang et al. 2014) |
|  |  | PheNAC3 | Moso bamboo | Overexpression | Arabidopsis | Enhanced | (Xie et al. 2020) |
|  |  | PeNAC070 | Poplar | Overexpression | Arabidopsis | Enhanced | (Lu et al. 2017) |
|  | NAM | OMTN2 | Rice | Overexpression | Rice | Reduced | (Fang et al. 2014) |
|  |  | OMTN3 | Rice | Overexpression | Rice | Reduced | (Fang et al. 2014) |
|  |  | OsNAC2 | Rice | Overexpression/Silence | Rice | Reduced/Enhanced | (Shen et al. 2017) |
|  |  | ONAC045 | Rice | Overexpression | Rice | Enhanced | (Zheng et al. 2009) |
|  |  | TaRNAC1 | Wheat | Overexpression | Wheat | Enhanced | (Chen et al. 2018) |
|  |  | RhNAC2 | Rose | Overexpression | Arabidopsis | Enhanced | (Dai et al. 2012) |
|  |  | SlNAC2 | Tomato | Overexpression | Arabidopsis, Tobacco | Enhanced | (Borgohain et al. 2019; van Beek et al. 2021) |
|  | ANAC001 | - | - | - | - | - |  |
|  | ONAC003 | OsNAC006 | Rice | Mutation | Rice | Reduced | (Wang et al. 2020a) |
|  |  | SNAC3 | Rice | Overexpression/Silence | Rice | Enhanced/Reduced | (Fang et al. 2015) |
|  |  | ZmNAC84 | Maize | Overexpression | Maize, Tobacco | Enhanced | (Zhu et al. 2016a; Han et al. 2021) |
|  | ONAC001 | - | - | - | - | - |  |
|  | ANAC063 | - | - | - | - | - |  |
|  | unclassified | MdNAC1 | Apple | Overexpression | Apple | Enhanced | (Jia et al. 2019) |
|  |  | SlNAC8 | Suaeda liaotungensis | Overexpression | Arabidopsis | Enhanced | (Wu et al. 2018) |
|  |  | GmNAC019 | Soybean | Overexpression | Arabidopsis | Enhanced | (Hoang et al. 2019) |
|  |  | PwNAC2 | Picea wilsonii | Overexpression | Arabidopsis | Enhanced | (Zhang et al. 2018b) |
|  |  | TsNAC1 | Thellungiella halophile | Overexpression | Thellungiella halophile | Enhanced | (Liu et al. 2019a) |
| **ERF** | A1 | MbDREB1 | Apple | Overexpression | Arabidopsis | Enhanced | (Yang et al. 2011) |
|  |  | AtDREB1A | Arabidopsis | Overexpression | Salvia miltiorrhiza, Arabidopsis，Rice | Enhanced | (Wei et al. 2016; Ravikumar et al. 2014; Kasuga et al. 1999) |
|  |  | AtDREB1C | Arabidopsis | Overexpression | Salvia miltiorrhiza | Enhanced | (Wei et al. 2017c) |
|  |  | DREB1D | Arabidopsis | Overexpression | Soybean | Enhanced | (Guttikonda et al. 2014) |
|  |  | FTL1/DDF1 | Arabidopsis | Overexpression | Arabidopsis | Enhanced | (Kang et al. 2011) |
|  |  | CBF4 | Arabidopsis | Overexpression | Arabidopsis | Enhanced | (Haake et al. 2002) |
|  |  | OsDREB1A | Rice | Overexpression | Arabidopsis, Rice | Enhanced | (Dubouzet et al. 2003) |
|  |  | ClCBF1 | Chrysanthemum | Overexpression | Chrysanthemum | Enhanced | (Gao et al. 2018b) |
|  |  | MrCBF2 | Muscadinia rotundifolia | Overexpression | Arabidopsis | Enhanced | (Wu et al. 2017) |
|  | A2 | AmDREB2C | Ammopiptanthus mongolicus | Overexpression | Arabidopsis | Enhanced | (Yin et al. 2018) |
|  |  | FaDREB2 | Festuca arundinacea Schreb | Overexpression | paper mulberry | Enhanced | (Li et al. 2011) |
|  |  | DREB2A | Arabidopsis | Overexpression | Arabidopsis | Enhanced | (Sakuma et al. 2006) |
|  |  | OsDREB2A | Rice | Overexpression | Rice | Enhanced | (Cui et al. 2011) |
|  |  | PeDREB2L | Poplar | Overexpression | Arabidopsis | Enhanced | (Chen et al. 2011) |
|  |  | VrDREB2A | Mung bean | Overexpression | Arabidopsis | Enhanced | (Chen et al. 2016) |
|  |  | VuDREB2A | Cowpea | Overexpression | Arabidopsis | Enhanced | (Sadhukhan et al. 2014) |
|  | A3 | ABI4 | Arabidopsis | Mutation | Arabidopsis | Reduced | (Khan et al. 2020) |
|  | A4 | TINY | Arabidopsis | Overexpression/Mutation | Arabidopsis | Enhanced/Reduced | (Xie et al. 2019) |
|  |  | HARDY | Arabidopsis | Overexpression | Arabidopsis,Rice,Trifolium alexandrinum L. | Enhanced | (Abogadallah et al. 2011; Karaba et al. 2007) |
|  |  | OsAP21 | Rice | Overexpression | Arabidopsis | Enhanced | (Jin et al. 2013) |
|  | A5 | NtERF172 | Tobacco | Overexpression/Silence | Tobacco | Enhanced/Reduced | (Zhao et al. 2020b) |
|  |  | GhDREB | Cotton | Overexpression | Wheat | Enhanced | (Gao et al. 2009) |
|  |  | GmDREB2 | Soybean | Overexpression | Arabidopsis | Enhanced | (Chen et al. 2007) |
|  |  | PeDREB2a | Poplar | Overexpression | Arabidopsis,Lotus corniculatus,cotton | Enhanced | (Li et al. 2016b; Zhou et al. 2012) |
|  |  | AtERF019 | Arabidopsis | Overexpression | Arabidopsis | Enhanced | (Scarpeci et al. 2017) |
|  |  | StDREB2 | Potato | Overexpression | Cotton | Enhanced | (El-Esawi and Alayafi 2019) |
|  | A6 | CmERF053 | Chrysanthemum | Overexpression | Arabidopsis | Enhanced | (Nie et al. 2018) |
|  |  | MdDREB76 | Apple | Overexpression | Tobacco | Enhanced | (Sharma et al. 2019) |
|  |  | AhDREB1 | Peanut | Overexpression | Arabidopsis | Enhanced | (Zhang et al. 2018a) |
|  |  | WXP1 | Medicago | Overexpression | Arabidopsis, Medicago | Enhanced | (Zhang et al. 2007; Zhang et al. 2005) |
|  |  | WXP2 | Medicago | Overexpression | Arabidopsis | Enhanced | (Zhang et al. 2007) |
|  |  | AhERF019 | Peanut | Overexpression | Arabidopsis | Enhanced | (Wan et al. 2014) |
|  |  | OsERF48 | Rice | Overexpression | Rice | Enhanced | (Jung et al. 2017) |
|  |  | AtERF53 | Arabidopsis | Overexpression | Arabidopsis | Enhanced | (Cheng et al. 2012) |
|  |  | RAP2.4 | Arabidopsis | Overexpression | Arabidopsis | Enhanced | (Lin et al. 2008) |
|  |  | TG | Arabidopsis | Overexpression | Arabidopsis | Enhanced | (Zhu et al. 2014a) |
|  | B1 | OsDRAP1 | Rice | Overexpression/Silence | Rice | Enhanced/Reduced | (Huang et al. 2018a) |
|  |  | AP37 | Rice | Overexpression | Rice | Enhanced | (Oh et al. 2009) |
|  |  | BrERF4 | Cabbage | Overexpression | Arabidopsis | Enhanced | (Seo et al. 2010) |
|  | B2 | IbRAP2-12 | Sweetpotato | Overexpression | Arabidopsis | Enhanced | (Li et al. 2019c) |
|  |  | OsEBP89 | Rice | Mutation | Rice | Enhanced | (Zhang et al. 2020c) |
|  |  | OsEREBP1 | Rice | Overexpression | Rice | Enhanced | (Jisha et al. 2015) |
|  |  | OsERF71 | Rice | Overexpression | Rice | Enhanced | (Lee et al. 2016) |
|  |  | CaPF1 | Pepper | Overexpression | Pinus strobus | Enhanced | (Tang et al. 2007) |
|  |  | ERF1-V | Haynaldia villosa | Overexpression | wheat | Enhanced | (Xing et al. 2017) |
|  |  | GmERF3 | Soybean | Overexpression | Tobacco | Enhanced | (Zhang et al. 2009) |
|  |  | HhERF2 | Halimodendron halodendron | Overexpression | Cotton | Enhanced | (Li et al. 2016b) |
|  | B3 | AtERF15 | Arabidopsis | Overexpression | Arabidopsis | Enhanced | (Lee et al. 2015b) |
|  |  | TaERF3 | Wheat | Overexpression/Silence | Wheat | Enhanced/Reduced | (Rong et al. 2014) |
|  |  | SlERF5 | Tomato | Overexpression | Tomato | Enhanced | (Pan et al. 2012) |
|  |  | CaAIEF1 | Pepper | Overexpression/Silence | Arabidopsis/pepper | Enhanced/Reduced | (Hong et al. 2017) |
|  |  | SpERF1 | Stipa purpurea | Overexpression | Arabidopsis | Enhanced | (Yang et al. 2016b) |
|  |  | TSRF1 | Tomato | Overexpression | Rice | Enhanced | (Quan et al. 2010) |
|  |  | OsERF83 | Rice | Overexpression | Rice | Enhanced | (Jung et al. 2021) |
|  |  | AP59 | Rice | Overexpression | Rice | Enhanced | (Oh et al. 2009) |
|  | B4 | ABR1 | Arabidopsis | Mutation | Arabidopsis | Reduced | (Pandey et al. 2005) |
|  |  | JcERF2 | Jatropha curcas L. | Overexpression | Tobacco | Enhanced | (Wang et al. 2015c) |
|  |  | OsERF101 | Rice | Overexpression | Rice | Enhanced | (Jin et al. 2018) |
|  |  | SlERF84 | Tomato | Overexpression | Arabidopsis | Enhanced | (Li et al. 2018b) |
|  | B5 | - | - | - | - | - |  |
|  | B6 | MdSHINE2 | Apple | Overexpression | Arabidopsis | Enhanced | (Zhang et al. 2019b) |
|  |  | HvSHN1 | Barley | Overexpression | Tobacco | Enhanced | (Djemal and Khoudi 2021) |
|  |  | TdSHN1 | Wheat | Overexpression | Tobacco | Enhanced | (Djemal and Khoudi 2019) |
|  |  | TaSHN1 | Wheat | Overexpression | Wheat | Enhanced | (Bi et al. 2018) |
|  |  | PeSHN1 | Poplar | Overexpression | Poplar | Enhanced | (Meng et al. 2019) |
|  |  | OsWR1 | Rice | Overexpression/Silence | Rice | Enhanced/Reduced | (Wang et al. 2012) |
|  |  | SHN1 | Arabidopsis | Overexpression | Arabidopsis | Enhanced | (Aharoni et al. 2004) |
|  | unclassified | OsERF115 | Rice | Overexpression | Rice | Enhanced | (Park et al. 2021) |
| **WRKY** | I | FvWRKY42 | Strawberry | Overexpression | Arabidopsis | Enhanced | (Wei et al. 2018) |
|  |  | CkWRKY33 | Caragana Korshinskii | Overexpression | Arabidopsis | Enhanced | (Li et al. 2021c) |
|  |  | TaWRKY44 | Wheat | Overexpression | Tobacco | Enhanced | (Wang et al. 2015d) |
|  |  | TaWRKY2 | Wheat | Overexpression | Wheat,Arabidopsis | Enhanced | (Gao et al. 2018a; Niu et al. 2012) |
|  |  | TaWRKY19 | Wheat | Overexpression | Arabidopsis | Enhanced | (Niu et al. 2012) |
|  |  | GhWRKY25 | Cotton | Overexpression | Tobacco | Reduced | (Liu et al. 2016b) |
|  |  | IbWRKY2 | Sweetpotato | Overexpression | Arabidopsis | Enhanced | (Zhu et al. 2020a) |
|  |  | MuWRKY3 | Horsegram | Overexpression | Groundnut | Enhanced | (Kiranmai et al. 2018) |
|  |  | OsWRKY30 | Rice | Overexpression | Rice | Enhanced | (Shen et al. 2012) |
|  |  | SpWRKY1 | Tomato | Overexpression | Tobacco | Enhanced | (Li et al. 2015b) |
|  |  | ZmWRKY65 | Maize | Overexpression | Arabidopsis | Enhanced | (Huo et al. 2021) |
|  |  | XsWRKY20 | Yellowhorn | Overexpression/Silence | Tobacco/yellowhorn | Enhanced/Reduced | (Xiong et al. 2020) |
|  |  | PoWRKY1 | Polygonatum odoratum | Overexpression | Arabidopsis | Enhanced | (Wei et al. 2021b) |
|  | IIa | TaWRKY33 | Wheat | Overexpression | Arabidopsis | Enhanced | (He et al. 2016) |
|  |  | TaWRKY93 | Wheat | Overexpression | Arabidopsis | Enhanced | (Qin et al. 2015) |
|  |  | GmWRKY27 | Soybean | Overexpression | Soybean | Enhanced | (Wang et al. 2015a) |
|  |  | LcWRKY5 | Sheepgrass | Overexpression | Arabidopsis | Enhanced | (Ma et al. 2014) |
|  |  | ZmWRKY40 | Maize | Overexpression | Arabidopsis | Enhanced | (Wang et al. 2018b) |
|  |  | VaWRKY14 | Grape | Overexpression | Arabidopsis | Enhanced | (Zhang et al. 2018c) |
|  | IIb | CmWRKY1 | Chrysanthemum | Overexpression | Chrysanthemum | Enhanced | (Fan et al. 2016) |
|  | IIc | GbWRKY1 | Cotton | Overexpression | Cotton, Arabidopsis | Reduced | (Luo et al. 2020) |
|  |  | GhWRKY68 | Cotton | Overexpression | Tobacco | Reduced | (Jia et al. 2015) |
|  |  | MeWRKY20 | Cassava | Silence | Cassava | Reduced | (Wei et al. 2020) |
|  |  | AtWRKY28 | Arabidopsis | Overexpression | Arabidopsis | Enhanced | (Babitha et al. 2013) |
|  |  | OsWRKY11 | Rice | Overexpression | Rice | Enhanced | (Lee et al. 2018; Wu et al. 2009a) |
|  |  | GmWRKY16 | Soybean | Overexpression | Arabidopsis | Enhanced | (Ma et al. 2018b) |
|  |  | GmWRKY54 | Soybean | Overexpression | Soybean, Arabidopsis | Enhanced | (Wei et al. 2019; Zhou et al. 2008) |
|  |  | VlWRKY3 | Grape | Overexpression | Arabidopsis | Enhanced | (Guo et al. 2018) |
|  |  | VvWRKY13 | Grape | Overexpression | Arabidopsis | Reduced | (Hou et al. 2020b) |
|  |  | MaWRKY80 | Banana | Overexpression | Arabidopsis | Enhanced | (Liu et al. 2020a) |
|  |  | TaWRKY10 | Wheat | Overexpression | Tobacco | Enhanced | (Wang et al. 2013) |
|  | IId | GhWRKY17 | Cotton | Overexpression | Tobacco | Reduced | (Yan et al. 2014) |
|  |  | GhWRKY21 | Cotton | Silence | Cotton | Enhanced | (Wang et al. 2020b) |
|  |  | MfWRKY17 | Myrothamnus Flabellifolia | Overexpression | Arabidopsis | Enhanced | (Huang et al. 2020) |
|  |  | GmWRKY13 | Soybean | Overexpression | Arabidopsis | Reduced | (Zhou et al. 2008) |
|  |  | HaWRKY76 | Sunflower | Overexpression | Arabidopsis | Enhanced | (Raineri et al. 2015a) |
|  |  | AtWRKY11 | Arabidopsis | Mutation | Arabidopsis | Reduced | (Ali et al. 2018) |
|  |  | AtWRKY17 | Arabidopsis | Mutation | Arabidopsis | Reduced | (Ali et al. 2018) |
|  |  | ThWRKY4 | Tamarix Hispida | Overexpression | Arabidopsis | Enhanced | (Zheng et al. 2013) |
|  |  | EjWRKY17 | Eriobotrya Japonica | Overexpression | Arabidopsis | Enhanced | (Wang et al. 2021a) |
|  | IIe | BdWRKY36 | Brachypodium Distachyon | Overexpression | Tobacco | Enhanced | (Sun et al. 2015) |
|  |  | GhWRKY91 | Cotton | Overexpression | Arabidopsis | Enhanced | (Gu et al. 2019) |
|  |  | CmWRKY10 | Chrysanthemum | Overexpression | Chrysanthemum | Enhanced | (Jaffar et al. 2016) |
|  |  | ZmWRKY106 | Maize | Overexpression | Arabidopsis | Enhanced | (Wang et al. 2018a) |
|  | III | AtWRKY63 | Arabidopsis | Mutation | Arabidopsis | Reduced | (Ren et al. 2010) |
|  |  | AtWRKY53 | Arabidopsis | Overexpression | Arabidopsis | Reduced | (Sun and Yu 2015) |
|  |  | AtWRKY30 | Arabidopsis | Overexpression | Wheat | Enhanced | (El-Esawi et al. 2019) |
|  |  | WRKY46 | Arabidopsis | Mutation | Arabidopsis | Enhanced | (Chen et al. 2017) |
|  |  | WRKY54 | Arabidopsis | Mutation | Arabidopsis | Enhanced | (Chen et al. 2017) |
|  |  | WRKY70 | Arabidopsis | Mutation | Arabidopsis | Enhanced | (Chen et al. 2017) |
|  |  | GhWRKY41 | Cotton | Overexpression | Tobacco | Enhanced | (Chu et al. 2015) |
|  |  | GhWRKY33 | Cotton | Overexpression | Arabidopsis | Reduced | (Wang et al. 2019a) |
|  |  | GhWRKY27a | Cotton | Overexpression/Silence | Tobacco/Cotton | Reduced/Enhanced | (Yan et al. 2015) |
|  |  | MfWRKY70 | Myrothamnus Flabellifolia | Overexpression | Arabidopsis | Enhanced | (Xiang et al. 2021b) |
|  |  | TaWRKY146 | Wheat | Overexpression | Arabidopsis | Reduced | (Ma et al. 2017) |
|  |  | TaWRKY1 | Wheat | Overexpression | Arabidopsis | Enhanced | (He et al. 2016) |
|  |  | TaWRKY46 | Wheat | Overexpression | Arabidopsis | Enhanced | (Li et al. 2020e) |
|  |  | GmWRKY12 | Soybean | Overexpression | Soybean | Enhanced | (Shi et al. 2018) |
|  |  | GsWRKY20 | soybean | Overexpression | Arabidopsis | Enhanced | (Luo et al. 2013) |
|  |  | FcWRKY70 | Fortunella Crassifolia | Overexpression/Silence | Tobacco, lemon /kumquat | Enhanced/Reduced | (Gong et al. 2015) |
|  |  | TcWRKY53 | Thlaspi Caerulescens | Overexpression | Tobacco | Reduced | (Wei et al. 2008) |
|  |  | OsWRKY47 | Rice | Overexpression/Mutation | Rice | Enhanced/Reduced | (Raineri et al. 2015b) |
|  |  | OsWRKY55 | Rice | Overexpression | Rice | Reduced | (Huang et al. 2021) |
|  |  | SbWRKY30 | Sorghum | Overexpression | Arabidopsis, Rice | Enhanced | (Yang et al. 2020c) |
|  |  | PbrWRKY53 | Pyrus | Overexpression/Silence | Tobacco/Pyrus | Enhanced | (Liu et al. 2019e) |
| **bZIP** | A | TaABL1 | Wheat | Overexpression | Wheat | Enhanced | (Xu et al. 2014) |
|  |  | TabZIP174 | Wheat | Overexpression | Arabidopsis | Enhanced | (Li et al. 2016d) |
|  |  | TabZIP60 | Wheat | Overexpression | Arabidopsis | Enhanced | (Zhang et al. 2015c) |
|  |  | Wabi5 | Wheat | Overexpression | Tobacco | Enhanced | (Kobayashi et al. 2008) |
|  |  | TaAREB3 | Wheat | Overexpression | Arabidopsis | Enhanced | (Wang et al. 2016d) |
|  |  | ABP9 | Maize | Overexpression | Cotton, Arabidopsis | Enhanced | (Wang et al. 2017a; Zhang et al. 2011) |
|  |  | ZmbZIP72 | Maize | Overexpression | Arabidopsis | Enhanced | (Ying et al. 2012) |
|  |  | ZmbZIP4 | Maize | Overexpression | Maize | Enhanced | (Ma et al. 2018a) |
|  |  | AREB1 | Arabidopsis | mutant | Arabidopsis | Reduced | (Yoshida et al. 2010) |
|  |  | AREB2 | Arabidopsis | mutant | Arabidopsis | Reduced | (Yoshida et al. 2010) |
|  |  | ABF1 | Arabidopsis | Mutation | Arabidopsis | Reduced | (Yoshida et al. 2015) |
|  |  | AtABF3 | Arabidopsis | Overexpression/Mutation | Medicago, Cotton/Arabidopsis | Enhanced/Reduced | (Yoshida et al. 2010; Kerr et al. 2018; Wang et al. 2016f) |
|  |  | AtABI5 | Arabidopsis | Overexpression | Cotton | Enhanced | (Mittal et al. 2014) |
|  |  | PtrAREB1-2 | Poplar | Silence | Poplar | Reduced | (Li et al. 2019b) |
|  |  | HvABI5 | Barley | mutant | Barley | Enhanced | (Collin et al. 2020) |
|  |  | BnaABF2 | Rapeseed | Overexpression | Arabidopsis | Enhanced | (Zhao et al. 2016a) |
|  |  | OsABF1 | Rice | Mutation | Rice | Reduced | (Amir Hossain et al. 2010) |
|  |  | OsbZIP23 | Rice | Overexpression/Mutation | Rice | Enhanced/Reduced | (Xiang et al. 2008) |
|  |  | OsABF2 | Rice | Mutation | Rice | Reduced | (Hossain et al. 2010) |
|  |  | OsbZIP46CA1 | Rice | Overexpression | Rice | Enhanced | (Tang et al. 2012a) |
|  |  | OsbZIP72 | Rice | Overexpression | Rice | Enhanced | (Lu et al. 2009) |
|  |  | OsbZIP42 | Rice | Overexpression | Rice | Enhanced | (Joo et al. 2019b) |
|  |  | OsbZIP62 | Rice | Overexpression/Mutation | Rice | Enhanced/Reduced | (Yang et al. 2019a) |
|  |  | CbABF1 | Cryophyte | Overexpression | Tobacco | Enhanced | (Yue et al. 2019) |
|  |  | ABF2D | Cotton | Overexpression | Cotton | Enhanced | (Kerr et al. 2018) |
|  |  | GhABF2 | Cotton | Overexpression | Arabidopsis/Cotton | Enhanced | (Liang et al. 2016) |
|  |  | VlbZIP30 | Grape | Overexpression | Arabidopsis | Enhanced | (Tu et al. 2018) |
|  |  | VvABF2 | Grape | Overexpression | Arabidopsis | Enhanced | (Liu et al. 2019b) |
|  |  | AhAREB1 | Peanut | Overexpression | Arabidopsis | Enhanced | (Li et al. 2013) |
|  |  | GmFDL19 | Soybean | Overexpression | Soybean | Enhanced | (Li et al. 2017c) |
|  |  | GmbZIP1 | Soybean | Overexpression | Wheat | Enhanced | (Gao et al. 2011) |
|  |  | PtrABF | Poncirus Trifoliata | Overexpression | Tobacco | Enhanced | (Huang et al. 2010) |
|  |  | CaATBZ1 | Pepper | Overexpression/Silence | Arabidopsis/Pepper | Reduced/Enhanced | (Joo et al. 2019a) |
|  |  | IbABF4 | Sweetpotato | Overexpression | Sweetpotato | Enhanced | (Wang et al. 2019b) |
|  |  | FtbZIP5 | Buckwheat | Overexpression | Arabidopsis | Enhanced | (Li et al. 2020b) |
|  |  | FtbZIP83 | Buckwheat | Overexpression | Arabidopsis | Enhanced | (Li et al. 2019a) |
|  |  | SlAREB1 | Tomato | Overexpression | Tomato | Enhanced | (Orellana et al. 2010) |
|  | B | - | - | - | - | - |  |
|  | C | OsbZIP52 | Rice | Overexpression | Rice | Reduced | (Liu et al. 2012) |
|  | D | AtTGA4 | Arabidopsis | Overexpression | Arabidopsis | Enhanced | (Zhong et al. 2015) |
|  |  | BnbZIP2 | Ramie | Overexpression | Arabidopsis | Reduced | (Huang et al. 2016a) |
|  |  | CaDILZ1 | Pepper | Overexpression/Silence | Arabidopsis/Pepper | Enhanced/Reduced | (Lim et al. 2018) |
|  |  | SlbZIP38 | Tomato | Overexpression | Tomato | Reduced | (Pan et al. 2017) |
|  | E | IbbZIP1 | Sweetpotato | Overexpression | Arabidopsis | Enhanced | (Kang et al. 2019) |
|  | F | GmbZIP19 | Soybean | Overexpression | Arabidopsis | Reduced | (He et al. 2020a) |
|  | G | AtGBF3 | Arabidopsis | Overexpression | Arabidopsis | Enhanced | (Ramegowda et al. 2017) |
|  |  | EcGBF3 | Finger millet | Overexpression | Arabidopsis | Enhanced | (Ramegowda et al. 2017) |
|  | H | - | - | - | - | - |  |
|  | I | - | - | - | - | - |  |
|  | S | AtbZIP1 | Arabidopsis | Overexpression/Mutation | Arabidopsis | Enhanced/Reduced | (Sun et al. 2012) |
|  |  | OsbZIP16 | Rice | Overexpression | Rice | Enhanced | (Chen et al. 2012) |
|  |  | OsbZIP71 | Rice | Overexpression | Rice | Enhanced | (Liu et al. 2014b) |
|  |  | SlbZIP1 | Tomato | Silence | Tomato | Reduced | (Zhu et al. 2018) |
|  |  | ThbZIP1 | Tamarix Hispida | Overexpression | Arabidopsis | Enhanced | (Ji et al. 2013) |
|  |  | CaBZ1 | Pepper | Overexpression | Potato | Enhanced | (Moon et al. 2015) |
|  |  | GmbZIP2 | Soybean | Overexpression | Arabidopsis | Enhanced | (Yang et al. 2020b) |
|  | M | - | - | - | - | - |  |
|  | K | VlbZIP36 | Grape | Overexpression | Arabidopsis | Enhanced | (Tu et al. 2016) |
|  |  | CAbZIP1 | Pepper | Overexpression | Arabidopsis | Enhanced | (Lee et al. 2006) |
|  |  | GmbZIP15 | Soybean | Overexpression | Soybean | Reduced | (Zhang et al. 2020b) |
|  |  | TabZIP | Wheat | Overexpression | Arabidopsis | Enhanced | (Agarwal et al. 2019) |
|  | J | AtbZIP62 | Arabidopsis | Mutation | Arabidopsis | Reduced | (Rolly et al. 2020) |
| **R2R3**  **-MYB** | 1 | CsMYB30 | Citrus Sinensis | Overexpression | Arabidopsis | Enhanced | (Wen et al. 2021) |
|  |  | AtMYB60 | Arabidopsis | Mutation | Arabidopsis | Enhanced | (Cominelli et al. 2005) |
|  |  | MYB96 | Arabidopsis | Overexpression/Mutation | Arabidopsis | Enhanced/Reduced | (Seo et al. 2009) |
|  |  | PtrMYB94 | Poplar | Overexpression | Poplar | Enhanced | (Fang et al. 2020) |
|  |  | TaMYB31-B | Wheat | Overexpression | Arabidopsis | Enhanced | (Zhao et al. 2018) |
|  | 2 | TaMYB33 | Wheat | Overexpression | Arabidopsis | Enhanced | (Qin et al. 2012) |
|  |  | MdSIMYB1 | Apple | Overexpression | Tobacco | Enhanced | (Wang et al. 2014b) |
|  |  | GmMYB14 | Soybean | Overexpression | Soybean | Enhanced | (Chen et al. 2021) |
|  |  | MYB15 | Arabidopsis | Overexpression | Arabidopsis | Enhanced | (Ding et al. 2009) |
|  | 3 | - | - | - | - | - |  |
|  | 4 | AcoMYB4 | Pineapple | Overexpression | Rice, Arabidopsis | Reduced | (Chen et al. 2020b) |
|  |  | AtMYB32 | Arabidopsis | Mutation | Arabidopsis | Enhanced | (Li et al. 2021b) |
|  |  | HvMYB1 | Barley | Overexpression | Barley | Enhanced | (Alexander et al. 2019) |
|  |  | GmMYB3a | Soybean | Overexpression | Peanut | Enhanced | (He et al. 2020b) |
|  |  | VcMYB4a | Blueberry | Overexpression | Blueberry | Reduced | (Zhang et al. 2020a) |
|  |  | TaMyb1D | Wheat | Overexpression | Tobacco | Enhanced | (Wei et al. 2017b) |
|  | 5 | TaMpc1-D4 | Wheat | Overexpression | Arabidopsis | Reduced | (Li et al. 2020d) |
|  | 6 | AmROSEA1 | Antirrhinum Majus | Overexpression | Rice | Enhanced | (Dou et al. 2016) |
|  | 7 | - | - | - | - | - |  |
|  | 8 | - | - | - | - | - |  |
|  | 9 | - | - | - | - | - |  |
|  | 10 | - | - | - | - | - |  |
|  | 11 | LlMYB3 | Lily | Overexpression | Arabidopsis | Enhanced | (Yong et al. 2019a) |
|  |  | GmMYBJ1 | Soybean | Overexpression | Arabidopsis | Enhanced | (Su et al. 2014) |
|  |  | MYB49 | Tomato | Overexpression | Tomato | Enhanced | (Cui et al. 2018) |
|  | 12 | - | - | - | - | - |  |
|  | 13 | PtoMYB170 | Poplar | Overexpression | Arabidopsis | Enhanced | (Xu et al. 2017) |
|  | 14 | GbMYB5 | Cotton | Overexpression/Silence | Tobacco/Cotton | Enhanced/Reduced | (Chen et al. 2015b) |
|  |  | OsMYB55 | Rice | Overexpression | Maize | Enhanced | (Casaretto et al. 2016) |
|  |  | MYB37 | Arabidopsis | Overexpression | Arabidopsis | Enhanced | (Yu et al. 2016c) |
|  | 15 | - | - | - | - | - |  |
|  | 16 | - | - | - | - | - |  |
|  | 17 | - | - | - | - | - |  |
|  | 18 | - | - | - | - | - |  |
|  | 19 | - | - | - | - | - |  |
|  | 20 | MsMYB2L | Medicago | Overexpression | Arabidopsis | Enhanced | (Song et al. 2019) |
|  |  | GmMYB84 | Soybean | Overexpression | Soybean | Enhanced | (Wang et al. 2017b) |
|  |  | IbMYB116 | Sweetpotato | Overexpression | Arabidopsis | Enhanced | (Zhou et al. 2019) |
|  |  | FtMYB9 | Buckwheat | Overexpression | Arabidopsis | Enhanced | (Gao et al. 2017) |
|  |  | PbrMYB21 | Pyrus | Overexpression/Silence | Tobacco/Pyrus | Enhanced/Reduced | (Li et al. 2017b) |
|  |  | TaPIMP1 | Wheat | Overexpression/Silence | Wheat | Enhanced/Reduced | (Zhang et al. 2012c) |
|  |  | RmMYB108 | Rose | Overexpression | Arabidopsis | Enhanced | (Dong et al. 2021) |
|  | 21 | TaSIM | Wheat | Overexpression | Arabidopsis | Enhanced | (Yu et al. 2019) |
|  |  | SiMYB56 | Foxtail Millet | Overexpression | Rice | Enhanced | (Xu et al. 2020a) |
|  | 22 | GmMYB81 | Soybean | Overexpression | Arabidopsis | Enhanced | (Bian et al. 2020) |
|  |  | CmMYB2 | Chrysanthemum | Overexpression | Arabidopsis | Enhanced | (Shan et al. 2012) |
|  |  | AtMYB44 | Arabidopsis | Overexpression/Mutation | Arabidopsis | Enhanced/Reduced | (Persak and Pitzschke 2014; Jung et al. 2008) |
|  |  | FtMYB13 | Buckwheat | Overexpression | Arabidopsis | Enhanced | (Huang et al. 2018b) |
|  |  | FtMYB10 | Buckwheat | Overexpression | Arabidopsis | Reduced | (Gao et al. 2016) |
|  |  | XsMYB44 | Yellowhorn | Silence | Yellowhorn | Reduced | (Li et al. 2021a) |
|  |  | SpMYB | Tomato | Overexpression | Tobacco | Enhanced | (Li et al. 2014a) |
|  |  | OsMYB102 | Rice | Overexpression | Arabidopsis | Reduced | (Piao et al. 2019) |
|  | 23 | OsMYB6 | Rice | Overexpression | Rice | Enhanced | (Tang et al. 2019) |
|  | 24 | TaMYBsm1-D | Wheat | Overexpression | Arabidopsis | Enhanced | (Li et al. 2016c) |
|  | 25 | - | - | - | - | - |  |
|  | unclassified | SiMYB75 | Sesame | Overexpression | Arabidopsis | Enhanced | (Dossa et al. 2020) |
|  |  | GaMYB85 | Cotton | Overexpression | Arabidopsis | Enhanced | (Butt et al. 2017) |
|  |  | NbPHAN | Tobacco | Silence | Tobacco | Reduced | (Huang et al. 2013) |
|  |  | OsMYB48-1 | Rice | Overexpression | Rice | Enhanced | (Xiong et al. 2014) |
|  |  | ScMYBAS1-3 | Sugarcane | Overexpression | Rice | Enhanced | (Favero Peixoto-Junior et al. 2018) |
|  |  | LpMYB1 | Lablab Purpureus | Overexpression | Arabidopsis | Enhanced | (Yao et al. 2016) |
|  |  | TaMYB30-B | Wheat | Overexpression | Arabidopsis | Enhanced | (Zhang et al. 2012a) |
|  |  | TaODORANT1 | Wheat | Overexpression | Tobacco | Enhanced | (Wei et al. 2017a) |
|  |  | MdoMYB121 | Apple | Overexpression | Tomato, Apple | Enhanced | (Cao et al. 2013) |
|  |  | MdMYB88 | Apple | Overexpression/Silence | Apple | Enhanced/Reduced | (Xie et al. 2021) |
|  |  | MdMYB124 | Apple | Overexpression/Silence | Apple | Enhanced/Reduced | (Xie et al. 2021) |
| **HD-ZIP** | I | TaHDZ5-6A | Wheat | Overexpression | Arabidopsis | Enhanced | (Li et al. 2020c) |
|  |  | TaHDZipI-5 | Wheat | Overexpression | Wheat | Enhanced | (Yang et al. 2018b) |
|  |  | CaHDZ12 | Chickpea | Overexpression | Tobacco | Enhanced | (Sen et al. 2017) |
|  |  | Hahb-4 | Sunflower | Overexpression | Arabidopsis | Enhanced | (Manavella et al. 2006) |
|  |  | HaHB11 | Sunflower | Overexpression | Arabidopsis | Enhanced | (Cabello et al. 2017) |
|  |  | HaHB1 | Sunflower | Overexpression | Arabidopsis | Enhanced | (Cabello and Chan 2012) |
|  |  | Oshox22 | Rice | Overexpression/Mutation | Rice | Reduced/Enhanced | (Zhang et al. 2012b) |
|  |  | AtHB13 | Arabidopsis | Overexpression | Arabidopsis | Enhanced | (Cabello and Chan 2012) |
|  |  | Phehdz1 | Phyllostachys Edulis | Overexpression | Rice | Enhanced | (Gao et al. 2021) |
|  |  | Zmhdz10 | Maize | Overexpression | Rice/Arabidopsis | Enhanced | (Zhao et al. 2014) |
|  |  | CaHB12 | Coffea Arabica | Overexpression | Arabidopsis | Enhanced | (Basso et al. 2021) |
|  | II | EcHB1 | Ucalyptus Camaldulensis | Overexpression | Eucalyptus | Enhanced | (Sasaki et al. 2019) |
|  |  | ABIG1 | Arabidopsis | Mutation | Arabidopsis | Enhanced | (Liu et al. 2016a) |
|  |  | HAT1 | Arabidopsis | Overexpression/Mutation | Arabidopsis | Reduced/Enhanced | (Tan et al. 2018) |
|  |  | HAT3 | Arabidopsis | Mutation | Arabidopsis | Enhanced | (Tan et al. 2018) |
|  | III | - | - | - | - | - |  |
|  | IV | EDT1/HDG11 | Arabidopsis | Overexpression | Arabidopsis, Rice, Cotton, Tobacco, Poplar, tall fescue, Chinese kale | Enhanced | (Yu et al. 2016a; Zhu et al. 2016b; Yu et al. 2008; Yu et al. 2013; Cao et al. 2009) |
|  |  | OsTF1L | Rice | Overexpression | Rice | Enhanced | (Bang et al. 2019) |
|  |  | ROC4 | Rice | Overexpression/Mutation | Rice | Enhanced/Reduced | (Wang et al. 2018d) |
| **ZnF** | A1 | - | - | - | - | - |  |
|  | A2 | - | - | - | - | - |  |
|  | A3 | - | - | - | - | - |  |
|  | A4 | - | - | - | - | - |  |
|  | B | - | - | - | - | - |  |
|  | C1-1i | DST | Rice | Overexpression/Silence | Rice | Reduced/Enhanced | (Cui et al. 2015; Huang et al. 2009b) |
|  |  | GmZFP3 | Soybean | Overexpression | Arabidopsis | Reduced | (Zhang et al. 2016) |
|  | C1-Sa | GsZFP1 | Soybean | Overexpression | Arabidopsis, Medicago | Enhanced | (Luo et al. 2012; Tang et al. 2013) |
|  | C1-Q | - | - | - | - | - |  |
|  | C1 cons. | - | - | - | - | - |  |
|  | C1-unique | - | - | - | - | - |  |
|  | C1-2i | ZAT18 | Arabidopsis | Overexpression/Mutation | Arabidopsis | Enhanced/Reduced | (Yin et al. 2017) |
|  |  | STZ | Arabidopsis | Overexpression | Arabidopsis | Enhanced | (Sakamoto et al. 2004) |
|  |  | TaZFP1B | Wheat | Overexpression/Silence | Wheat | Enhanced/Reduced | (Cheuk et al. 2020) |
|  |  | ZFP252 | Rice | Overexpression | Rice | Enhanced | (Xu et al. 2008) |
|  |  | ZFP245 | Rice | Overexpression | Rice | Enhanced | (Huang et al. 2009a) |
|  |  | ZFP182 | Rice | Overexpression | Rice | Enhanced | (Huang et al. 2012) |
|  |  | OsDRZ1 | Rice | Overexpression/Silence | Rice | Enhanced/Reduced | (Yuan et al. 2018) |
|  |  | IbZFP1 | Sweetpotato | Overexpression | Arabidopsis | Enhanced | (Wang et al. 2016a) |
|  |  | DgZFP3 | Chrysanthemum | Overexpression | Tobacco | Enhanced | (Liu et al. 2013a) |
|  |  | CgZFP1 | Chrysanthemum | Overexpression | Arabidopsis | Enhanced | (Gao et al. 2012) |
|  |  | ZPT2-3 | Petunia | Overexpression | Petunia | Enhanced | (Sugano et al. 2003) |
|  | C1C2 mixed | - | - | - | - | - |  |
|  | C1-3i | GmZAT4 | Soybean | Overexpression | Arabidopsis | Enhanced | (Sun et al. 2019) |
|  | C1-4i | - | - | - | - | - |  |
|  | C1-5i | - | - | - | - | - |  |
|  | C2-1i | - | - | - | - | - |  |
|  | C2-B | - | - | - | - | - |  |
|  | C2-Sb | - | - | - | - | - |  |
|  | C2 pairs | - | - | - | - | - |  |
|  | C2-unique | - | - | - | - | - |  |
|  | C2 cons. | - | - | - | - | - |  |
|  | C3 | - | - | - | - | - |  |
|  | C3H | OsC3H47 | Rice | Overexpression | Rice | Enhanced | (Wang et al. 2015b) |
|  |  | OsTZF5 | Rice | Overexpression | Rice | Enhanced | (Selvaraj et al. 2020) |
|  |  | OsTZF1 | Rice | Overexpression/Silence | Rice | Enhanced/Reduced | (Jan et al. 2013) |
|  |  | OsC3H10 | Rice | Overexpression | Rice | Enhanced | (Seong et al. 2020) |
|  |  | IbC3H18 | Sweetpotato | Overexpression/Silence | Sweetpotato | Enhanced/Reduced | (Zhang et al. 2019a) |
|  |  | PdC3H17 | Poplar | Overexpression | Poplar | Enhanced | (Zhuang et al. 2019) |
|  |  | PeC3H74 | Moso Bamboo | Overexpression | Arabidopsis | Enhanced | (Chen et al. 2020a) |
|  | Di-19 | Di19 | Arabidopsis | Overexpression | Arabidopsis | Enhanced | (Liu et al. 2013b) |
|  |  | Di19-3 | Arabidopsis | Mutation/Overexpression | Arabidopsis | Enhanced/Reduced | (Qin et al. 2014) |
|  |  | OsDi19-4 | Rice | Overexpression | Rice | Enhanced | (Wang et al. 2014a) |
|  | BBX | MdBBX10 | Apple | Overexpression | Arabidopsis | Enhanced | (Liu et al. 2019d) |
|  |  | CmBBX19 | Chrysanthemum | Overexpression/Silence | Chrysanthemum | Reduced/Enhanced | (Xu et al. 2020b) |
|  | others | VOZ1 | Arabidopsis | Mutation | Arabidopsis | Enhanced | (Nakai et al. 2013) |
|  |  | VOZ2 | Arabidopsis | Mutation | Arabidopsis | Enhanced | (Nakai et al. 2013) |
|  |  | GmVOZ1G | Soybean | Overexpression/Silence | Soybean | Enhanced/Reduced | (Li et al. 2020a) |
|  |  | MdDof54 | Apple | Overexpression/Silence | Apple | Enhanced/Reduced | (Chen et al. 2020c) |
| **bHLH** | 1 | AabHLH35 | Anthurium Andraeanum | Overexpression | Arabidopsis | Enhanced | (Jiang et al. 2019) |
|  |  | ZjICE2 | Zoysia Japonica | Overexpression | Arabidopsis | Enhanced | (Zuo et al. 2020) |
|  |  | PebHLH35 | Poplar | Overexpression | Arabidopsis | Enhanced | (Dong et al. 2014) |
|  |  | FtbHLH3 | Buckwheat | Overexpression | Arabidopsis | Enhanced | (Yao et al. 2017) |
|  |  | AtICE1 | Arabidopsis | Overexpression | Rice | Enhanced | (Verma et al. 2020) |
|  | 2 | AtAIB(AtbHLH17) | Arabidopsis | Overexpression | Arabidopsis | Enhanced | (Babitha et al. 2013) |
|  | 3 | MdSAT1 | Apple | Overexpression | Arabidopsis, Apple | Enhanced | (Yang et al. 2021) |
|  | 4 | EcbHLH57 | Finger Millet | Overexpression | Tobacco | Enhanced | (Babitha et al. 2015) |
|  | 5 | AmDEL | Antirrhinum Majus | Overexpression | Arabidopsis, Tobacco | Enhanced | (Wang et al. 2016b) |
|  |  | leaf color (Lc) | Maize | Overexpression | Ma Bamboo | Enhanced | (Xiang et al. 2021a) |
|  | 7 | ZmbHLH124 | Maize | Overexpression | Maize, Rice | Enhanced | (Wei et al. 2021a) |
|  | 12 | MfbHLH38 | Myrothamnus Flabellifolia | Overexpression | Arabidopsis | Enhanced | (Qiu et al. 2020) |
|  | 15 | AtbHLH112 | Arabidopsis | Overexpression/Mutation | Arabidopsis | Enhanced/Reduced | (Liu et al. 2015) |
|  |  | AtbHLH68 | Arabidopsis | Overexpression | Arabidopsis | Enhanced | (Le Hir et al. 2017) |
|  | 18 | IBH1 | Arabidopsis | Overexpression | Arabidopsis | Enhanced | (Moreno et al. 2018) |
|  | 24 | ZmPIF3 | Maize | Overexpression | Rice | Enhanced | (Gao et al. 2015; Gao et al. 2018c) |
|  |  | TabHLH39 | Wheat | Overexpression | Arabidopsis | Enhanced | (Zhai et al. 2016) |
|  | 25 | BEE1/2/3 | Arabidopsis | Mutation | Arabidopsis | Enhanced | (Moreno et al. 2018) |
|  |  | SlbHLH22 | Tomato | Overexpression | Tomato | Enhanced | (Waseem et al. 2019) |
|  |  | TabHLH49 | Wheat | Overexpression/Silence | Wheat | Enhanced/Reduced | (Liu et al. 2020b) |
|  | 26 | ZmPTF1 | Maize | Overexpression/Mutation | Maize | Enhanced/Reduced | (Li et al. 2019d) |
|  |  | TabHLH1 | Wheat | Overexpression | Tobacco | Enhanced | (Yang et al. 2016a) |
|  | 27 | bHLH122 | Arabidopsis | Overexpression/Mutation | Arabidopsis | Enhanced/Reduced | (Liu et al. 2014d) |
|  |  | MdbHLH130 | Apple | Overexpression | Tobacco, Apple | Enhanced/Reduced | (Zhao et al. 2020a) |
| **ASR** | | SiASR1 | Foxtail Millet | Overexpression | Tobacco | Enhanced | (Feng et al. 2016) |
|  |  | SiASR4 | Foxtail Millet | Overexpression | Arabidopsis, Foxtail Millet | Enhanced | (Li et al. 2016a) |
|  |  | OsASR1 | Rice | Overexpression | Rice | Enhanced | (Park et al. 2019) |
|  |  | OsASR5 | Rice | Overexpression | Rice/Arabidopsis | Enhanced | (Li et al. 2017a) |
|  |  | ZmASR3 | Maize | Overexpression | Arabidopsis | Enhanced | (Liang et al. 2019) |
|  |  | MpAsr | Plantain | Overexpression | Arabidopsis | Enhanced | (Liu et al. 2010) |
|  |  | PgASR3 | Pearl Millet | Overexpression | Arabidopsis | Enhanced | (Meena et al. 2020) |
|  |  | HvASR5 | Barley | Overexpression | Rice, Arabidopsis | Enhanced | (Perez-Diaz et al. 2019) |
|  |  | SbASR-1 | Salicornia Brachiata | Overexpression | Groundnut | Enhanced | (Tiwari et al. 2015) |
|  |  | BdASR1 | Brachypodium Distachyon | Overexpression | Tobacco | Enhanced | (Wang et al. 2016e) |
|  |  | BdASR4 | Brachypodium Distachyon | Overexpression | Brachypodium Distachyon | Enhanced | (Yoon et al. 2019) |
|  |  | PheASR2 | Moso Bamboo | Overexpression | Rice | Enhanced | (Wu et al. 2020) |
|  |  | LLA23 | Lily | Overexpression | Arabidopsis | Enhanced | (Yang et al. 2005) |
|  |  | TtASR | Tetragonia Tetragonoides | Overexpression | Arabidopsis | Enhanced | (Ye et al. 2019) |
|  |  | TaASR2D | Wheat | Overexpression | Brachypodium Distachyon | Enhanced | (Yoon et al. 2021) |
|  |  | TaASR1 | Wheat | Overexpression | Tobacco | Enhanced | (Hu et al. 2013) |
|  |  | MaASR | Banana | Overexpression | Arabidopsis | Enhanced | (Zhang et al. 2015a) |
| **NF-Y** | | NFYA5 | Arabidopsis | Overexpression/Mutation | Arabidopsis | Enhanced/Reduced | (Li et al. 2008) |
|  |  | ZmNF-YA3 | Maize | Mutation | Maize | Reduced | (Su et al. 2018) |
|  |  | ZmNF-YB2 | Maize | Overexpression | Maize | Enhanced | (Nelson et al. 2007) |
|  |  | CsNF-YA5 | Sweet Orange | Overexpression | Tobacco | Enhanced | (Pereira et al. 2018) |
|  |  | GmNFYA3 | Soybean | Overexpression | Arabidopsis | Enhanced | (Ni et al. 2013) |
|  |  | GmNFYA5 | Soybean | Overexpression | Arabidopsis | Enhanced | (Ma et al. 2020) |
|  |  | OsNF-YA7 | Rice | Overexpression | Rice | Enhanced | (Lee et al. 2015a) |
|  |  | PwNF-YB3 | Picea Wilsonii | Overexpression | Arabidopsis | Enhanced | (Zhang et al. 2015d) |
|  |  | Cdt-NF-YC1 | Bermudagrass | Overexpression | Rice | Enhanced | (Chen et al. 2015a) |
|  |  | PdNF-YB7 | Poplar | Overexpression | Arabidopsis | Enhanced | (Han et al. 2013) |
|  |  | PdNF-YB21 | Poplar | Overexpression/Mutation | Poplar | Enhanced/Reduced | (Zhou et al. 2020) |
|  |  | TaNF-YA10-1 | Wheat | Overexpression | Arabidopsis | Enhanced | (Ma et al. 2015) |
| **HSF** | | TaHsfA6f | Wheat | Overexpresion | Arabidopsis | Enhanced | (Bi et al. 2020) |
|  |  | OsHSFA3 | Rice | Overexpresion | Arabidopsis | Enhanced | (Zhu et al. 2020b) |
|  |  | MdHSFA8a | Apple | Overexpresion/Silence | Apple | Enhanced/Reduced | (Wang et al. 2020d) |
|  |  | GmHsf-34 | Soybean | Overexpresion | Arabidopsis | Enhanced | (Li et al. 2014b) |
|  |  | ZmHsf06 | Maize | Overexpresion | Arabidopsis | Enhanced | (Li et al. 2015a) |
|  |  | BhHsf1 | Boea hygrometrica | Overexpresion | Arabidopsis,Tobacco | Enhanced | (Zhu et al. 2009) |
|  |  | HSFA6a/HSFA6b | Arabidopsis | Mutation | Arabidopsis,Tobacco | Enhanced | (Wenjing et al. 2020) |
|  |  | HSFA1b | Arabidopsis | Overexpresion | Arabidopsis | Enhanced | (Bechtold et al. 2013) |
| **Others** | | BrLAS | Pepper | Overexpresion | Arabidopsis | Enhanced | (Li et al. 2018a) |
|  |  | OsGRAS23 | Rice | Overexpresion | Rice | Enhanced | (Xu et al. 2015) |
|  |  | OsWOX13 | Rice | Overexpresion | Rice | Enhanced | (Minh-Thu et al. 2018) |
|  |  | PagKNAT2/6b | Poplar | Overexpresion | Poplar | Enhanced | (Song et al. 2021) |
|  |  | TaBZR2 | Wheat | Overexpresion/Silence | Wheat | Enhanced/Reduced | (Cui et al. 2019) |
|  |  | GTL1 | Arabidopsis | Mutation | Arabidopsis | Reduced | (Yoo et al. 2010) |

**References**

Abogadallah GM, Nada RM, Malinowski R, Quick P (2011) Overexpression of HARDY, an AP2/ERF gene from Arabidopsis, improves drought and salt tolerance by reducing transpiration and sodium uptake in transgenic Trifolium alexandrinum L. Planta 233 (6):1265-1276. doi:10.1007/s00425-011-1382-3

Agarwal P, Baranwal VK, Khurana P (2019) Genome-wide Analysis of bZIP Transcription Factors in wheat and Functional Characterization of a TabZIP under Abiotic Stress. Sci Rep 9 (1):4608. doi:10.1038/s41598-019-40659-7

Aharoni A, Dixit S, Jetter R, Thoenes E, van Arkel G, Pereira A (2004) The SHINE clade of AP2 domain transcription factors activates wax biosynthesis, alters cuticle properties, and confers drought tolerance when overexpressed in Arabidopsis. Plant Cell 16 (9):2463-2480. doi:10.1105/tpc.104.022897

Alexander RD, Wendelboe-Nelson C, Morris PC (2019) The barley transcription factor HvMYB1 is a positive regulator of drought tolerance. Plant Physiol Biochem 142:246-253. doi:10.1016/j.plaphy.2019.07.014

Ali MA, Azeem F, Nawaz MA, Acet T, Abbas A, Imran QM, Shah KH, Rehman HM, Chung G, Yang SH, Bohlmann H (2018) Transcription factors WRKY11 and WRKY17 are involved in abiotic stress responses in Arabidopsis. J Plant Physiol 226:12-21. doi:10.1016/j.jplph.2018.04.007

Amir Hossain M, Lee Y, Cho JI, Ahn CH, Lee SK, Jeon JS, Kang H, Lee CH, An G, Park PB (2010) The bZIP transcription factor OsABF1 is an ABA responsive element binding factor that enhances abiotic stress signaling in rice. Plant Mol Biol 72 (4-5):557-566. doi:10.1007/s11103-009-9592-9

Babitha KC, Ramu SV, Pruthvi V, Mahesh P, Nataraja KN, Udayakumar M (2013) Co-expression of AtbHLH17 and AtWRKY28 confers resistance to abiotic stress in Arabidopsis. Transgenic Res 22 (2):327-341. doi:10.1007/s11248-012-9645-8

Babitha KC, Vemanna RS, Nataraja KN, Udayakumar M (2015) Overexpression of EcbHLH57 Transcription Factor from Eleusine coracana L. in Tobacco Confers Tolerance to Salt, Oxidative and Drought Stress. PLoS One 10 (9):e0137098. doi:10.1371/journal.pone.0137098

Bang SW, Lee DK, Jung H, Chung PJ, Kim YS, Choi YD, Suh JW, Kim JK (2019) Overexpression of OsTF1L, a rice HD-Zip transcription factor, promotes lignin biosynthesis and stomatal closure that improves drought tolerance. Plant Biotechnol J 17 (1):118-131. doi:10.1111/pbi.12951

Basso MF, Costa JA, Ribeiro TP, Arraes FBM, Lourenco-Tessutti IT, Macedo AF, Neves MRD, Nardeli SM, Arge LW, Perez CEA, Silva PLR, de Macedo LLP, Lisei-de-Sa ME, Santos Amorim RM, Pinto ERC, Silva MCM, Morgante CV, Floh EIS, Alves-Ferreira M, Grossi-de-Sa MF (2021) Overexpression of the CaHB12 transcription factor in cotton (Gossypium hirsutum) improves drought tolerance. Plant Physiol Biochem 165:80-93. doi:10.1016/j.plaphy.2021.05.009

Bechtold U, Albihlal WS, Lawson T, Fryer MJ, Sparrow PA, Richard F, Persad R, Bowden L, Hickman R, Martin C, Beynon JL, Buchanan-Wollaston V, Baker NR, Morison JI, Schoffl F, Ott S, Mullineaux PM (2013) Arabidopsis HEAT SHOCK TRANSCRIPTION FACTORA1b overexpression enhances water productivity, resistance to drought, and infection. J Exp Bot 64 (11):3467-3481. doi:10.1093/jxb/ert185

Bi H, Shi J, Kovalchuk N, Luang S, Bazanova N, Chirkova L, Zhang D, Shavrukov Y, Stepanenko A, Tricker P, Langridge P, Hrmova M, Lopato S, Borisjuk N (2018) Overexpression of the TaSHN1 transcription factor in bread wheat leads to leaf surface modifications, improved drought tolerance, and no yield penalty under controlled growth conditions. Plant Cell Environ 41 (11):2549-2566. doi:10.1111/pce.13339

Bi H, Zhao Y, Li H, Liu W (2020) Wheat Heat Shock Factor TaHsfA6f Increases ABA Levels and Enhances Tolerance to Multiple Abiotic Stresses in Transgenic Plants. Int J Mol Sci 21 (9). doi:10.3390/ijms21093121

Bian S, Jin D, Sun G, Shan B, Zhou H, Wang J, Zhai L, Li X (2020) Characterization of the soybean R2R3-MYB transcription factor GmMYB81 and its functional roles under abiotic stresses. Gene 753:144803. doi:10.1016/j.gene.2020.144803

Borgohain P, Saha B, Agrahari R, Chowardhara B, Sahoo S, van der Vyver C, Panda SK (2019) SlNAC2 overexpression in Arabidopsis results in enhanced abiotic stress tolerance with alteration in glutathione metabolism. Protoplasma 256 (4):1065-1077. doi:10.1007/s00709-019-01368-0

Butt HI, Yang Z, Gong Q, Chen E, Wang X, Zhao G, Ge X, Zhang X, Li F (2017) GaMYB85, an R2R3 MYB gene, in transgenic Arabidopsis plays an important role in drought tolerance. BMC Plant Biol 17 (1):142. doi:10.1186/s12870-017-1078-3

Cabello JV, Chan RL (2012) The homologous homeodomain-leucine zipper transcription factors HaHB1 and AtHB13 confer tolerance to drought and salinity stresses via the induction of proteins that stabilize membranes. Plant Biotechnol J 10 (7):815-825. doi:10.1111/j.1467-7652.2012.00701.x

Cabello JV, Giacomelli JI, Gomez MC, Chan RL (2017) The sunflower transcription factor HaHB11 confers tolerance to water deficit and salinity to transgenic Arabidopsis and alfalfa plants. J Biotechnol 257:35-46. doi:10.1016/j.jbiotec.2016.11.017

Cao H, Wang L, Nawaz MA, Niu M, Sun J, Xie J, Kong Q, Huang Y, Cheng F, Bie Z (2017) Ectopic Expression of Pumpkin NAC Transcription Factor CmNAC1 Improves Multiple Abiotic Stress Tolerance in Arabidopsis. Front Plant Sci 8:2052. doi:10.3389/fpls.2017.02052

Cao YJ, Wei Q, Liao Y, Song HL, Li X, Xiang CB, Kuai BK (2009) Ectopic overexpression of AtHDG11 in tall fescue resulted in enhanced tolerance to drought and salt stress. Plant Cell Rep 28 (4):579-588. doi:10.1007/s00299-008-0659-x

Cao ZH, Zhang SZ, Wang RK, Zhang RF, Hao YJ (2013) Genome wide analysis of the apple MYB transcription factor family allows the identification of MdoMYB121 gene confering abiotic stress tolerance in plants. PLoS One 8 (7):e69955. doi:10.1371/journal.pone.0069955

Casaretto JA, El-Kereamy A, Zeng B, Stiegelmeyer SM, Chen X, Bi YM, Rothstein SJ (2016) Expression of OsMYB55 in maize activates stress-responsive genes and enhances heat and drought tolerance. BMC Genomics 17:312. doi:10.1186/s12864-016-2659-5

Chen D, Chai S, McIntyre CL, Xue GP (2018) Overexpression of a predominantly root-expressed NAC transcription factor in wheat roots enhances root length, biomass and drought tolerance. Plant Cell Rep 37 (2):225-237. doi:10.1007/s00299-017-2224-y

Chen F, Liu HL, Wang K, Gao YM, Wu M, Xiang Y (2020a) Identification of CCCH Zinc Finger Proteins Family in Moso Bamboo (Phyllostachys edulis), and PeC3H74 Confers Drought Tolerance to Transgenic Plants. Front Plant Sci 11:579255. doi:10.3389/fpls.2020.579255

Chen H, Chen W, Zhou J, He H, Chen L, Chen H, Deng XW (2012) Basic leucine zipper transcription factor OsbZIP16 positively regulates drought resistance in rice. Plant Sci 193-194:8-17. doi:10.1016/j.plantsci.2012.05.003

Chen H, Lai L, Li L, Liu L, Jakada BH, Huang Y, He Q, Chai M, Niu X, Qin Y (2020b) AcoMYB4, an Ananas comosus L. MYB Transcription Factor, Functions in Osmotic Stress through Negative Regulation of ABA Signaling. Int J Mol Sci 21 (16). doi:10.3390/ijms21165727

Chen H, Liu L, Wang L, Wang S, Cheng X (2016) VrDREB2A, a DREB-binding transcription factor from Vigna radiata, increased drought and high-salt tolerance in transgenic Arabidopsis thaliana. J Plant Res 129 (2):263-273. doi:10.1007/s10265-015-0773-0

Chen J, Nolan TM, Ye H, Zhang M, Tong H, Xin P, Chu J, Chu C, Li Z, Yin Y (2017) Arabidopsis WRKY46, WRKY54, and WRKY70 Transcription Factors Are Involved in Brassinosteroid-Regulated Plant Growth and Drought Responses. Plant Cell 29 (6):1425-1439. doi:10.1105/tpc.17.00364

Chen J, Xia X, Yin W (2011) A poplar DRE-binding protein gene, PeDREB2L, is involved in regulation of defense response against abiotic stress. Gene 483 (1-2):36-42. doi:10.1016/j.gene.2011.05.010

Chen L, Yang H, Fang Y, Guo W, Chen H, Zhang X, Dai W, Chen S, Hao Q, Yuan S, Zhang C, Huang Y, Shan Z, Yang Z, Qiu D, Liu X, Tran LP, Zhou X, Cao D (2021) Overexpression of GmMYB14 improves high-density yield and drought tolerance of soybean through regulating plant architecture mediated by the brassinosteroid pathway. Plant Biotechnol J 19 (4):702-716. doi:10.1111/pbi.13496

Chen M, Wang QY, Cheng XG, Xu ZS, Li LC, Ye XG, Xia LQ, Ma YZ (2007) GmDREB2, a soybean DRE-binding transcription factor, conferred drought and high-salt tolerance in transgenic plants. Biochem Biophys Res Commun 353 (2):299-305. doi:10.1016/j.bbrc.2006.12.027

Chen M, Zhao Y, Zhuo C, Lu S, Guo Z (2015a) Overexpression of a NF-YC transcription factor from bermudagrass confers tolerance to drought and salinity in transgenic rice. Plant Biotechnol J 13 (4):482-491. doi:10.1111/pbi.12270

Chen P, Yan M, Li L, He J, Zhou S, Li Z, Niu C, Bao C, Zhi F, Ma F, Guan Q (2020c) The apple DNA-binding one zinc-finger protein MdDof54 promotes drought resistance. Hortic Res 7 (1):195. doi:10.1038/s41438-020-00419-5

Chen T, Li W, Hu X, Guo J, Liu A, Zhang B (2015b) A Cotton MYB Transcription Factor, GbMYB5, is Positively Involved in Plant Adaptive Response to Drought Stress. Plant Cell Physiol 56 (5):917-929. doi:10.1093/pcp/pcv019

Chen X, Wang Y, Lv B, Li J, Luo L, Lu S, Zhang X, Ma H, Ming F (2014) The NAC family transcription factor OsNAP confers abiotic stress response through the ABA pathway. Plant Cell Physiol 55 (3):604-619. doi:10.1093/pcp/pct204

Cheng MC, Hsieh EJ, Chen JH, Chen HY, Lin TP (2012) Arabidopsis RGLG2, functioning as a RING E3 ligase, interacts with AtERF53 and negatively regulates the plant drought stress response. Plant Physiol 158 (1):363-375. doi:10.1104/pp.111.189738

Cheuk A, Ouellet F, Houde M (2020) The barley stripe mosaic virus expression system reveals the wheat C2H2 zinc finger protein TaZFP1B as a key regulator of drought tolerance. BMC Plant Biol 20 (1):144. doi:10.1186/s12870-020-02355-x

Chu X, Wang C, Chen X, Lu W, Li H, Wang X, Hao L, Guo X (2015) The Cotton WRKY Gene GhWRKY41 Positively Regulates Salt and Drought Stress Tolerance in Transgenic Nicotiana benthamiana. PLoS One 10 (11):e0143022. doi:10.1371/journal.pone.0143022

Chung PJ, Jung H, Choi YD, Kim JK (2018) Genome-wide analyses of direct target genes of four rice NAC-domain transcription factors involved in drought tolerance. BMC Genomics 19 (1):40. doi:10.1186/s12864-017-4367-1

Collin A, Daszkowska-Golec A, Kurowska M, Szarejko I (2020) Barley ABI5 (Abscisic Acid INSENSITIVE 5) Is Involved in Abscisic Acid-Dependent Drought Response. Front Plant Sci 11:1138. doi:10.3389/fpls.2020.01138

Cominelli E, Galbiati M, Vavasseur A, Conti L, Sala T, Vuylsteke M, Leonhardt N, Dellaporta SL, Tonelli C (2005) A guard-cell-specific MYB transcription factor regulates stomatal movements and plant drought tolerance. Curr Biol 15 (13):1196-1200. doi:10.1016/j.cub.2005.05.048

Cui J, Jiang N, Zhou X, Hou X, Yang G, Meng J, Luan Y (2018) Tomato MYB49 enhances resistance to Phytophthora infestans and tolerance to water deficit and salt stress. Planta 248 (6):1487-1503. doi:10.1007/s00425-018-2987-6

Cui LG, Shan JX, Shi M, Gao JP, Lin HX (2015) DCA1 Acts as a Transcriptional Co-activator of DST and Contributes to Drought and Salt Tolerance in Rice. PLoS Genet 11 (10):e1005617. doi:10.1371/journal.pgen.1005617

Cui M, Zhang W, Zhang Q, Xu Z, Zhu Z, Duan F, Wu R (2011) Induced over-expression of the transcription factor OsDREB2A improves drought tolerance in rice. Plant Physiol Biochem 49 (12):1384-1391. doi:10.1016/j.plaphy.2011.09.012

Cui XY, Gao Y, Guo J, Yu TF, Zheng WJ, Liu YW, Chen J, Xu ZS, Ma YZ (2019) BES/BZR Transcription Factor TaBZR2 Positively Regulates Drought Responses by Activation of TaGST1. Plant Physiol 180 (1):605-620. doi:10.1104/pp.19.00100

Dai F, Zhang C, Jiang X, Kang M, Yin X, Lu P, Zhang X, Zheng Y, Gao J (2012) RhNAC2 and RhEXPA4 are involved in the regulation of dehydration tolerance during the expansion of rose petals. Plant Physiol 160 (4):2064-2082. doi:10.1104/pp.112.207720

Ding Z, Li S, An X, Liu X, Qin H, Wang D (2009) Transgenic expression of MYB15 confers enhanced sensitivity to abscisic acid and improved drought tolerance in Arabidopsis thaliana. J Genet Genomics 36 (1):17-29. doi:10.1016/S1673-8527(09)60003-5

Djemal R, Khoudi H (2019) Combination of the endogenous promoter-intron significantly improves salt and drought tolerance conferred by TdSHN1 transcription factor in transgenic tobacco. Plant Physiol Biochem 139:435-445. doi:10.1016/j.plaphy.2019.04.009

Djemal R, Khoudi H (2021) The barley SHN1-type transcription factor HvSHN1 imparts heat, drought and salt tolerances in transgenic tobacco. Plant Physiol Biochem 164:44-53. doi:10.1016/j.plaphy.2021.04.018

Dong J, Cao L, Zhang X, Zhang W, Yang T, Zhang J, Che D (2021) An R2R3-MYB Transcription Factor RmMYB108 Responds to Chilling Stress of Rosa multiflora and Conferred Cold Tolerance of Arabidopsis. Front Plant Sci 12:696919. doi:10.3389/fpls.2021.696919

Dong Y, Wang C, Han X, Tang S, Liu S, Xia X, Yin W (2014) A novel bHLH transcription factor PebHLH35 from Populus euphratica confers drought tolerance through regulating stomatal development, photosynthesis and growth in Arabidopsis. Biochem Biophys Res Commun 450 (1):453-458. doi:10.1016/j.bbrc.2014.05.139

Dossa K, Mmadi MA, Zhou R, Liu A, Yang Y, Diouf D, You J, Zhang X (2020) Ectopic expression of the sesame MYB transcription factor SiMYB305 promotes root growth and modulates ABA-mediated tolerance to drought and salt stresses in Arabidopsis. AoB Plants 12 (1):plz081. doi:10.1093/aobpla/plz081

Dou M, Fan S, Yang S, Huang R, Yu H, Feng X (2016) Overexpression of AmRosea1 Gene Confers Drought and Salt Tolerance in Rice. Int J Mol Sci 18 (1). doi:10.3390/ijms18010002

Duan M, Zhang R, Zhu F, Zhang Z, Gou L, Wen J, Dong J, Wang T (2017) A Lipid-Anchored NAC Transcription Factor Is Translocated into the Nucleus and Activates Glyoxalase I Expression during Drought Stress. Plant Cell 29 (7):1748-1772. doi:10.1105/tpc.17.00044

Dubouzet JG, Sakuma Y, Ito Y, Kasuga M, Dubouzet EG, Miura S, Seki M, Shinozaki K, Yamaguchi-Shinozaki K (2003) OsDREB genes in rice, Oryza sativa L., encode transcription activators that function in drought-, high-salt- and cold-responsive gene expression. Plant J 33 (4):751-763. doi:10.1046/j.1365-313x.2003.01661.x

Ebrahimian-Motlagh S, Ribone PA, Thirumalaikumar VP, Allu AD, Chan RL, Mueller-Roeber B, Balazadeh S (2017) JUNGBRUNNEN1 Confers Drought Tolerance Downstream of the HD-Zip I Transcription Factor AtHB13. Front Plant Sci 8:2118. doi:10.3389/fpls.2017.02118

El-Esawi MA, Al-Ghamdi AA, Ali HM, Ahmad M (2019) Overexpression of AtWRKY30 Transcription Factor Enhances Heat and Drought Stress Tolerance in Wheat (Triticum aestivum L.). Genes (Basel) 10 (2). doi:10.3390/genes10020163

El-Esawi MA, Alayafi AA (2019) Overexpression of StDREB2 Transcription Factor Enhances Drought Stress Tolerance in Cotton (Gossypium barbadense L.). Genes (Basel) 10 (2). doi:10.3390/genes10020142

Fan Q, Song A, Jiang J, Zhang T, Sun H, Wang Y, Chen S, Chen F (2016) CmWRKY1 Enhances the Dehydration Tolerance of Chrysanthemum through the Regulation of ABA-Associated Genes. PLoS One 11 (3):e0150572. doi:10.1371/journal.pone.0150572

Fang Q, Wang X, Wang H, Tang X, Liu C, Yin H, Ye S, Jiang Y, Duan Y, Luo K (2020) The poplar R2R3 MYB transcription factor PtrMYB94 coordinates with abscisic acid signaling to improve drought tolerance in plants. Tree Physiol 40 (1):46-59. doi:10.1093/treephys/tpz113

Fang Y, Liao K, Du H, Xu Y, Song H, Li X, Xiong L (2015) A stress-responsive NAC transcription factor SNAC3 confers heat and drought tolerance through modulation of reactive oxygen species in rice. J Exp Bot 66 (21):6803-6817. doi:10.1093/jxb/erv386

Fang Y, Xie K, Xiong L (2014) Conserved miR164-targeted NAC genes negatively regulate drought resistance in rice. J Exp Bot 65 (8):2119-2135. doi:10.1093/jxb/eru072

Favero Peixoto-Junior R, Mara de Andrade L, Dos Santos Brito M, Macedo Nobile P, Palma Boer Martins A, Domingues Carlin S, Vasconcelos Ribeiro R, de Souza Goldman MH, Nebo Carlos de Oliveira JF, Vargas de Oliveira Figueira A, Creste S (2018) Overexpression of ScMYBAS1 alternative splicing transcripts differentially impacts biomass accumulation and drought tolerance in rice transgenic plants. PLoS One 13 (12):e0207534. doi:10.1371/journal.pone.0207534

Feng ZJ, Xu ZS, Sun J, Li LC, Chen M, Yang GX, He GY, Ma YZ (2016) Investigation of the ASR family in foxtail millet and the role of ASR1 in drought/oxidative stress tolerance. Plant Cell Rep 35 (1):115-128. doi:10.1007/s00299-015-1873-y

Ferreira DO, Fraga OT, Pimenta MR, Caetano HDN, Machado JPB, Carpinetti PA, Brustolini OJB, Quadros IPS, Reis PAB, Fontes EPB (2020) GmNAC81 Inversely Modulates Leaf Senescence and Drought Tolerance. Front Genet 11:601876. doi:10.3389/fgene.2020.601876

Fu Y, Ma H, Chen S, Gu T, Gong J (2018) Control of proline accumulation under drought via a novel pathway comprising the histone methylase CAU1 and the transcription factor ANAC055. J Exp Bot 69 (3):579-588. doi:10.1093/jxb/erx419

Gao F, Yao H, Zhao H, Zhou J, Luo X, Huang Y, Li C, Chen H, Wu Q (2016) Tartary buckwheat FtMYB10 encodes an R2R3-MYB transcription factor that acts as a novel negative regulator of salt and drought response in transgenic Arabidopsis. Plant Physiol Biochem 109:387-396. doi:10.1016/j.plaphy.2016.10.022

Gao F, Zhou J, Deng RY, Zhao HX, Li CL, Chen H, Suzuki T, Park SU, Wu Q (2017) Overexpression of a tartary buckwheat R2R3-MYB transcription factor gene, FtMYB9, enhances tolerance to drought and salt stresses in transgenic Arabidopsis. J Plant Physiol 214:81-90. doi:10.1016/j.jplph.2017.04.007

Gao H, Song A, Zhu X, Chen F, Jiang J, Chen Y, Sun Y, Shan H, Gu C, Li P, Chen S (2012) The heterologous expression in Arabidopsis of a chrysanthemum Cys2/His2 zinc finger protein gene confers salinity and drought tolerance. Planta 235 (5):979-993. doi:10.1007/s00425-011-1558-x

Gao H, Wang Y, Xu P, Zhang Z (2018a) Overexpression of a WRKY Transcription Factor TaWRKY2 Enhances Drought Stress Tolerance in Transgenic Wheat. Front Plant Sci 9:997. doi:10.3389/fpls.2018.00997

Gao SQ, Chen M, Xia LQ, Xiu HJ, Xu ZS, Li LC, Zhao CP, Cheng XG, Ma YZ (2009) A cotton (Gossypium hirsutum) DRE-binding transcription factor gene, GhDREB, confers enhanced tolerance to drought, high salt, and freezing stresses in transgenic wheat. Plant Cell Rep 28 (2):301-311. doi:10.1007/s00299-008-0623-9

Gao SQ, Chen M, Xu ZS, Zhao CP, Li L, Xu HJ, Tang YM, Zhao X, Ma YZ (2011) The soybean GmbZIP1 transcription factor enhances multiple abiotic stress tolerances in transgenic plants. Plant Mol Biol 75 (6):537-553. doi:10.1007/s11103-011-9738-4

Gao W, He M, Liu J, Ma X, Zhang Y, Dai S, Zhou Y (2018b) Overexpression of Chrysanthemum lavandulifolium ClCBF1 in Chrysanthemum morifolium 'White Snow' improves the level of salinity and drought tolerance. Plant Physiol Biochem 124:50-58. doi:10.1016/j.plaphy.2018.01.004

Gao Y, Jiang W, Dai Y, Xiao N, Zhang C, Li H, Lu Y, Wu M, Tao X, Deng D, Chen J (2015) A maize phytochrome-interacting factor 3 improves drought and salt stress tolerance in rice. Plant Mol Biol 87 (4-5):413-428. doi:10.1007/s11103-015-0288-z

Gao Y, Liu H, Zhang K, Li F, Wu M, Xiang Y (2021) A moso bamboo transcription factor, Phehdz1, positively regulates the drought stress response of transgenic rice. Plant Cell Rep 40 (1):187-204. doi:10.1007/s00299-020-02625-w

Gao Y, Wu M, Zhang M, Jiang W, Liang E, Zhang D, Zhang C, Xiao N, Chen J (2018c) Roles of a maize phytochrome-interacting factors protein ZmPIF3 in regulation of drought stress responses by controlling stomatal closure in transgenic rice without yield penalty. Plant Mol Biol 97 (4-5):311-323. doi:10.1007/s11103-018-0739-4

Gong L, Zhang H, Liu X, Gan X, Nie F, Yang W, Zhang L, Chen Y, Song Y, Zhang H (2020) Ectopic expression of HaNAC1, an ATAF transcription factor from Haloxylon ammodendron, improves growth and drought tolerance in transgenic Arabidopsis. Plant Physiol Biochem 151:535-544. doi:10.1016/j.plaphy.2020.04.008

Gong X, Zhang J, Hu J, Wang W, Wu H, Zhang Q, Liu JH (2015) FcWRKY70, a WRKY protein of Fortunella crassifolia, functions in drought tolerance and modulates putrescine synthesis by regulating arginine decarboxylase gene. Plant Cell Environ 38 (11):2248-2262. doi:10.1111/pce.12539

Gu L, Ma Q, Zhang C, Wang C, Wei H, Wang H, Yu S (2019) The Cotton GhWRKY91 Transcription Factor Mediates Leaf Senescence and Responses to Drought Stress in Transgenic Arabidopsis thaliana. Front Plant Sci 10:1352. doi:10.3389/fpls.2019.01352

Guan H, Liu X, Niu F, Zhao Q, Fan N, Cao D, Meng D, He W, Guo B, Wei Y, Fu Y (2019) OoNAC72, a NAC-Type Oxytropis ochrocephala Transcription Factor, Conferring Enhanced Drought and Salt Stress Tolerance in Arabidopsis. Front Plant Sci 10:890. doi:10.3389/fpls.2019.00890

Gunapati S, Naresh R, Ranjan S, Nigam D, Hans A, Verma PC, Gadre R, Pathre UV, Sane AP, Sane VA (2016) Expression of GhNAC2 from G. herbaceum, improves root growth and imparts tolerance to drought in transgenic cotton and Arabidopsis. Sci Rep 6:24978. doi:10.1038/srep24978

Guo R, Qiao H, Zhao J, Wang X, Tu M, Guo C, Wan R, Li Z, Wang X (2018) The Grape VlWRKY3 Gene Promotes Abiotic and Biotic Stress Tolerance in Transgenic Arabidopsis thaliana. Front Plant Sci 9:545. doi:10.3389/fpls.2018.00545

Guo Y, Pang C, Jia X, Ma Q, Dou L, Zhao F, Gu L, Wei H, Wang H, Fan S, Su J, Yu S (2017) An NAM Domain Gene, GhNAC79, Improves Resistance to Drought Stress in Upland Cotton. Front Plant Sci 8:1657. doi:10.3389/fpls.2017.01657

Guttikonda SK, Valliyodan B, Neelakandan AK, Tran LS, Kumar R, Quach TN, Voothuluru P, Gutierrez-Gonzalez JJ, Aldrich DL, Pallardy SG, Sharp RE, Ho TH, Nguyen HT (2014) Overexpression of AtDREB1D transcription factor improves drought tolerance in soybean. Mol Biol Rep 41 (12):7995-8008. doi:10.1007/s11033-014-3695-3

Haake V, Cook D, Riechmann JL, Pineda O, Thomashow MF, Zhang JZ (2002) Transcription factor CBF4 is a regulator of drought adaptation in Arabidopsis. Plant Physiol 130 (2):639-648. doi:10.1104/pp.006478

Han T, Yan J, Xiang Y, Zhang A (2021) Phosphorylation of ZmNAC84 at Ser-113 enhances the drought tolerance by directly modulating ZmSOD2 expression in maize. Biochem Biophys Res Commun 567:86-91. doi:10.1016/j.bbrc.2021.06.026

Han X, Tang S, An Y, Zheng DC, Xia XL, Yin WL (2013) Overexpression of the poplar NF-YB7 transcription factor confers drought tolerance and improves water-use efficiency in Arabidopsis. J Exp Bot 64 (14):4589-4601. doi:10.1093/jxb/ert262

He GH, Xu JY, Wang YX, Liu JM, Li PS, Chen M, Ma YZ, Xu ZS (2016) Drought-responsive WRKY transcription factor genes TaWRKY1 and TaWRKY33 from wheat confer drought and/or heat resistance in Arabidopsis. BMC Plant Biol 16 (1):116. doi:10.1186/s12870-016-0806-4

He K, Zhao X, Chi X, Wang Y, Jia C, Zhang H, Zhou G, Hu R (2019) A novel Miscanthus NAC transcription factor MlNAC10 enhances drought and salinity tolerance in transgenic Arabidopsis. J Plant Physiol 233:84-93. doi:10.1016/j.jplph.2019.01.001

He L, Shi X, Wang Y, Guo Y, Yang K, Wang Y (2017) Arabidopsis ANAC069 binds to C[A/G]CG[T/G] sequences to negatively regulate salt and osmotic stress tolerance. Plant Mol Biol 93 (4-5):369-387. doi:10.1007/s11103-016-0567-3

He Q, Cai H, Bai M, Zhang M, Chen F, Huang Y, Priyadarshani S, Chai M, Liu L, Liu Y, Chen H, Qin Y (2020a) A Soybean bZIP Transcription Factor GmbZIP19 Confers Multiple Biotic and Abiotic Stress Responses in Plant. Int J Mol Sci 21 (13). doi:10.3390/ijms21134701

He Y, Mu S, He Z, Wang B, Li Y (2020b) Ectopic expression of MYB repressor GmMYB3a improves drought tolerance and productivity of transgenic peanuts (Arachis hypogaea L.) under conditions of water deficit. Transgenic Res 29 (5-6):563-574. doi:10.1007/s11248-020-00220-z

Hoang XLT, Nguyen NC, Nguyen YH, Watanabe Y, Tran LP, Thao NP (2019) The Soybean GmNAC019 Transcription Factor Mediates Drought Tolerance in Arabidopsis in an Abscisic Acid-Dependent Manner. Int J Mol Sci 21 (1). doi:10.3390/ijms21010286

Hong E, Lim CW, Han SW, Lee SC (2017) Functional Analysis of the Pepper Ethylene-Responsive Transcription Factor, CaAIEF1, in Enhanced ABA Sensitivity and Drought Tolerance. Front Plant Sci 8:1407. doi:10.3389/fpls.2017.01407

Hong Y, Zhang H, Huang L, Li D, Song F (2016) Overexpression of a Stress-Responsive NAC Transcription Factor Gene ONAC022 Improves Drought and Salt Tolerance in Rice. Front Plant Sci 7:4. doi:10.3389/fpls.2016.00004

Hossain MA, Cho JI, Han M, Ahn CH, Jeon JS, An G, Park PB (2010) The ABRE-binding bZIP transcription factor OsABF2 is a positive regulator of abiotic stress and ABA signaling in rice. J Plant Physiol 167 (17):1512-1520. doi:10.1016/j.jplph.2010.05.008

Hou D, Zhao Z, Hu Q, Li L, Vasupalli N, Zhuo J, Zeng W, Wu A, Lin X (2020a) PeSNAC-1 a NAC transcription factor from moso bamboo (Phyllostachys edulis) confers tolerance to salinity and drought stress in transgenic rice. Tree Physiol 40 (12):1792-1806. doi:10.1093/treephys/tpaa099

Hou L, Fan X, Hao J, Liu G, Zhang Z, Liu X (2020b) Negative regulation by transcription factor VvWRKY13 in drought stress of Vitis vinifera L. Plant Physiol Biochem 148:114-121. doi:10.1016/j.plaphy.2020.01.008

Hu H, Dai M, Yao J, Xiao B, Li X, Zhang Q, Xiong L (2006) Overexpressing a NAM, ATAF, and CUC (NAC) transcription factor enhances drought resistance and salt tolerance in rice. Proc Natl Acad Sci U S A 103 (35):12987-12992. doi:10.1073/pnas.0604882103

Hu W, Huang C, Deng X, Zhou S, Chen L, Li Y, Wang C, Ma Z, Yuan Q, Wang Y, Cai R, Liang X, Yang G, He G (2013) TaASR1, a transcription factor gene in wheat, confers drought stress tolerance in transgenic tobacco. Plant Cell Environ 36 (8):1449-1464. doi:10.1111/pce.12074

Huang C, Hu G, Li F, Li Y, Wu J, Zhou X (2013) NbPHAN, a MYB transcriptional factor, regulates leaf development and affects drought tolerance in Nicotiana benthamiana. Physiol Plant 149 (3):297-309. doi:10.1111/ppl.12031

Huang C, Zhou J, Jie Y, Xing H, Zhong Y, Yu W, She W, Ma Y, Liu Z, Zhang Y (2016a) A Ramie bZIP Transcription Factor BnbZIP2 Is Involved in Drought, Salt, and Heavy Metal Stress Response. DNA Cell Biol 35 (12):776-786. doi:10.1089/dna.2016.3251

Huang J, Sun S, Xu D, Lan H, Sun H, Wang Z, Bao Y, Wang J, Tang H, Zhang H (2012) A TFIIIA-type zinc finger protein confers multiple abiotic stress tolerances in transgenic rice (Oryza sativa L.). Plant Mol Biol 80 (3):337-350. doi:10.1007/s11103-012-9955-5

Huang J, Sun SJ, Xu DQ, Yang X, Bao YM, Wang ZF, Tang HJ, Zhang H (2009a) Increased tolerance of rice to cold, drought and oxidative stresses mediated by the overexpression of a gene that encodes the zinc finger protein ZFP245. Biochem Biophys Res Commun 389 (3):556-561. doi:10.1016/j.bbrc.2009.09.032

Huang K, Wu T, Ma Z, Li Z, Chen H, Zhang M, Bian M, Bai H, Jiang W, Du X (2021) Rice Transcription Factor OsWRKY55 Is Involved in the Drought Response and Regulation of Plant Growth. Int J Mol Sci 22 (9). doi:10.3390/ijms22094337

Huang L, Hong Y, Zhang H, Li D, Song F (2016b) Rice NAC transcription factor ONAC095 plays opposite roles in drought and cold stress tolerance. BMC Plant Biol 16 (1):203. doi:10.1186/s12870-016-0897-y

Huang L, Wang Y, Wang W, Zhao X, Qin Q, Sun F, Hu F, Zhao Y, Li Z, Fu B, Li Z (2018a) Characterization of Transcription Factor Gene OsDRAP1 Conferring Drought Tolerance in Rice. Front Plant Sci 9:94. doi:10.3389/fpls.2018.00094

Huang Q, Wang Y (2016) Overexpression of TaNAC2D Displays Opposite Responses to Abiotic Stresses between Seedling and Mature Stage of Transgenic Arabidopsis. Front Plant Sci 7:1754. doi:10.3389/fpls.2016.01754

Huang Q, Wang Y, Li B, Chang J, Chen M, Li K, Yang G, He G (2015) TaNAC29, a NAC transcription factor from wheat, enhances salt and drought tolerance in transgenic Arabidopsis. BMC Plant Biol 15:268. doi:10.1186/s12870-015-0644-9

Huang XS, Liu JH, Chen XJ (2010) Overexpression of PtrABF gene, a bZIP transcription factor isolated from Poncirus trifoliata, enhances dehydration and drought tolerance in tobacco via scavenging ROS and modulating expression of stress-responsive genes. BMC Plant Biol 10:230. doi:10.1186/1471-2229-10-230

Huang XY, Chao DY, Gao JP, Zhu MZ, Shi M, Lin HX (2009b) A previously unknown zinc finger protein, DST, regulates drought and salt tolerance in rice via stomatal aperture control. Genes Dev 23 (15):1805-1817. doi:10.1101/gad.1812409

Huang Y, Zhao H, Gao F, Yao P, Deng R, Li C, Chen H, Wu Q (2018b) A R2R3-MYB transcription factor gene, FtMYB13, from Tartary buckwheat improves salt/drought tolerance in Arabidopsis. Plant Physiol Biochem 132:238-248. doi:10.1016/j.plaphy.2018.09.012

Huang Z, Guo HD, Liu L, Jin SH, Zhu PL, Zhang YP, Jiang CZ (2020) Heterologous Expression of Dehydration-Inducible MfWRKY17 of Myrothamnus Flabellifolia Confers Drought and Salt Tolerance in Arabidopsis. Int J Mol Sci 21 (13). doi:10.3390/ijms21134603

Huo T, Wang CT, Yu TF, Wang DM, Li M, Zhao D, Li XT, Fu JD, Xu ZS, Song XY (2021) Overexpression of ZmWRKY65 transcription factor from maize confers stress resistances in transgenic Arabidopsis. Sci Rep 11 (1):4024. doi:10.1038/s41598-021-83440-5

Jaffar MA, Song A, Faheem M, Chen S, Jiang J, Liu C, Fan Q, Chen F (2016) Involvement of CmWRKY10 in Drought Tolerance of Chrysanthemum through the ABA-Signaling Pathway. Int J Mol Sci 17 (5). doi:10.3390/ijms17050693

Jan A, Maruyama K, Todaka D, Kidokoro S, Abo M, Yoshimura E, Shinozaki K, Nakashima K, Yamaguchi-Shinozaki K (2013) OsTZF1, a CCCH-tandem zinc finger protein, confers delayed senescence and stress tolerance in rice by regulating stress-related genes. Plant Physiol 161 (3):1202-1216. doi:10.1104/pp.112.205385

Jeong JS, Kim YS, Baek KH, Jung H, Ha SH, Do Choi Y, Kim M, Reuzeau C, Kim JK (2010) Root-specific expression of OsNAC10 improves drought tolerance and grain yield in rice under field drought conditions. Plant Physiol 153 (1):185-197. doi:10.1104/pp.110.154773

Ji X, Liu G, Liu Y, Zheng L, Nie X, Wang Y (2013) The bZIP protein from Tamarix hispida, ThbZIP1, is ACGT elements binding factor that enhances abiotic stress signaling in transgenic Arabidopsis. BMC Plant Biol 13:151. doi:10.1186/1471-2229-13-151

Ji XL, Li HL, Qiao ZW, Zhang JC, Sun WJ, Wang CK, Yang K, You CX, Hao YJ (2020) The BTB-TAZ protein MdBT2 negatively regulates the drought stress response by interacting with the transcription factor MdNAC143 in apple. Plant Sci 301:110689. doi:10.1016/j.plantsci.2020.110689

Jia D, Jiang Q, van Nocker S, Gong X, Ma F (2019) An apple (Malus domestica) NAC transcription factor enhances drought tolerance in transgenic apple plants. Plant Physiol Biochem 139:504-512. doi:10.1016/j.plaphy.2019.04.011

Jia H, Wang C, Wang F, Liu S, Li G, Guo X (2015) GhWRKY68 reduces resistance to salt and drought in transgenic Nicotiana benthamiana. PLoS One 10 (3):e0120646. doi:10.1371/journal.pone.0120646

Jian W, Zheng Y, Yu T, Cao H, Chen Y, Cui Q, Xu C, Li Z (2021) SlNAC6, A NAC transcription factor, is involved in drought stress response and reproductive process in tomato. J Plant Physiol 264:153483. doi:10.1016/j.jplph.2021.153483

Jiang L, Tian X, Li S, Fu Y, Xu J, Wang G (2019) The AabHLH35 Transcription Factor Identified from Anthurium andraeanum is Involved in Cold and Drought Tolerance. Plants (Basel) 8 (7). doi:10.3390/plants8070216

Jiang X, Zhang C, Lu P, Jiang G, Liu X, Dai F, Gao J (2014) RhNAC3, a stress-associated NAC transcription factor, has a role in dehydration tolerance through regulating osmotic stress-related genes in rose petals. Plant Biotechnol J 12 (1):38-48. doi:10.1111/pbi.12114

Jin C, Li KQ, Xu XY, Zhang HP, Chen HX, Chen YH, Hao J, Wang Y, Huang XS, Zhang SL (2017) A Novel NAC Transcription Factor, PbeNAC1, of Pyrus betulifolia Confers Cold and Drought Tolerance via Interacting with PbeDREBs and Activating the Expression of Stress-Responsive Genes. Front Plant Sci 8:1049. doi:10.3389/fpls.2017.01049

Jin X, Xue Y, Wang R, Xu R, Bian L, Zhu B, Han H, Peng R, Yao Q (2013) Transcription factor OsAP21 gene increases salt/drought tolerance in transgenic Arabidopsis thaliana. Mol Biol Rep 40 (2):1743-1752. doi:10.1007/s11033-012-2228-1

Jin Y, Pan W, Zheng X, Cheng X, Liu M, Ma H, Ge X (2018) OsERF101, an ERF family transcription factor, regulates drought stress response in reproductive tissues. Plant Mol Biol 98 (1-2):51-65. doi:10.1007/s11103-018-0762-5

Jisha V, Dampanaboina L, Vadassery J, Mithofer A, Kappara S, Ramanan R (2015) Overexpression of an AP2/ERF Type Transcription Factor OsEREBP1 Confers Biotic and Abiotic Stress Tolerance in Rice. PLoS One 10 (6):e0127831. doi:10.1371/journal.pone.0127831

Joo H, Lim CW, Lee SC (2019a) Roles of pepper bZIP transcription factor CaATBZ1 and its interacting partner RING-type E3 ligase CaASRF1 in modulation of ABA signalling and drought tolerance. Plant J 100 (2):399-410. doi:10.1111/tpj.14451

Joo J, Lee YH, Song SI (2019b) OsbZIP42 is a positive regulator of ABA signaling and confers drought tolerance to rice. Planta 249 (5):1521-1533. doi:10.1007/s00425-019-03104-7

Ju YL, Min Z, Yue XF, Zhang YL, Zhang JX, Zhang ZQ, Fang YL (2020a) Overexpression of grapevine VvNAC08 enhances drought tolerance in transgenic Arabidopsis. Plant Physiol Biochem 151:214-222. doi:10.1016/j.plaphy.2020.03.028

Ju YL, Yue XF, Min Z, Wang XH, Fang YL, Zhang JX (2020b) VvNAC17, a novel stress-responsive grapevine (Vitis vinifera L.) NAC transcription factor, increases sensitivity to abscisic acid and enhances salinity, freezing, and drought tolerance in transgenic Arabidopsis. Plant Physiol Biochem 146:98-111. doi:10.1016/j.plaphy.2019.11.002

Jung C, Seo JS, Han SW, Koo YJ, Kim CH, Song SI, Nahm BH, Choi YD, Cheong JJ (2008) Overexpression of AtMYB44 enhances stomatal closure to confer abiotic stress tolerance in transgenic Arabidopsis. Plant Physiol 146 (2):623-635. doi:10.1104/pp.107.110981

Jung H, Chung PJ, Park SH, Redillas M, Kim YS, Suh JW, Kim JK (2017) Overexpression of OsERF48 causes regulation of OsCML16, a calmodulin-like protein gene that enhances root growth and drought tolerance. Plant Biotechnol J 15 (10):1295-1308. doi:10.1111/pbi.12716

Jung SE, Bang SW, Kim SH, Seo JS, Yoon HB, Kim YS, Kim JK (2021) Overexpression of OsERF83, a Vascular Tissue-Specific Transcription Factor Gene, Confers Drought Tolerance in Rice. Int J Mol Sci 22 (14). doi:10.3390/ijms22147656

Kang C, Zhai H, He S, Zhao N, Liu Q (2019) A novel sweetpotato bZIP transcription factor gene, IbbZIP1, is involved in salt and drought tolerance in transgenic Arabidopsis. Plant Cell Rep 38 (11):1373-1382. doi:10.1007/s00299-019-02441-x

Kang HG, Kim J, Kim B, Jeong H, Choi SH, Kim EK, Lee HY, Lim PO (2011) Overexpression of FTL1/DDF1, an AP2 transcription factor, enhances tolerance to cold, drought, and heat stresses in Arabidopsis thaliana. Plant Sci 180 (4):634-641. doi:10.1016/j.plantsci.2011.01.002

Karaba A, Dixit S, Greco R, Aharoni A, Trijatmiko KR, Marsch-Martinez N, Krishnan A, Nataraja KN, Udayakumar M, Pereira A (2007) Improvement of water use efficiency in rice by expression of HARDY, an Arabidopsis drought and salt tolerance gene. Proc Natl Acad Sci U S A 104 (39):15270-15275. doi:10.1073/pnas.0707294104

Kasuga M, Liu Q, Miura S, Yamaguchi-Shinozaki K, Shinozaki K (1999) Improving plant drought, salt, and freezing tolerance by gene transfer of a single stress-inducible transcription factor. Nat Biotechnol 17 (3):287-291. doi:10.1038/7036

Kerr TCC, Abdel-Mageed H, Aleman L, Lee J, Payton P, Cryer D, Allen RD (2018) Ectopic expression of two AREB/ABF orthologs increases drought tolerance in cotton (Gossypium hirsutum). Plant Cell Environ 41 (5):898-907. doi:10.1111/pce.12906

Khan IU, Ali A, Khan HA, Baek D, Park J, Lim CJ, Zareen S, Jan M, Lee SY, Pardo JM, Kim WY, Yun DJ (2020) PWR/HDA9/ABI4 Complex Epigenetically Regulates ABA Dependent Drought Stress Tolerance in Arabidopsis. Front Plant Sci 11:623. doi:10.3389/fpls.2020.00623

Kim MJ, Park MJ, Seo PJ, Song JS, Kim HJ, Park CM (2012) Controlled nuclear import of the transcription factor NTL6 reveals a cytoplasmic role of SnRK2.8 in the drought-stress response. Biochem J 448 (3):353-363. doi:10.1042/BJ20120244

Kiranmai K, Lokanadha Rao G, Pandurangaiah M, Nareshkumar A, Amaranatha Reddy V, Lokesh U, Venkatesh B, Anthony Johnson AM, Sudhakar C (2018) A Novel WRKY Transcription Factor, MuWRKY3 (Macrotyloma uniflorum Lam. Verdc.) Enhances Drought Stress Tolerance in Transgenic Groundnut (Arachis hypogaea L.) Plants. Front Plant Sci 9:346. doi:10.3389/fpls.2018.00346

Kobayashi F, Maeta E, Terashima A, Takumi S (2008) Positive role of a wheat HvABI5 ortholog in abiotic stress response of seedlings. Physiol Plant 134 (1):74-86. doi:10.1111/j.1399-3054.2008.01107.x

Le Hir R, Castelain M, Chakraborti D, Moritz T, Dinant S, Bellini C (2017) AtbHLH68 transcription factor contributes to the regulation of ABA homeostasis and drought stress tolerance in Arabidopsis thaliana. Physiol Plant 160 (3):312-327. doi:10.1111/ppl.12549

Lee DK, Chung PJ, Jeong JS, Jang G, Bang SW, Jung H, Kim YS, Ha SH, Choi YD, Kim JK (2017) The rice OsNAC6 transcription factor orchestrates multiple molecular mechanisms involving root structural adaptions and nicotianamine biosynthesis for drought tolerance. Plant Biotechnol J 15 (6):754-764. doi:10.1111/pbi.12673

Lee DK, Jung H, Jang G, Jeong JS, Kim YS, Ha SH, Do Choi Y, Kim JK (2016) Overexpression of the OsERF71 Transcription Factor Alters Rice Root Structure and Drought Resistance. Plant Physiol 172 (1):575-588. doi:10.1104/pp.16.00379

Lee DK, Kim HI, Jang G, Chung PJ, Jeong JS, Kim YS, Bang SW, Jung H, Choi YD, Kim JK (2015a) The NF-YA transcription factor OsNF-YA7 confers drought stress tolerance of rice in an abscisic acid independent manner. Plant Sci 241:199-210. doi:10.1016/j.plantsci.2015.10.006

Lee H, Cha J, Choi C, Choi N, Ji HS, Park SR, Lee S, Hwang DJ (2018) Rice WRKY11 Plays a Role in Pathogen Defense and Drought Tolerance. Rice (N Y) 11 (1):5. doi:10.1186/s12284-018-0199-0

Lee S, Seo PJ, Lee HJ, Park CM (2012) A NAC transcription factor NTL4 promotes reactive oxygen species production during drought-induced leaf senescence in Arabidopsis. Plant J 70 (5):831-844. doi:10.1111/j.1365-313X.2012.04932.x

Lee SB, Lee SJ, Kim SY (2015b) AtERF15 is a positive regulator of ABA response. Plant Cell Rep 34 (1):71-81. doi:10.1007/s00299-014-1688-2

Lee SC, Choi HW, Hwang IS, Choi DS, Hwang BK (2006) Functional roles of the pepper pathogen-induced bZIP transcription factor, CAbZIP1, in enhanced resistance to pathogen infection and environmental stresses. Planta 224 (5):1209-1225. doi:10.1007/s00425-006-0302-4

Li B, Zheng JC, Wang TT, Min DH, Wei WL, Chen J, Zhou YB, Chen M, Xu ZS, Ma YZ (2020a) Expression Analyses of Soybean VOZ Transcription Factors and the Role of GmVOZ1G in Drought and Salt Stress Tolerance. Int J Mol Sci 21 (6). doi:10.3390/ijms21062177

Li HC, Zhang HN, Li GL, Liu ZH, Zhang YM, Zhang HM, Guo XL (2015a) Expression of maize heat shock transcription factor gene ZmHsf06 enhances the thermotolerance and drought-stress tolerance of transgenic Arabidopsis. Funct Plant Biol 42 (11):1080-1091. doi:10.1071/FP15080

Li J, Dong Y, Li C, Pan Y, Yu J (2016a) SiASR4, the Target Gene of SiARDP from Setaria italica, Improves Abiotic Stress Adaption in Plants. Front Plant Sci 7:2053. doi:10.3389/fpls.2016.02053

Li J, Li Y, Yin Z, Jiang J, Zhang M, Guo X, Ye Z, Zhao Y, Xiong H, Zhang Z, Shao Y, Jiang C, Zhang H, An G, Paek NC, Ali J, Li Z (2017a) OsASR5 enhances drought tolerance through a stomatal closure pathway associated with ABA and H2 O2 signalling in rice. Plant Biotechnol J 15 (2):183-196. doi:10.1111/pbi.12601

Li J, Zhao S, Yu X, Du W, Li H, Sun Y, Sun H, Ruan C (2021a) Role of Xanthoceras sorbifolium MYB44 in tolerance to combined drought and heat stress via modulation of stomatal closure and ROS homeostasis. Plant Physiol Biochem 162:410-420. doi:10.1016/j.plaphy.2021.03.007

Li JB, Luan YS, Liu Z (2015b) Overexpression of SpWRKY1 promotes resistance to Phytophthora nicotianae and tolerance to salt and drought stress in transgenic tobacco. Physiol Plant 155 (3):248-266. doi:10.1111/ppl.12315

Li JB, Luan YS, Yin YL (2014a) SpMYB overexpression in tobacco plants leads to altered abiotic and biotic stress responses. Gene 547 (1):145-151. doi:10.1016/j.gene.2014.06.049

Li JB, Ni Dong X, Lei Z, Liang Li Y, Yang Yang P, Tao F, Zhao L, Li SG, Du LF, Shao JR, Wu YM (2016b) Simultaneous Overexpression of the HhERF2 and PeDREB2a Genes Enhanced Tolerances to Salt and Drought in Transgenic Cotton. Protein Pept Lett 23 (5):450-458. doi:10.2174/0929866523666160314153212

Li K, Xing C, Yao Z, Huang X (2017b) PbrMYB21, a novel MYB protein of Pyrus betulaefolia, functions in drought tolerance and modulates polyamine levels by regulating arginine decarboxylase gene. Plant Biotechnol J 15 (9):1186-1203. doi:10.1111/pbi.12708

Li MJ, Qiao Y, Li YQ, Shi ZL, Zhang N, Bi CL, Guo JK (2016c) A R2R3-MYB transcription factor gene in common wheat (namely TaMYBsm1) involved in enhancement of drought tolerance in transgenic Arabidopsis. J Plant Res 129 (6):1097-1107. doi:10.1007/s10265-016-0857-5

Li MR, Li Y, Li HQ, Wu GJ (2011) Ectopic expression of FaDREB2 enhances osmotic tolerance in paper mulberry. J Integr Plant Biol 53 (12):951-960. doi:10.1111/j.1744-7909.2011.01087.x

Li P, Zhang B, Su T, Li P, Xin X, Wang W, Zhao X, Yu Y, Zhang D, Yu S, Zhang F (2018a) BrLAS, a GRAS Transcription Factor From Brassica rapa, Is Involved in Drought Stress Tolerance in Transgenic Arabidopsis. Front Plant Sci 9:1792. doi:10.3389/fpls.2018.01792

Li PS, Yu TF, He GH, Chen M, Zhou YB, Chai SC, Xu ZS, Ma YZ (2014b) Genome-wide analysis of the Hsf family in soybean and functional identification of GmHsf-34 involvement in drought and heat stresses. BMC Genomics 15:1009. doi:10.1186/1471-2164-15-1009

Li Q, Wu Q, Wang A, Lv B, Dong Q, Yao Y, Wu Q, Zhao H, Li C, Chen H, Wang X (2019a) Tartary buckwheat transcription factor FtbZIP83 improves the drought/salt tolerance of Arabidopsis via an ABA-mediated pathway. Plant Physiol Biochem 144:312-323. doi:10.1016/j.plaphy.2019.10.003

Li Q, Zhao H, Wang X, Kang J, Lv B, Dong Q, Li C, Chen H, Wu Q (2020b) Tartary Buckwheat Transcription Factor FtbZIP5, Regulated by FtSnRK2.6, Can Improve Salt/Drought Resistance in Transgenic Arabidopsis. Int J Mol Sci 21 (3). doi:10.3390/ijms21031123

Li S, Chen N, Li F, Mei F, Wang Z, Cheng X, Kang Z, Mao H (2020c) Characterization of wheat homeodomain-leucine zipper family genes and functional analysis of TaHDZ5-6A in drought tolerance in transgenic Arabidopsis. BMC Plant Biol 20 (1):50. doi:10.1186/s12870-020-2252-6

Li S, Lin YJ, Wang P, Zhang B, Li M, Chen S, Shi R, Tunlaya-Anukit S, Liu X, Wang Z, Dai X, Yu J, Zhou C, Liu B, Wang JP, Chiang VL, Li W (2019b) The AREB1 Transcription Factor Influences Histone Acetylation to Regulate Drought Responses and Tolerance in Populus trichocarpa. Plant Cell 31 (3):663-686. doi:10.1105/tpc.18.00437

Li WX, Oono Y, Zhu J, He XJ, Wu JM, Iida K, Lu XY, Cui X, Jin H, Zhu JK (2008) The Arabidopsis NFYA5 transcription factor is regulated transcriptionally and posttranscriptionally to promote drought resistance. Plant Cell 20 (8):2238-2251. doi:10.1105/tpc.108.059444

Li X, Feng B, Zhang F, Tang Y, Zhang L, Ma L, Zhao C, Gao S (2016d) Bioinformatic Analyses of Subgroup-A Members of the Wheat bZIP Transcription Factor Family and Functional Identification of TabZIP174 Involved in Drought Stress Response. Front Plant Sci 7:1643. doi:10.3389/fpls.2016.01643

Li X, Tang Y, Li H, Luo W, Zhou C, Zhang L, Lv J (2020d) A wheat R2R3 MYB gene TaMpc1-D4 negatively regulates drought tolerance in transgenic Arabidopsis and wheat. Plant Sci 299:110613. doi:10.1016/j.plantsci.2020.110613

Li X, Tang Y, Zhou C, Zhang L, Lv J (2020e) A Wheat WRKY Transcription Factor TaWRKY46 Enhances Tolerance to Osmotic Stress in transgenic Arabidopsis Plants. Int J Mol Sci 21 (4). doi:10.3390/ijms21041321

Li X, Zhong M, Qu L, Yang J, Liu X, Zhao Q, Liu X, Zhao X (2021b) AtMYB32 regulates the ABA response by targeting ABI3, ABI4 and ABI5 and the drought response by targeting CBF4 in Arabidopsis. Plant Sci 310:110983. doi:10.1016/j.plantsci.2021.110983

Li XL, Yang X, Hu YX, Yu XD, Li QL (2014c) A novel NAC transcription factor from Suaeda liaotungensis K. enhanced transgenic Arabidopsis drought, salt, and cold stress tolerance. Plant Cell Rep 33 (5):767-778. doi:10.1007/s00299-014-1602-y

Li XY, Liu X, Yao Y, Li YH, Liu S, He CY, Li JM, Lin YY, Li L (2013) Overexpression of Arachis hypogaea AREB1 gene enhances drought tolerance by modulating ROS scavenging and maintaining endogenous ABA content. Int J Mol Sci 14 (6):12827-12842. doi:10.3390/ijms140612827

Li Y, Chen Q, Nan H, Li X, Lu S, Zhao X, Liu B, Guo C, Kong F, Cao D (2017c) Overexpression of GmFDL19 enhances tolerance to drought and salt stresses in soybean. PLoS One 12 (6):e0179554. doi:10.1371/journal.pone.0179554

Li Y, Zhang H, Zhang Q, Liu Q, Zhai H, Zhao N, He S (2019c) An AP2/ERF gene, IbRAP2-12, from sweetpotato is involved in salt and drought tolerance in transgenic Arabidopsis. Plant Sci 281:19-30. doi:10.1016/j.plantsci.2019.01.009

Li Z, Liang F, Zhang T, Fu N, Pei X, Long Y (2021c) Enhanced tolerance to drought stress resulting from Caragana korshinskii CkWRKY33 in transgenic Arabidopsis thaliana. BMC Genom Data 22 (1):11. doi:10.1186/s12863-021-00965-4

Li Z, Liu C, Zhang Y, Wang B, Ran Q, Zhang J (2019d) The bHLH family member ZmPTF1 regulates drought tolerance in maize by promoting root development and abscisic acid synthesis. J Exp Bot 70 (19):5471-5486. doi:10.1093/jxb/erz307

Li Z, Tian Y, Xu J, Fu X, Gao J, Wang B, Han H, Wang L, Peng R, Yao Q (2018b) A tomato ERF transcription factor, SlERF84, confers enhanced tolerance to drought and salt stress but negatively regulates immunity against Pseudomonas syringae pv. tomato DC3000. Plant Physiol Biochem 132:683-695. doi:10.1016/j.plaphy.2018.08.022

Liang C, Meng Z, Meng Z, Malik W, Yan R, Lwin KM, Lin F, Wang Y, Sun G, Zhou T, Zhu T, Li J, Jin S, Guo S, Zhang R (2016) GhABF2, a bZIP transcription factor, confers drought and salinity tolerance in cotton (Gossypium hirsutum L.). Sci Rep 6:35040. doi:10.1038/srep35040

Liang J, Zheng J, Wu Z, Wang H (2020) Strawberry FaNAC2 Enhances Tolerance to Abiotic Stress by Regulating Proline Metabolism. Plants (Basel) 9 (11). doi:10.3390/plants9111417

Liang Y, Jiang Y, Du M, Li B, Chen L, Chen M, Jin D, Wu J (2019) ZmASR3 from the Maize ASR Gene Family Positively Regulates Drought Tolerance in Transgenic Arabidopsis. Int J Mol Sci 20 (9). doi:10.3390/ijms20092278

Lim CW, Baek W, Lee SC (2018) Roles of pepper bZIP protein CaDILZ1 and its interacting partner RING-type E3 ligase CaDSR1 in modulation of drought tolerance. Plant J 96 (2):452-467. doi:10.1111/tpj.14046

Lin J, Liu D, Wang X, Ahmed S, Li M, Kovinich N, Sui S (2021) Transgene CpNAC68 from Wintersweet (Chimonanthus praecox) Improves Arabidopsis Survival of Multiple Abiotic Stresses. Plants (Basel) 10 (7). doi:10.3390/plants10071403

Lin RC, Park HJ, Wang HY (2008) Role of Arabidopsis RAP2.4 in regulating light- and ethylene-mediated developmental processes and drought stress tolerance. Mol Plant 1 (1):42-57. doi:10.1093/mp/ssm004

Liu B, Ouyang Z, Zhang Y, Li X, Hong Y, Huang L, Liu S, Zhang H, Li D, Song F (2014a) Tomato NAC transcription factor SlSRN1 positively regulates defense response against biotic stress but negatively regulates abiotic stress response. PLoS One 9 (7):e102067. doi:10.1371/journal.pone.0102067

Liu C, Ma H, Zhou J, Li Z, Peng Z, Guo F, Zhang J (2019a) TsHD1 and TsNAC1 cooperatively play roles in plant growth and abiotic stress resistance of Thellungiella halophile. Plant J 99 (1):81-97. doi:10.1111/tpj.14310

Liu C, Mao B, Ou S, Wang W, Liu L, Wu Y, Chu C, Wang X (2014b) OsbZIP71, a bZIP transcription factor, confers salinity and drought tolerance in rice. Plant Mol Biol 84 (1-2):19-36. doi:10.1007/s11103-013-0115-3

Liu C, Wu Y, Wang X (2012) bZIP transcription factor OsbZIP52/RISBZ5: a potential negative regulator of cold and drought stress response in rice. Planta 235 (6):1157-1169. doi:10.1007/s00425-011-1564-z

Liu G, Li B, Li X, Wei Y, He C, Shi H (2020a) MaWRKY80 positively regulates plant drought stress resistance through modulation of abscisic acid and redox metabolism. Plant Physiol Biochem 156:155-166. doi:10.1016/j.plaphy.2020.09.015

Liu G, Li X, Jin S, Liu X, Zhu L, Nie Y, Zhang X (2014c) Overexpression of rice NAC gene SNAC1 improves drought and salt tolerance by enhancing root development and reducing transpiration rate in transgenic cotton. PLoS One 9 (1):e86895. doi:10.1371/journal.pone.0086895

Liu H, Yang Y, Liu D, Wang X, Zhang L (2020b) Transcription factor TabHLH49 positively regulates dehydrin WZY2 gene expression and enhances drought stress tolerance in wheat. BMC Plant Biol 20 (1):259. doi:10.1186/s12870-020-02474-5

Liu HY, Dai JR, Feng DR, Liu B, Wang HB, Wang JF (2010) Characterization of a novel plantain Asr gene, MpAsr, that is regulated in response to infection of Fusarium oxysporum f. sp. cubense and abiotic stresses. J Integr Plant Biol 52 (3):315-323. doi:10.1111/j.1744-7909.2010.00912.x

Liu J, Chu J, Ma C, Jiang Y, Ma Y, Xiong J, Cheng ZM (2019b) Overexpression of an ABA-dependent grapevine bZIP transcription factor, VvABF2, enhances osmotic stress in Arabidopsis. Plant Cell Rep 38 (5):587-596. doi:10.1007/s00299-019-02389-y

Liu QL, Xu KD, Zhong M, Pan YZ, Jiang BB, Liu GL, Jia Y, Zhang HQ (2013a) Overexpression of a novel chrysanthemum Cys2/His2-type zinc finger protein gene DgZFP3 confers drought tolerance in tobacco. Biotechnol Lett 35 (11):1953-1959. doi:10.1007/s10529-013-1289-0

Liu T, Longhurst AD, Talavera-Rauh F, Hokin SA, Barton MK (2016a) The Arabidopsis transcription factor ABIG1 relays ABA signaled growth inhibition and drought induced senescence. Elife 5. doi:10.7554/eLife.13768

Liu W, Tai H, Li S, Gao W, Zhao M, Xie C, Li WX (2014d) bHLH122 is important for drought and osmotic stress resistance in Arabidopsis and in the repression of ABA catabolism. New Phytol 201 (4):1192-1204. doi:10.1111/nph.12607

Liu W, Zhao BG, Chao Q, Wang B, Zhang Q, Zhang C, Li S, Jin F, Yang D, Li X (2019c) Function analysis of ZmNAC33, a positive regulator in drought stress response in Arabidopsis. Plant Physiol Biochem 145:174-183. doi:10.1016/j.plaphy.2019.10.038

Liu WX, Zhang FC, Zhang WZ, Song LF, Wu WH, Chen YF (2013b) Arabidopsis Di19 functions as a transcription factor and modulates PR1, PR2, and PR5 expression in response to drought stress. Mol Plant 6 (5):1487-1502. doi:10.1093/mp/sst031

Liu X, Li R, Dai Y, Yuan L, Sun Q, Zhang S, Wang X (2019d) A B-box zinc finger protein, MdBBX10, enhanced salt and drought stresses tolerance in Arabidopsis. Plant Mol Biol 99 (4-5):437-447. doi:10.1007/s11103-019-00828-8

Liu X, Liu S, Wu J, Zhang B, Li X, Yan Y, Li L (2013c) Overexpression of Arachis hypogaea NAC3 in tobacco enhances dehydration and drought tolerance by increasing superoxide scavenging. Plant Physiol Biochem 70:354-359. doi:10.1016/j.plaphy.2013.05.018

Liu X, Song Y, Xing F, Wang N, Wen F, Zhu C (2016b) GhWRKY25, a group I WRKY gene from cotton, confers differential tolerance to abiotic and biotic stresses in transgenic Nicotiana benthamiana. Protoplasma 253 (5):1265-1281. doi:10.1007/s00709-015-0885-3

Liu Y, Ji X, Nie X, Qu M, Zheng L, Tan Z, Zhao H, Huo L, Liu S, Zhang B, Wang Y (2015) Arabidopsis AtbHLH112 regulates the expression of genes involved in abiotic stress tolerance by binding to their E-box and GCG-box motifs. New Phytol 207 (3):692-709. doi:10.1111/nph.13387

Liu Y, Yang T, Lin Z, Gu B, Xing C, Zhao L, Dong H, Gao J, Xie Z, Zhang S, Huang X (2019e) A WRKY transcription factor PbrWRKY53 from Pyrus betulaefolia is involved in drought tolerance and AsA accumulation. Plant Biotechnol J 17 (9):1770-1787. doi:10.1111/pbi.13099

Lu G, Gao C, Zheng X, Han B (2009) Identification of OsbZIP72 as a positive regulator of ABA response and drought tolerance in rice. Planta 229 (3):605-615. doi:10.1007/s00425-008-0857-3

Lu M, Ying S, Zhang DF, Shi YS, Song YC, Wang TY, Li Y (2012) A maize stress-responsive NAC transcription factor, ZmSNAC1, confers enhanced tolerance to dehydration in transgenic Arabidopsis. Plant Cell Rep 31 (9):1701-1711. doi:10.1007/s00299-012-1284-2

Lu X, Dun H, Lian C, Zhang X, Yin W, Xia X (2017) The role of peu-miR164 and its target PeNAC genes in response to abiotic stress in Populus euphratica. Plant Physiol Biochem 115:418-438. doi:10.1016/j.plaphy.2017.04.009

Lu X, Zhang X, Duan H, Lian C, Liu C, Yin W, Xia X (2018) Three stress-responsive NAC transcription factors from Populus euphratica differentially regulate salt and drought tolerance in transgenic plants. Physiol Plant 162 (1):73-97. doi:10.1111/ppl.12613

Luo X, Bai X, Sun X, Zhu D, Liu B, Ji W, Cai H, Cao L, Wu J, Hu M, Liu X, Tang L, Zhu Y (2013) Expression of wild soybean WRKY20 in Arabidopsis enhances drought tolerance and regulates ABA signalling. J Exp Bot 64 (8):2155-2169. doi:10.1093/jxb/ert073

Luo X, Bai X, Zhu D, Li Y, Ji W, Cai H, Wu J, Liu B, Zhu Y (2012) GsZFP1, a new Cys2/His2-type zinc-finger protein, is a positive regulator of plant tolerance to cold and drought stress. Planta 235 (6):1141-1155. doi:10.1007/s00425-011-1563-0

Luo X, Li C, He X, Zhang X, Zhu L (2020) ABA signaling is negatively regulated by GbWRKY1 through JAZ1 and ABI1 to affect salt and drought tolerance. Plant Cell Rep 39 (2):181-194. doi:10.1007/s00299-019-02480-4

Lv Z, Wang S, Zhang F, Chen L, Hao X, Pan Q, Fu X, Li L, Sun X, Tang K (2016) Overexpression of a Novel NAC Domain-Containing Transcription Factor Gene (AaNAC1) Enhances the Content of Artemisinin and Increases Tolerance to Drought and Botrytis cinerea in Artemisia annua. Plant Cell Physiol 57 (9):1961-1971. doi:10.1093/pcp/pcw118

Ma H, Liu C, Li Z, Ran Q, Xie G, Wang B, Fang S, Chu J, Zhang J (2018a) ZmbZIP4 Contributes to Stress Resistance in Maize by Regulating ABA Synthesis and Root Development. Plant Physiol 178 (2):753-770. doi:10.1104/pp.18.00436

Ma J, Gao X, Liu Q, Shao Y, Zhang D, Jiang L, Li C (2017) Overexpression of TaWRKY146 Increases Drought Tolerance through Inducing Stomatal Closure in Arabidopsis thaliana. Front Plant Sci 8:2036. doi:10.3389/fpls.2017.02036

Ma J, Wang LY, Dai JX, Wang Y, Lin D (2021) The NAC-type transcription factor CaNAC46 regulates the salt and drought tolerance of transgenic Arabidopsis thaliana. BMC Plant Biol 21 (1):11. doi:10.1186/s12870-020-02764-y

Ma Q, Xia Z, Cai Z, Li L, Cheng Y, Liu J, Nian H (2018b) GmWRKY16 Enhances Drought and Salt Tolerance Through an ABA-Mediated Pathway in Arabidopsis thaliana. Front Plant Sci 9:1979. doi:10.3389/fpls.2018.01979

Ma T, Li M, Zhao A, Xu X, Liu G, Cheng L (2014) LcWRKY5: an unknown function gene from sheepgrass improves drought tolerance in transgenic Arabidopsis. Plant Cell Rep 33 (9):1507-1518. doi:10.1007/s00299-014-1634-3

Ma X, Li C, Wang M (2015) Wheat NF-YA10 functions independently in salinity and drought stress. Bioengineered 6 (4):245-247. doi:10.1080/21655979.2015.1054085

Ma XJ, Yu TF, Li XH, Cao XY, Ma J, Chen J, Zhou YB, Chen M, Ma YZ, Zhang JH, Xu ZS (2020) Overexpression of GmNFYA5 confers drought tolerance to transgenic Arabidopsis and soybean plants. BMC Plant Biol 20 (1):123. doi:10.1186/s12870-020-02337-z

Manavella PA, Arce AL, Dezar CA, Bitton F, Renou JP, Crespi M, Chan RL (2006) Cross-talk between ethylene and drought signalling pathways is mediated by the sunflower Hahb-4 transcription factor. Plant J 48 (1):125-137. doi:10.1111/j.1365-313X.2006.02865.x

Mao H, Wang H, Liu S, Li Z, Yang X, Yan J, Li J, Tran LS, Qin F (2015) A transposable element in a NAC gene is associated with drought tolerance in maize seedlings. Nat Commun 6:8326. doi:10.1038/ncomms9326

Mao H, Yu L, Han R, Li Z, Liu H (2016) ZmNAC55, a maize stress-responsive NAC transcription factor, confers drought resistance in transgenic Arabidopsis. Plant Physiol Biochem 105:55-66. doi:10.1016/j.plaphy.2016.04.018

Mao X, Chen S, Li A, Zhai C, Jing R (2014) Novel NAC transcription factor TaNAC67 confers enhanced multi-abiotic stress tolerances in Arabidopsis. PLoS One 9 (1):e84359. doi:10.1371/journal.pone.0084359

Mao X, Zhang H, Qian X, Li A, Zhao G, Jing R (2012) TaNAC2, a NAC-type wheat transcription factor conferring enhanced multiple abiotic stress tolerances in Arabidopsis. J Exp Bot 63 (8):2933-2946. doi:10.1093/jxb/err462

Mao Y, Xu J, Wang Q, Li G, Tang X, Liu T, Feng X, Wu F, Li M, Xie W, Lu Y (2021) A natural antisense transcript acts as a negative regulator for the maize drought stress response gene ZmNAC48. J Exp Bot 72 (7):2790-2806. doi:10.1093/jxb/erab023

Meena RP, Vishwakarma H, Ghosh G, Gaikwad K, Chellapilla TS, Singh MP, Padaria JC (2020) Novel ASR isolated from drought stress responsive SSH library in pearl millet confers multiple abiotic stress tolerance in PgASR3 transgenic Arabidopsis. Plant Physiol Biochem 156:7-19. doi:10.1016/j.plaphy.2020.07.031

Mei F, Chen B, Li F, Zhang Y, Kang Z, Wang X, Mao H (2021) Overexpression of the wheat NAC transcription factor TaSNAC4-3A gene confers drought tolerance in transgenic Arabidopsis. Plant Physiol Biochem 160:37-50. doi:10.1016/j.plaphy.2021.01.004

Meng S, Cao Y, Li H, Bian Z, Wang D, Lian C, Yin W, Xia X (2019) PeSHN1 regulates water-use efficiency and drought tolerance by modulating wax biosynthesis in poplar. Tree Physiol 39 (8):1371-1386. doi:10.1093/treephys/tpz033

Minh-Thu PT, Kim JS, Chae S, Jun KM, Lee GS, Kim DE, Cheong JJ, Song SI, Nahm BH, Kim YK (2018) A WUSCHEL Homeobox Transcription Factor, OsWOX13, Enhances Drought Tolerance and Triggers Early Flowering in Rice. Mol Cells 41 (8):781-798. doi:10.14348/molcells.2018.0203

Mittal A, Gampala SS, Ritchie GL, Payton P, Burke JJ, Rock CD (2014) Related to ABA-Insensitive3(ABI3)/Viviparous1 and AtABI5 transcription factor coexpression in cotton enhances drought stress adaptation. Plant Biotechnol J 12 (5):578-589. doi:10.1111/pbi.12162

Moon SJ, Han SY, Kim DY, Yoon IS, Shin D, Byun MO, Kwon HB, Kim BG (2015) Ectopic expression of a hot pepper bZIP-like transcription factor in potato enhances drought tolerance without decreasing tuber yield. Plant Mol Biol 89 (4-5):421-431. doi:10.1007/s11103-015-0378-y

Moreno JE, Moreno-Piovano G, Chan RL (2018) The antagonistic basic helix-loop-helix partners BEE and IBH1 contribute to control plant tolerance to abiotic stress. Plant Sci 271:143-150. doi:10.1016/j.plantsci.2018.03.024

Nakai Y, Nakahira Y, Sumida H, Takebayashi K, Nagasawa Y, Yamasaki K, Akiyama M, Ohme-Takagi M, Fujiwara S, Shiina T, Mitsuda N, Fukusaki E, Kubo Y, Sato MH (2013) Vascular plant one-zinc-finger protein 1/2 transcription factors regulate abiotic and biotic stress responses in Arabidopsis. Plant J 73 (5):761-775. doi:10.1111/tpj.12069

Nakashima K, Tran LS, Van Nguyen D, Fujita M, Maruyama K, Todaka D, Ito Y, Hayashi N, Shinozaki K, Yamaguchi-Shinozaki K (2007) Functional analysis of a NAC-type transcription factor OsNAC6 involved in abiotic and biotic stress-responsive gene expression in rice. Plant J 51 (4):617-630. doi:10.1111/j.1365-313X.2007.03168.x

Negi S, Tak H, Ganapathi TR (2018) A banana NAC transcription factor (MusaSNAC1) impart drought tolerance by modulating stomatal closure and H2O2 content. Plant Mol Biol 96 (4-5):457-471. doi:10.1007/s11103-018-0710-4

Nelson DE, Repetti PP, Adams TR, Creelman RA, Wu J, Warner DC, Anstrom DC, Bensen RJ, Castiglioni PP, Donnarummo MG, Hinchey BS, Kumimoto RW, Maszle DR, Canales RD, Krolikowski KA, Dotson SB, Gutterson N, Ratcliffe OJ, Heard JE (2007) Plant nuclear factor Y (NF-Y) B subunits confer drought tolerance and lead to improved corn yields on water-limited acres. Proc Natl Acad Sci U S A 104 (42):16450-16455. doi:10.1073/pnas.0707193104

Nguyen NC, Hoang XLT, Nguyen QT, Binh NX, Watanabe Y, Thao NP, Tran LP (2019) Ectopic Expression of Glycine max GmNAC109 Enhances Drought Tolerance and ABA Sensitivity in Arabidopsis. Biomolecules 9 (11). doi:10.3390/biom9110714

Ni Z, Hu Z, Jiang Q, Zhang H (2013) GmNFYA3, a target gene of miR169, is a positive regulator of plant tolerance to drought stress. Plant Mol Biol 82 (1-2):113-129. doi:10.1007/s11103-013-0040-5

Nie J, Wen C, Xi L, Lv S, Zhao Q, Kou Y, Ma N, Zhao L, Zhou X (2018) The AP2/ERF transcription factor CmERF053 of chrysanthemum positively regulates shoot branching, lateral root, and drought tolerance. Plant Cell Rep 37 (7):1049-1060. doi:10.1007/s00299-018-2290-9

Niu CF, Wei W, Zhou QY, Tian AG, Hao YJ, Zhang WK, Ma B, Lin Q, Zhang ZB, Zhang JS, Chen SY (2012) Wheat WRKY genes TaWRKY2 and TaWRKY19 regulate abiotic stress tolerance in transgenic Arabidopsis plants. Plant Cell Environ 35 (6):1156-1170. doi:10.1111/j.1365-3040.2012.02480.x

Oh SJ, Kim YS, Kwon CW, Park HK, Jeong JS, Kim JK (2009) Overexpression of the transcription factor AP37 in rice improves grain yield under drought conditions. Plant Physiol 150 (3):1368-1379. doi:10.1104/pp.109.137554

Orellana S, Yanez M, Espinoza A, Verdugo I, Gonzalez E, Ruiz-Lara S, Casaretto JA (2010) The transcription factor SlAREB1 confers drought, salt stress tolerance and regulates biotic and abiotic stress-related genes in tomato. Plant Cell Environ 33 (12):2191-2208. doi:10.1111/j.1365-3040.2010.02220.x

Pan Y, Hu X, Li C, Xu X, Su C, Li J, Song H, Zhang X, Pan Y (2017) SlbZIP38, a Tomato bZIP Family Gene Downregulated by Abscisic Acid, Is a Negative Regulator of Drought and Salt Stress Tolerance. Genes (Basel) 8 (12). doi:10.3390/genes8120402

Pan Y, Seymour GB, Lu C, Hu Z, Chen X, Chen G (2012) An ethylene response factor (ERF5) promoting adaptation to drought and salt tolerance in tomato. Plant Cell Rep 31 (2):349-360. doi:10.1007/s00299-011-1170-3

Pandey GK, Grant JJ, Cheong YH, Kim BG, Li L, Luan S (2005) ABR1, an APETALA2-domain transcription factor that functions as a repressor of ABA response in Arabidopsis. Plant Physiol 139 (3):1185-1193. doi:10.1104/pp.105.066324

Pandurangaiah M, Lokanadha Rao G, Sudhakarbabu O, Nareshkumar A, Kiranmai K, Lokesh U, Thapa G, Sudhakar C (2014) Overexpression of horsegram (Macrotyloma uniflorum Lam.Verdc.) NAC transcriptional factor (MuNAC4) in groundnut confers enhanced drought tolerance. Mol Biotechnol 56 (8):758-769. doi:10.1007/s12033-014-9754-0

Pang X, Xue M, Ren M, Nan D, Wu Y, Guo H (2019) Ammopiptanthus mongolicus stress-responsive NAC gene enhances the tolerance of transgenic Arabidopsis thaliana to drought and cold stresses. Genet Mol Biol 42 (3):624-634. doi:10.1590/1678-4685-GMB-2018-0101

Park SI, Kim JJ, Shin SY, Kim YS, Yoon HS (2019) ASR Enhances Environmental Stress Tolerance and Improves Grain Yield by Modulating Stomatal Closure in Rice. Front Plant Sci 10:1752. doi:10.3389/fpls.2019.01752

Park SI, Kwon HJ, Cho MH, Song JS, Kim BG, Baek J, Kim SL, Ji H, Kwon TR, Kim KH, Yoon IS (2021) The OsERF115/AP2EREBP110 Transcription Factor Is Involved in the Multiple Stress Tolerance to Heat and Drought in Rice Plants. Int J Mol Sci 22 (13). doi:10.3390/ijms22137181

Pereira SLS, Martins CPS, Sousa AO, Camillo LR, Araujo CP, Alcantara GM, Camargo DS, Cidade LC, de Almeida AF, Costa MGC (2018) Genome-wide characterization and expression analysis of citrus NUCLEAR FACTOR-Y (NF-Y) transcription factors identified a novel NF-YA gene involved in drought-stress response and tolerance. PLoS One 13 (6):e0199187. doi:10.1371/journal.pone.0199187

Perez-Diaz J, Perez-Diaz JR, Medeiros DB, Zuther E, Hong CY, Nunes-Nesi A, Hincha DK, Ruiz-Lara S, Casaretto JA (2019) Transcriptome analysis reveals potential roles of a barley ASR gene that confers stress tolerance in transgenic rice. J Plant Physiol 238:29-39. doi:10.1016/j.jplph.2019.05.005

Persak H, Pitzschke A (2014) Dominant repression by Arabidopsis transcription factor MYB44 causes oxidative damage and hypersensitivity to abiotic stress. Int J Mol Sci 15 (2):2517-2537. doi:10.3390/ijms15022517

Piao W, Sakuraba Y, Paek NC (2019) Transgenic expression of rice MYB102 (OsMYB102) delays leaf senescence and decreases abiotic stress tolerance in Arabidopsis thaliana. BMB Rep 52 (11):653-658

Qin LX, Li Y, Li DD, Xu WL, Zheng Y, Li XB (2014) Arabidopsis drought-induced protein Di19-3 participates in plant response to drought and high salinity stresses. Plant Mol Biol 86 (6):609-625. doi:10.1007/s11103-014-0251-4

Qin Y, Tian Y, Liu X (2015) A wheat salinity-induced WRKY transcription factor TaWRKY93 confers multiple abiotic stress tolerance in Arabidopsis thaliana. Biochem Biophys Res Commun 464 (2):428-433. doi:10.1016/j.bbrc.2015.06.128

Qin Y, Wang M, Tian Y, He W, Han L, Xia G (2012) Over-expression of TaMYB33 encoding a novel wheat MYB transcription factor increases salt and drought tolerance in Arabidopsis. Mol Biol Rep 39 (6):7183-7192. doi:10.1007/s11033-012-1550-y

Qiu JR, Huang Z, Xiang XY, Xu WX, Wang JT, Chen J, Song L, Xiao Y, Li X, Ma J, Cai SZ, Sun LX, Jiang CZ (2020) MfbHLH38, a Myrothamnus flabellifolia bHLH transcription factor, confers tolerance to drought and salinity stresses in Arabidopsis. BMC Plant Biol 20 (1):542. doi:10.1186/s12870-020-02732-6

Quan R, Hu S, Zhang Z, Zhang H, Zhang Z, Huang R (2010) Overexpression of an ERF transcription factor TSRF1 improves rice drought tolerance. Plant Biotechnol J 8 (4):476-488. doi:10.1111/j.1467-7652.2009.00492.x

Rahman H, Ramanathan V, Nallathambi J, Duraialagaraja S, Muthurajan R (2016) Over-expression of a NAC 67 transcription factor from finger millet (Eleusine coracana L.) confers tolerance against salinity and drought stress in rice. BMC Biotechnol 16 Suppl 1:35. doi:10.1186/s12896-016-0261-1

Raineri J, Ribichich KF, Chan RL (2015a) The sunflower transcription factor HaWRKY76 confers drought and flood tolerance to Arabidopsis thaliana plants without yield penalty. Plant Cell Rep 34 (12):2065-2080. doi:10.1007/s00299-015-1852-3

Raineri J, Wang S, Peleg Z, Blumwald E, Chan RL (2015b) The rice transcription factor OsWRKY47 is a positive regulator of the response to water deficit stress. Plant Mol Biol 88 (4-5):401-413. doi:10.1007/s11103-015-0329-7

Ramegowda V, Gill US, Sivalingam PN, Gupta A, Gupta C, Govind G, Nataraja KN, Pereira A, Udayakumar M, Mysore KS, Senthil-Kumar M (2017) GBF3 transcription factor imparts drought tolerance in Arabidopsis thaliana. Sci Rep 7 (1):9148. doi:10.1038/s41598-017-09542-1

Ramegowda V, Senthil-Kumar M, Nataraja KN, Reddy MK, Mysore KS, Udayakumar M (2012) Expression of a finger millet transcription factor, EcNAC1, in tobacco confers abiotic stress-tolerance. PLoS One 7 (7):e40397. doi:10.1371/journal.pone.0040397

Ravikumar G, Manimaran P, Voleti SR, Subrahmanyam D, Sundaram RM, Bansal KC, Viraktamath BC, Balachandran SM (2014) Stress-inducible expression of AtDREB1A transcription factor greatly improves drought stress tolerance in transgenic indica rice. Transgenic Res 23 (3):421-439. doi:10.1007/s11248-013-9776-6

Redillas MC, Jeong JS, Kim YS, Jung H, Bang SW, Choi YD, Ha SH, Reuzeau C, Kim JK (2012) The overexpression of OsNAC9 alters the root architecture of rice plants enhancing drought resistance and grain yield under field conditions. Plant Biotechnol J 10 (7):792-805. doi:10.1111/j.1467-7652.2012.00697.x

Ren X, Chen Z, Liu Y, Zhang H, Zhang M, Liu Q, Hong X, Zhu JK, Gong Z (2010) ABO3, a WRKY transcription factor, mediates plant responses to abscisic acid and drought tolerance in Arabidopsis. Plant J 63 (3):417-429. doi:10.1111/j.1365-313X.2010.04248.x

Rolly NK, Imran QM, Shahid M, Imran M, Khan M, Lee SU, Hussain A, Lee IJ, Yun BW (2020) Drought-induced AtbZIP62 transcription factor regulates drought stress response in Arabidopsis. Plant Physiol Biochem 156:384-395. doi:10.1016/j.plaphy.2020.09.013

Rong W, Qi L, Wang A, Ye X, Du L, Liang H, Xin Z, Zhang Z (2014) The ERF transcription factor TaERF3 promotes tolerance to salt and drought stresses in wheat. Plant Biotechnol J 12 (4):468-479. doi:10.1111/pbi.12153

Saad AS, Li X, Li HP, Huang T, Gao CS, Guo MW, Cheng W, Zhao GY, Liao YC (2013) A rice stress-responsive NAC gene enhances tolerance of transgenic wheat to drought and salt stresses. Plant Sci 203-204:33-40. doi:10.1016/j.plantsci.2012.12.016

Sadhukhan A, Kobayashi Y, Kobayashi Y, Tokizawa M, Yamamoto YY, Iuchi S, Koyama H, Panda SK, Sahoo L (2014) VuDREB2A, a novel DREB2-type transcription factor in the drought-tolerant legume cowpea, mediates DRE-dependent expression of stress-responsive genes and confers enhanced drought resistance in transgenic Arabidopsis. Planta 240 (3):645-664. doi:10.1007/s00425-014-2111-5

Sakamoto H, Maruyama K, Sakuma Y, Meshi T, Iwabuchi M, Shinozaki K, Yamaguchi-Shinozaki K (2004) Arabidopsis Cys2/His2-type zinc-finger proteins function as transcription repressors under drought, cold, and high-salinity stress conditions. Plant Physiol 136 (1):2734-2746. doi:10.1104/pp.104.046599

Sakuma Y, Maruyama K, Osakabe Y, Qin F, Seki M, Shinozaki K, Yamaguchi-Shinozaki K (2006) Functional analysis of an Arabidopsis transcription factor, DREB2A, involved in drought-responsive gene expression. Plant Cell 18 (5):1292-1309. doi:10.1105/tpc.105.035881

Sakuraba Y, Kim YS, Han SH, Lee BD, Paek NC (2015) The Arabidopsis Transcription Factor NAC016 Promotes Drought Stress Responses by Repressing AREB1 Transcription through a Trifurcate Feed-Forward Regulatory Loop Involving NAP. Plant Cell 27 (6):1771-1787. doi:10.1105/tpc.15.00222

Sasaki K, Ida Y, Kitajima S, Kawazu T, Hibino T, Hanba YT (2019) Overexpressing the HD-Zip class II transcription factor EcHB1 from Eucalyptus camaldulensis increased the leaf photosynthesis and drought tolerance of Eucalyptus. Sci Rep 9 (1):14121. doi:10.1038/s41598-019-50610-5

Scarpeci TE, Frea VS, Zanor MI, Valle EM (2017) Overexpression of AtERF019 delays plant growth and senescence, and improves drought tolerance in Arabidopsis. J Exp Bot 68 (3):673-685. doi:10.1093/jxb/erw429

Selvaraj MG, Jan A, Ishizaki T, Valencia M, Dedicova B, Maruyama K, Ogata T, Todaka D, Yamaguchi-Shinozaki K, Nakashima K, Ishitani M (2020) Expression of the CCCH-tandem zinc finger protein gene OsTZF5 under a stress-inducible promoter mitigates the effect of drought stress on rice grain yield under field conditions. Plant Biotechnol J 18 (8):1711-1721. doi:10.1111/pbi.13334

Sen S, Chakraborty J, Ghosh P, Basu D, Das S (2017) Chickpea WRKY70 Regulates the Expression of a Homeodomain-Leucine Zipper (HD-Zip) I Transcription Factor CaHDZ12, which Confers Abiotic Stress Tolerance in Transgenic Tobacco and Chickpea. Plant Cell Physiol 58 (11):1934-1952. doi:10.1093/pcp/pcx126

Seo PJ, Xiang F, Qiao M, Park JY, Lee YN, Kim SG, Lee YH, Park WJ, Park CM (2009) The MYB96 transcription factor mediates abscisic acid signaling during drought stress response in Arabidopsis. Plant Physiol 151 (1):275-289. doi:10.1104/pp.109.144220

Seo YJ, Park JB, Cho YJ, Jung C, Seo HS, Park SK, Nahm BH, Song JT (2010) Overexpression of the ethylene-responsive factor gene BrERF4 from Brassica rapa increases tolerance to salt and drought in Arabidopsis plants. Mol Cells 30 (3):271-277. doi:10.1007/s10059-010-0114-z

Seong SY, Shim JS, Bang SW, Kim JK (2020) Overexpression of OsC3H10, a CCCH-Zinc Finger, Improves Drought Tolerance in Rice by Regulating Stress-Related Genes. Plants (Basel) 9 (10). doi:10.3390/plants9101298

Shan H, Chen S, Jiang J, Chen F, Chen Y, Gu C, Li P, Song A, Zhu X, Gao H, Zhou G, Li T, Yang X (2012) Heterologous expression of the chrysanthemum R2R3-MYB transcription factor CmMYB2 enhances drought and salinity tolerance, increases hypersensitivity to ABA and delays flowering in Arabidopsis thaliana. Mol Biotechnol 51 (2):160-173. doi:10.1007/s12033-011-9451-1

Shang X, Yu Y, Zhu L, Liu H, Chai Q, Guo W (2020) A cotton NAC transcription factor GhirNAC2 plays positive roles in drought tolerance via regulating ABA biosynthesis. Plant Sci 296:110498. doi:10.1016/j.plantsci.2020.110498

Sharma V, Goel P, Kumar S, Singh AK (2019) An apple transcription factor, MdDREB76, confers salt and drought tolerance in transgenic tobacco by activating the expression of stress-responsive genes. Plant Cell Rep 38 (2):221-241. doi:10.1007/s00299-018-2364-8

Shen H, Liu C, Zhang Y, Meng X, Zhou X, Chu C, Wang X (2012) OsWRKY30 is activated by MAP kinases to confer drought tolerance in rice. Plant Mol Biol 80 (3):241-253. doi:10.1007/s11103-012-9941-y

Shen J, Lv B, Luo L, He J, Mao C, Xi D, Ming F (2017) The NAC-type transcription factor OsNAC2 regulates ABA-dependent genes and abiotic stress tolerance in rice. Sci Rep 7:40641. doi:10.1038/srep40641

Shi WY, Du YT, Ma J, Min DH, Jin LG, Chen J, Chen M, Zhou YB, Ma YZ, Xu ZS, Zhang XH (2018) The WRKY Transcription Factor GmWRKY12 Confers Drought and Salt Tolerance in Soybean. Int J Mol Sci 19 (12). doi:10.3390/ijms19124087

Shim JS, Oh N, Chung PJ, Kim YS, Choi YD, Kim JK (2018) Overexpression of OsNAC14 Improves Drought Tolerance in Rice. Front Plant Sci 9:310. doi:10.3389/fpls.2018.00310

Song X, Zhao Y, Wang J, Lu MZ (2021) The transcription factor KNAT2/6b mediates changes in plant architecture in response to drought via downregulating GA20ox1 in Populus alba x P. glandulosa. J Exp Bot. doi:10.1093/jxb/erab201

Song Y, Lv J, Qiu N, Bai Y, Yang N, Dong W (2019) The constitutive expression of alfalfa MsMYB2L enhances salinity and drought tolerance of Arabidopsis thaliana. Plant Physiol Biochem 141:300-305. doi:10.1016/j.plaphy.2019.06.007

Su H, Cao Y, Ku L, Yao W, Cao Y, Ren Z, Dou D, Wang H, Ren Z, Liu H, Tian L, Zheng Y, Chen C, Chen Y (2018) Dual functions of ZmNF-YA3 in photoperiod-dependent flowering and abiotic stress responses in maize. J Exp Bot 69 (21):5177-5189. doi:10.1093/jxb/ery299

Su L, Fang L, Zhu Z, Zhang L, Sun X, Wang Y, Wang Q, Li S, Xin H (2020) The transcription factor VaNAC17 from grapevine (Vitis amurensis) enhances drought tolerance by modulating jasmonic acid biosynthesis in transgenic Arabidopsis. Plant Cell Rep 39 (5):621-634. doi:10.1007/s00299-020-02519-x

Su LT, Li JW, Liu DQ, Zhai Y, Zhang HJ, Li XW, Zhang QL, Wang Y, Wang QY (2014) A novel MYB transcription factor, GmMYBJ1, from soybean confers drought and cold tolerance in Arabidopsis thaliana. Gene 538 (1):46-55. doi:10.1016/j.gene.2014.01.024

Sugano S, Kaminaka H, Rybka Z, Catala R, Salinas J, Matsui K, Ohme-Takagi M, Takatsuji H (2003) Stress-responsive zinc finger gene ZPT2-3 plays a role in drought tolerance in petunia. Plant J 36 (6):830-841. doi:10.1046/j.1365-313x.2003.01924.x

Sun J, Hu W, Zhou R, Wang L, Wang X, Wang Q, Feng Z, Li Y, Qiu D, He G, Yang G (2015) The Brachypodium distachyon BdWRKY36 gene confers tolerance to drought stress in transgenic tobacco plants. Plant Cell Rep 34 (1):23-35. doi:10.1007/s00299-014-1684-6

Sun X, Li Y, Cai H, Bai X, Ji W, Ding X, Zhu Y (2012) The Arabidopsis AtbZIP1 transcription factor is a positive regulator of plant tolerance to salt, osmotic and drought stresses. J Plant Res 125 (3):429-438. doi:10.1007/s10265-011-0448-4

Sun Y, Yu D (2015) Activated expression of AtWRKY53 negatively regulates drought tolerance by mediating stomatal movement. Plant Cell Rep 34 (8):1295-1306. doi:10.1007/s00299-015-1787-8

Sun Z, Liu R, Guo B, Huang K, Wang L, Han Y, Li H, Hou S (2019) Ectopic expression of GmZAT4, a putative C2H2-type zinc finger protein, enhances PEG and NaCl stress tolerances in Arabidopsis thaliana. 3 Biotech 9 (5):166. doi:10.1007/s13205-019-1673-0

Tak H, Negi S, Ganapathi TR (2017) Banana NAC transcription factor MusaNAC042 is positively associated with drought and salinity tolerance. Protoplasma 254 (2):803-816. doi:10.1007/s00709-016-0991-x

Tan W, Zhang D, Zhou H, Zheng T, Yin Y, Lin H (2018) Transcription factor HAT1 is a substrate of SnRK2.3 kinase and negatively regulates ABA synthesis and signaling in Arabidopsis responding to drought. PLoS Genet 14 (4):e1007336. doi:10.1371/journal.pgen.1007336

Tang L, Cai H, Ji W, Luo X, Wang Z, Wu J, Wang X, Cui L, Wang Y, Zhu Y, Bai X (2013) Overexpression of GsZFP1 enhances salt and drought tolerance in transgenic alfalfa (Medicago sativa L.). Plant Physiol Biochem 71:22-30. doi:10.1016/j.plaphy.2013.06.024

Tang N, Zhang H, Li X, Xiao J, Xiong L (2012a) Constitutive activation of transcription factor OsbZIP46 improves drought tolerance in rice. Plant Physiol 158 (4):1755-1768. doi:10.1104/pp.111.190389

Tang W, Newton RJ, Li C, Charles TM (2007) Enhanced stress tolerance in transgenic pine expressing the pepper CaPF1 gene is associated with the polyamine biosynthesis. Plant Cell Rep 26 (1):115-124. doi:10.1007/s00299-006-0228-0

Tang Y, Bao X, Zhi Y, Wu Q, Guo Y, Yin X, Zeng L, Li J, Zhang J, He W, Liu W, Wang Q, Jia C, Li Z, Liu K (2019) Overexpression of a MYB Family Gene, OsMYB6, Increases Drought and Salinity Stress Tolerance in Transgenic Rice. Front Plant Sci 10:168. doi:10.3389/fpls.2019.00168

Tang Y, Liu M, Gao S, Zhang Z, Zhao X, Zhao C, Zhang F, Chen X (2012b) Molecular characterization of novel TaNAC genes in wheat and overexpression of TaNAC2a confers drought tolerance in tobacco. Physiol Plant 144 (3):210-224. doi:10.1111/j.1399-3054.2011.01539.x

Thirumalaikumar VP, Devkar V, Mehterov N, Ali S, Ozgur R, Turkan I, Mueller-Roeber B, Balazadeh S (2018) NAC transcription factor JUNGBRUNNEN1 enhances drought tolerance in tomato. Plant Biotechnol J 16 (2):354-366. doi:10.1111/pbi.12776

Tiwari V, Chaturvedi AK, Mishra A, Jha B (2015) Introgression of the SbASR-1 gene cloned from a halophyte Salicornia brachiate enhances salinity and drought endurance in transgenic groundnut (arachis hypogaea)and acts as a transcription factor [corrected]. PLoS One 10 (7):e0131567. doi:10.1371/journal.pone.0131567

Tran LS, Nakashima K, Sakuma Y, Simpson SD, Fujita Y, Maruyama K, Fujita M, Seki M, Shinozaki K, Yamaguchi-Shinozaki K (2004) Isolation and functional analysis of Arabidopsis stress-inducible NAC transcription factors that bind to a drought-responsive cis-element in the early responsive to dehydration stress 1 promoter. Plant Cell 16 (9):2481-2498. doi:10.1105/tpc.104.022699

Tu M, Wang X, Feng T, Sun X, Wang Y, Huang L, Gao M, Wang Y, Wang X (2016) Expression of a grape (Vitis vinifera) bZIP transcription factor, VlbZIP36, in Arabidopsis thaliana confers tolerance of drought stress during seed germination and seedling establishment. Plant Sci 252:311-323. doi:10.1016/j.plantsci.2016.08.011

Tu M, Wang X, Zhu Y, Wang D, Zhang X, Cui Y, Li Y, Gao M, Li Z, Wang Y, Wang X (2018) VlbZIP30 of grapevine functions in dehydration tolerance via the abscisic acid core signaling pathway. Hortic Res 5:49. doi:10.1038/s41438-018-0054-x

van Beek CR, Guzha T, Kopana N, van der Westhuizen CS, Panda SK, van der Vyver C (2021) The SlNAC2 transcription factor from tomato confers tolerance to drought stress in transgenic tobacco plants. Physiol Mol Biol Plants 27 (5):907-921. doi:10.1007/s12298-021-00996-2

Verma RK, Kumar VVS, Yadav SK, Kumar TS, Rao MV, Chinnusamy V (2020) Overexpression of Arabidopsis ICE1 enhances yield and multiple abiotic stress tolerance in indica rice. Plant Signal Behav 15 (11):1814547. doi:10.1080/15592324.2020.1814547

Wan L, Wu Y, Huang J, Dai X, Lei Y, Yan L, Jiang H, Zhang J, Varshney RK, Liao B (2014) Identification of ERF genes in peanuts and functional analysis of AhERF008 and AhERF019 in abiotic stress response. Funct Integr Genomics 14 (3):467-477. doi:10.1007/s10142-014-0381-4

Wang B, Zhong Z, Wang X, Han X, Yu D, Wang C, Song W, Zheng X, Chen C, Zhang Y (2020a) Knockout of the OsNAC006 Transcription Factor Causes Drought and Heat Sensitivity in Rice. Int J Mol Sci 21 (7). doi:10.3390/ijms21072288

Wang C, Deng P, Chen L, Wang X, Ma H, Hu W, Yao N, Feng Y, Chai R, Yang G, He G (2013) A wheat WRKY transcription factor TaWRKY10 confers tolerance to multiple abiotic stresses in transgenic tobacco. PLoS One 8 (6):e65120. doi:10.1371/journal.pone.0065120

Wang C, Lu G, Hao Y, Guo H, Guo Y, Zhao J, Cheng H (2017a) ABP9, a maize bZIP transcription factor, enhances tolerance to salt and drought in transgenic cotton. Planta 246 (3):453-469. doi:10.1007/s00425-017-2704-x

Wang CT, Ru JN, Liu YW, Li M, Zhao D, Yang JF, Fu JD, Xu ZS (2018a) Maize WRKY Transcription Factor ZmWRKY106 Confers Drought and Heat Tolerance in Transgenic Plants. Int J Mol Sci 19 (10). doi:10.3390/ijms19103046

Wang CT, Ru JN, Liu YW, Yang JF, Li M, Xu ZS, Fu JD (2018b) The Maize WRKY Transcription Factor ZmWRKY40 Confers Drought Resistance in Transgenic Arabidopsis. Int J Mol Sci 19 (9). doi:10.3390/ijms19092580

Wang D, Chen Q, Chen W, Liu X, Xia Y, Guo Q, Jing D, Liang G (2021a) A WRKY Transcription Factor, EjWRKY17, from Eriobotrya japonica Enhances Drought Tolerance in Transgenic Arabidopsis. Int J Mol Sci 22 (11). doi:10.3390/ijms22115593

Wang F, Chen HW, Li QT, Wei W, Li W, Zhang WK, Ma B, Bi YD, Lai YC, Liu XL, Man WQ, Zhang JS, Chen SY (2015a) GmWRKY27 interacts with GmMYB174 to reduce expression of GmNAC29 for stress tolerance in soybean plants. Plant J 83 (2):224-236. doi:10.1111/tpj.12879

Wang F, Tong W, Zhu H, Kong W, Peng R, Liu Q, Yao Q (2016a) A novel Cys2/His2 zinc finger protein gene from sweetpotato, IbZFP1, is involved in salt and drought tolerance in transgenic Arabidopsis. Planta 243 (3):783-797. doi:10.1007/s00425-015-2443-9

Wang F, Zhu H, Kong W, Peng R, Liu Q, Yao Q (2016b) The Antirrhinum AmDEL gene enhances flavonoids accumulation and salt and drought tolerance in transgenic Arabidopsis. Planta 244 (1):59-73. doi:10.1007/s00425-016-2489-3

Wang G, Zhang S, Ma X, Wang Y, Kong F, Meng Q (2016c) A stress-associated NAC transcription factor (SlNAC35) from tomato plays a positive role in biotic and abiotic stresses. Physiol Plant 158 (1):45-64. doi:10.1111/ppl.12444

Wang HF, Shan HY, Shi H, Wu DD, Li TT, Li QL (2021b) Characterization of a transcription factor SlNAC7 gene from Suaeda liaotungensis and its role in stress tolerance. J Plant Res. doi:10.1007/s10265-021-01309-0

Wang J, Li Q, Mao X, Li A, Jing R (2016d) Wheat Transcription Factor TaAREB3 Participates in Drought and Freezing Tolerances in Arabidopsis. Int J Biol Sci 12 (2):257-269. doi:10.7150/ijbs.13538

Wang J, Lian W, Cao Y, Wang X, Wang G, Qi C, Liu L, Qin S, Yuan X, Li X, Ren S, Guo YD (2018c) Overexpression of BoNAC019, a NAC transcription factor from Brassica oleracea, negatively regulates the dehydration response and anthocyanin biosynthesis in Arabidopsis. Sci Rep 8 (1):13349. doi:10.1038/s41598-018-31690-1

Wang J, Wang L, Yan Y, Zhang S, Li H, Gao Z, Wang C, Guo X (2020b) GhWRKY21 regulates ABA-mediated drought tolerance by fine-tuning the expression of GhHAB in cotton. Plant Cell Rep. doi:10.1007/s00299-020-02590-4

Wang J, Zheng C, Shao X, Hu Z, Li J, Wang P, Wang A, Yu J, Shi K (2020c) Transcriptomic and genetic approaches reveal an essential role of the NAC transcription factor SlNAP1 in the growth and defense response of tomato. Hortic Res 7 (1):209. doi:10.1038/s41438-020-00442-6

Wang L, Hu W, Feng J, Yang X, Huang Q, Xiao J, Liu Y, Yang G, He G (2016e) Identification of the ASR gene family from Brachypodium distachyon and functional characterization of BdASR1 in response to drought stress. Plant Cell Rep 35 (6):1221-1234. doi:10.1007/s00299-016-1954-6

Wang L, Yu C, Chen C, He C, Zhu Y, Huang W (2014a) Identification of rice Di19 family reveals OsDi19-4 involved in drought resistance. Plant Cell Rep 33 (12):2047-2062. doi:10.1007/s00299-014-1679-3

Wang N, Cheng M, Chen Y, Liu B, Wang X, Li G, Zhou Y, Luo P, Xi Z, Yong H, Zhang D, Li M, Zhang X, Vicente FS, Hao Z, Li X (2021c) Natural variations in the non-coding region of ZmNAC080308 contributes maintaining grain yield under drought stress in maize. BMC Plant Biol 21 (1):305. doi:10.1186/s12870-021-03072-9

Wang N, Liu W, Yu L, Guo Z, Chen Z, Jiang S, Xu H, Fang H, Wang Y, Zhang Z, Chen X (2020d) HEAT SHOCK FACTOR A8a Modulates Flavonoid Synthesis and Drought Tolerance. Plant Physiol 184 (3):1273-1290. doi:10.1104/pp.20.01106

Wang N, Zhang W, Qin M, Li S, Qiao M, Liu Z, Xiang F (2017b) Drought Tolerance Conferred in Soybean (Glycine max. L) by GmMYB84, a Novel R2R3-MYB Transcription Factor. Plant Cell Physiol 58 (10):1764-1776. doi:10.1093/pcp/pcx111

Wang NN, Xu SW, Sun YL, Liu D, Zhou L, Li Y, Li XB (2019a) The cotton WRKY transcription factor (GhWRKY33) reduces transgenic Arabidopsis resistance to drought stress. Sci Rep 9 (1):724. doi:10.1038/s41598-018-37035-2

Wang Q, Guo C, Li Z, Sun J, Deng Z, Wen L, Li X, Guo Y (2021d) Potato NAC Transcription Factor StNAC053 Enhances Salt and Drought Tolerance in Transgenic Arabidopsis. Int J Mol Sci 22 (5). doi:10.3390/ijms22052568

Wang RK, Cao ZH, Hao YJ (2014b) Overexpression of a R2R3 MYB gene MdSIMYB1 increases tolerance to multiple stresses in transgenic tobacco and apples. Physiol Plant 150 (1):76-87. doi:10.1111/ppl.12069

Wang W, Liu B, Xu M, Jamil M, Wang G (2015b) ABA-induced CCCH tandem zinc finger protein OsC3H47 decreases ABA sensitivity and promotes drought tolerance in Oryza sativa. Biochem Biophys Res Commun 464 (1):33-37. doi:10.1016/j.bbrc.2015.05.087

Wang W, Qiu X, Yang Y, Kim HS, Jia X, Yu H, Kwak SS (2019b) Sweetpotato bZIP Transcription Factor IbABF4 Confers Tolerance to Multiple Abiotic Stresses. Front Plant Sci 10:630. doi:10.3389/fpls.2019.00630

Wang X, Han H, Yan J, Chen F, Wei W (2015c) A New AP2/ERF Transcription Factor from the Oil Plant Jatropha curcas Confers Salt and Drought Tolerance to Transgenic Tobacco. Appl Biochem Biotechnol 176 (2):582-597. doi:10.1007/s12010-015-1597-z

Wang X, Zeng J, Li Y, Rong X, Sun J, Sun T, Li M, Wang L, Feng Y, Chai R, Chen M, Chang J, Li K, Yang G, He G (2015d) Expression of TaWRKY44, a wheat WRKY gene, in transgenic tobacco confers multiple abiotic stress tolerances. Front Plant Sci 6:615. doi:10.3389/fpls.2015.00615

Wang Y, Cao S, Guan C, Kong X, Wang Y, Cui Y, Liu B, Zhou Y, Zhang Y (2020e) Overexpressing the NAC transcription factor LpNAC13 from Lilium pumilum in tobacco negatively regulates the drought response and positively regulates the salt response. Plant Physiol Biochem 149:96-110. doi:10.1016/j.plaphy.2020.01.036

Wang Y, Wan L, Zhang L, Zhang Z, Zhang H, Quan R, Zhou S, Huang R (2012) An ethylene response factor OsWR1 responsive to drought stress transcriptionally activates wax synthesis related genes and increases wax production in rice. Plant Mol Biol 78 (3):275-288. doi:10.1007/s11103-011-9861-2

Wang YX (2013) Characterization of a novel Medicago sativa NAC transcription factor gene involved in response to drought stress. Mol Biol Rep 40 (11):6451-6458. doi:10.1007/s11033-013-2760-7

Wang Z, Su G, Li M, Ke Q, Kim SY, Li H, Huang J, Xu B, Deng XP, Kwak SS (2016f) Overexpressing Arabidopsis ABF3 increases tolerance to multiple abiotic stresses and reduces leaf size in alfalfa. Plant Physiol Biochem 109:199-208. doi:10.1016/j.plaphy.2016.09.020

Wang Z, Tian X, Zhao Q, Liu Z, Li X, Ren Y, Tang J, Fang J, Xu Q, Bu Q (2018d) The E3 Ligase DROUGHT HYPERSENSITIVE Negatively Regulates Cuticular Wax Biosynthesis by Promoting the Degradation of Transcription Factor ROC4 in Rice. Plant Cell 30 (1):228-244. doi:10.1105/tpc.17.00823

Waseem M, Rong X, Li Z (2019) Dissecting the Role of a Basic Helix-Loop-Helix Transcription Factor, SlbHLH22, Under Salt and Drought Stresses in Transgenic Solanum lycopersicum L. Front Plant Sci 10:734. doi:10.3389/fpls.2019.00734

Wei Q, Luo Q, Wang R, Zhang F, He Y, Zhang Y, Qiu D, Li K, Chang J, Yang G, He G (2017a) A Wheat R2R3-type MYB Transcription Factor TaODORANT1 Positively Regulates Drought and Salt Stress Responses in Transgenic Tobacco Plants. Front Plant Sci 8:1374. doi:10.3389/fpls.2017.01374

Wei Q, Zhang F, Sun F, Luo Q, Wang R, Hu R, Chen M, Chang J, Yang G, He G (2017b) A wheat MYB transcriptional repressor TaMyb1D regulates phenylpropanoid metabolism and enhances tolerance to drought and oxidative stresses in transgenic tobacco plants. Plant Sci 265:112-123. doi:10.1016/j.plantsci.2017.09.020

Wei S, Xia R, Chen C, Shang X, Ge F, Wei H, Chen H, Wu Y, Xie Q (2021a) ZmbHLH124 identified in maize recombinant inbred lines contributes to drought tolerance in crops. Plant Biotechnol J. doi:10.1111/pbi.13637

Wei T, Deng K, Liu D, Gao Y, Liu Y, Yang M, Zhang L, Zheng X, Wang C, Song W, Chen C, Zhang Y (2016) Ectopic Expression of DREB Transcription Factor, AtDREB1A, Confers Tolerance to Drought in Transgenic Salvia miltiorrhiza. Plant Cell Physiol 57 (8):1593-1609. doi:10.1093/pcp/pcw084

Wei T, Deng K, Zhang Q, Gao Y, Liu Y, Yang M, Zhang L, Zheng X, Wang C, Liu Z, Chen C, Zhang Y (2017c) Modulating AtDREB1C Expression Improves Drought Tolerance in Salvia miltiorrhiza. Front Plant Sci 8:52. doi:10.3389/fpls.2017.00052

Wei W, Cui MY, Hu Y, Gao K, Xie YG, Jiang Y, Feng JY (2018) Ectopic expression of FvWRKY42, a WRKY transcription factor from the diploid woodland strawberry (Fragaria vesca), enhances resistance to powdery mildew, improves osmotic stress resistance, and increases abscisic acid sensitivity in Arabidopsis. Plant Sci 275:60-74. doi:10.1016/j.plantsci.2018.07.010

Wei W, Liang DW, Bian XH, Shen M, Xiao JH, Zhang WK, Ma B, Lin Q, Lv J, Chen X, Chen SY, Zhang JS (2019) GmWRKY54 improves drought tolerance through activating genes in abscisic acid and Ca(2+) signaling pathways in transgenic soybean. Plant J 100 (2):384-398. doi:10.1111/tpj.14449

Wei W, Zhang Y, Han L, Guan Z, Chai T (2008) A novel WRKY transcriptional factor from Thlaspi caerulescens negatively regulates the osmotic stress tolerance of transgenic tobacco. Plant Cell Rep 27 (4):795-803. doi:10.1007/s00299-007-0499-0

Wei Y, Liu W, Hu W, Yan Y, Shi H (2020) The chaperone MeHSP90 recruits MeWRKY20 and MeCatalase1 to regulate drought stress resistance in cassava. New Phytol 226 (2):476-491. doi:10.1111/nph.16346

Wei Z, Ye J, Zhou Z, Chen G, Meng F, Liu Y (2021b) Isolation and characterization of PoWRKY, an abiotic stress-related WRKY transcription factor from Polygonatum odoratum. Physiol Mol Biol Plants 27 (1):1-9. doi:10.1007/s12298-020-00924-w

Wen X, Geng F, Cheng Y, Wang J (2021) Ectopic expression of CsMYB30 from Citrus sinensis enhances salt and drought tolerance by regulating wax synthesis in Arabidopsis thaliana. Plant Physiol Biochem 166:777-788. doi:10.1016/j.plaphy.2021.06.045

Wenjing W, Chen Q, Singh PK, Huang Y, Pei D (2020) CRISPR/Cas9 edited HSFA6a and HSFA6b of Arabidopsis thaliana offers ABA and osmotic stress insensitivity by modulation of ROS homeostasis. Plant Signal Behav 15 (12):1816321. doi:10.1080/15592324.2020.1816321

Wu D, Sun Y, Wang H, Shi H, Su M, Shan H, Li T, Li Q (2018) The SlNAC8 gene of the halophyte Suaeda liaotungensis enhances drought and salt stress tolerance in transgenic Arabidopsis thaliana. Gene 662:10-20. doi:10.1016/j.gene.2018.04.012

Wu H, Fu B, Sun P, Xiao C, Liu JH (2016) A NAC Transcription Factor Represses Putrescine Biosynthesis and Affects Drought Tolerance. Plant Physiol 172 (3):1532-1547. doi:10.1104/pp.16.01096

Wu J, Folta KM, Xie Y, Jiang W, Lu J, Zhang Y (2017) Overexpression of Muscadinia rotundifolia CBF2 gene enhances biotic and abiotic stress tolerance in Arabidopsis. Protoplasma 254 (1):239-251. doi:10.1007/s00709-015-0939-6

Wu M, Liu R, Gao Y, Xiong R, Shi Y, Xiang Y (2020) PheASR2, a novel stress-responsive transcription factor from moso bamboo (Phyllostachys edulis), enhances drought tolerance in transgenic rice via increased sensitivity to abscisic acid. Plant Physiol Biochem 154:184-194. doi:10.1016/j.plaphy.2020.06.014

Wu X, Shiroto Y, Kishitani S, Ito Y, Toriyama K (2009a) Enhanced heat and drought tolerance in transgenic rice seedlings overexpressing OsWRKY11 under the control of HSP101 promoter. Plant Cell Rep 28 (1):21-30. doi:10.1007/s00299-008-0614-x

Wu Y, Deng Z, Lai J, Zhang Y, Yang C, Yin B, Zhao Q, Zhang L, Li Y, Yang C, Xie Q (2009b) Dual function of Arabidopsis ATAF1 in abiotic and biotic stress responses. Cell Res 19 (11):1279-1290. doi:10.1038/cr.2009.108

Xiang M, Ding W, Wu C, Wang W, Ye S, Cai C, Hu X, Wang N, Bai W, Tang X, Zhu C, Yu X, Xu Q, Zheng Y, Ding Z, Lin C, Zhu Q (2021a) Production of purple Ma bamboo (Dendrocalamus latiflorus Munro) with enhanced drought and cold stress tolerance by engineering anthocyanin biosynthesis. Planta 254 (3):50. doi:10.1007/s00425-021-03696-z

Xiang XY, Chen J, Xu WX, Qiu JR, Song L, Wang JT, Tang R, Chen D, Jiang CZ, Huang Z (2021b) Dehydration-Induced WRKY Transcriptional Factor MfWRKY70 of Myrothamnus flabellifolia Enhanced Drought and Salinity Tolerance in Arabidopsis. Biomolecules 11 (2). doi:10.3390/biom11020327

Xiang Y, Sun X, Bian X, Wei T, Han T, Yan J, Zhang A (2021c) The transcription factor ZmNAC49 reduces stomatal density and improves drought tolerance in maize. J Exp Bot 72 (4):1399-1410. doi:10.1093/jxb/eraa507

Xiang Y, Tang N, Du H, Ye H, Xiong L (2008) Characterization of OsbZIP23 as a key player of the basic leucine zipper transcription factor family for conferring abscisic acid sensitivity and salinity and drought tolerance in rice. Plant Physiol 148 (4):1938-1952. doi:10.1104/pp.108.128199

Xie L, Cai M, Li X, Zheng H, Xie Y, Cheng Z, Bai Y, Li J, Mu S, Gao J (2020) Overexpression of PheNAC3 from moso bamboo promotes leaf senescence and enhances abiotic stress tolerance in Arabidopsis. PeerJ 8:e8716. doi:10.7717/peerj.8716

Xie Y, Bao C, Chen P, Cao F, Liu X, Geng D, Li Z, Li X, Hou N, Zhi F, Niu C, Zhou S, Zhan X, Ma F, Guan Q (2021) Abscisic acid homeostasis is mediated by feedback regulation of MdMYB88 and MdMYB124. J Exp Bot 72 (2):592-607. doi:10.1093/jxb/eraa449

Xie Z, Nolan T, Jiang H, Tang B, Zhang M, Li Z, Yin Y (2019) The AP2/ERF Transcription Factor TINY Modulates Brassinosteroid-Regulated Plant Growth and Drought Responses in Arabidopsis. Plant Cell 31 (8):1788-1806. doi:10.1105/tpc.18.00918

Xing L, Di Z, Yang W, Liu J, Li M, Wang X, Cui C, Wang X, Wang X, Zhang R, Xiao J, Cao A (2017) Overexpression of ERF1-V from Haynaldia villosa Can Enhance the Resistance of Wheat to Powdery Mildew and Increase the Tolerance to Salt and Drought Stresses. Front Plant Sci 8:1948. doi:10.3389/fpls.2017.01948

Xiong C, Zhao S, Yu X, Sun Y, Li H, Ruan C, Li J (2020) Yellowhorn drought-induced transcription factor XsWRKY20 acts as a positive regulator in drought stress through ROS homeostasis and ABA signaling pathway. Plant Physiol Biochem 155:187-195. doi:10.1016/j.plaphy.2020.06.037

Xiong H, Li J, Liu P, Duan J, Zhao Y, Guo X, Li Y, Zhang H, Ali J, Li Z (2014) Overexpression of OsMYB48-1, a novel MYB-related transcription factor, enhances drought and salinity tolerance in rice. PLoS One 9 (3):e92913. doi:10.1371/journal.pone.0092913

Xu C, Fu X, Liu R, Guo L, Ran L, Li C, Tian Q, Jiao B, Wang B, Luo K (2017) PtoMYB170 positively regulates lignin deposition during wood formation in poplar and confers drought tolerance in transgenic Arabidopsis. Tree Physiol 37 (12):1713-1726. doi:10.1093/treephys/tpx093

Xu DB, Gao SQ, Ma YZ, Xu ZS, Zhao CP, Tang YM, Li XY, Li LC, Chen YF, Chen M (2014) ABI-like transcription factor gene TaABL1 from wheat improves multiple abiotic stress tolerances in transgenic plants. Funct Integr Genomics 14 (4):717-730. doi:10.1007/s10142-014-0394-z

Xu DQ, Huang J, Guo SQ, Yang X, Bao YM, Tang HJ, Zhang HS (2008) Overexpression of a TFIIIA-type zinc finger protein gene ZFP252 enhances drought and salt tolerance in rice (Oryza sativa L.). FEBS Lett 582 (7):1037-1043. doi:10.1016/j.febslet.2008.02.052

Xu K, Chen S, Li T, Ma X, Liang X, Ding X, Liu H, Luo L (2015) OsGRAS23, a rice GRAS transcription factor gene, is involved in drought stress response through regulating expression of stress-responsive genes. BMC Plant Biol 15:141. doi:10.1186/s12870-015-0532-3

Xu W, Tang W, Wang C, Ge L, Sun J, Qi X, He Z, Zhou Y, Chen J, Xu Z, Ma YZ, Chen M (2020a) SiMYB56 Confers Drought Stress Tolerance in Transgenic Rice by Regulating Lignin Biosynthesis and ABA Signaling Pathway. Front Plant Sci 11:785. doi:10.3389/fpls.2020.00785

Xu Y, Zhao X, Aiwaili P, Mu X, Zhao M, Zhao J, Cheng L, Ma C, Gao J, Hong B (2020b) A zinc finger protein BBX19 interacts with ABF3 to affect drought tolerance negatively in chrysanthemum. Plant J 103 (5):1783-1795. doi:10.1111/tpj.14863

Xu ZY, Kim SY, Hyeon do Y, Kim DH, Dong T, Park Y, Jin JB, Joo SH, Kim SK, Hong JC, Hwang D, Hwang I (2013) The Arabidopsis NAC transcription factor ANAC096 cooperates with bZIP-type transcription factors in dehydration and osmotic stress responses. Plant Cell 25 (11):4708-4724. doi:10.1105/tpc.113.119099

Xue GP, Way HM, Richardson T, Drenth J, Joyce PA, McIntyre CL (2011) Overexpression of TaNAC69 leads to enhanced transcript levels of stress up-regulated genes and dehydration tolerance in bread wheat. Mol Plant 4 (4):697-712. doi:10.1093/mp/ssr013

Yan H, Jia H, Chen X, Hao L, An H, Guo X (2014) The cotton WRKY transcription factor GhWRKY17 functions in drought and salt stress in transgenic Nicotiana benthamiana through ABA signaling and the modulation of reactive oxygen species production. Plant Cell Physiol 55 (12):2060-2076. doi:10.1093/pcp/pcu133

Yan Y, Jia H, Wang F, Wang C, Liu S, Guo X (2015) Overexpression of GhWRKY27a reduces tolerance to drought stress and resistance to Rhizoctonia solani infection in transgenic Nicotiana benthamiana. Front Physiol 6:265. doi:10.3389/fphys.2015.00265

Yang C, Huang Y, Lv W, Zhang Y, Bhat JA, Kong J, Xing H, Zhao J, Zhao T (2020a) GmNAC8 acts as a positive regulator in soybean drought stress. Plant Sci 293:110442. doi:10.1016/j.plantsci.2020.110442

Yang CY, Chen YC, Jauh GY, Wang CS (2005) A Lily ASR protein involves abscisic acid signaling and confers drought and salt resistance in Arabidopsis. Plant Physiol 139 (2):836-846. doi:10.1104/pp.105.065458

Yang S, Xu K, Chen S, Li T, Xia H, Chen L, Liu H, Luo L (2019a) A stress-responsive bZIP transcription factor OsbZIP62 improves drought and oxidative tolerance in rice. BMC Plant Biol 19 (1):260. doi:10.1186/s12870-019-1872-1

Yang T, Yao S, Hao L, Zhao Y, Lu W, Xiao K (2016a) Wheat bHLH-type transcription factor gene TabHLH1 is crucial in mediating osmotic stresses tolerance through modulating largely the ABA-associated pathway. Plant Cell Rep 35 (11):2309-2323. doi:10.1007/s00299-016-2036-5

Yang W, Liu XD, Chi XJ, Wu CA, Li YZ, Song LL, Liu XM, Wang YF, Wang FW, Zhang C, Liu Y, Zong JM, Li HY (2011) Dwarf apple MbDREB1 enhances plant tolerance to low temperature, drought, and salt stress via both ABA-dependent and ABA-independent pathways. Planta 233 (2):219-229. doi:10.1007/s00425-010-1279-6

Yang X, He K, Chi X, Chai G, Wang Y, Jia C, Zhang H, Zhou G, Hu R (2018a) Miscanthus NAC transcription factor MlNAC12 positively mediates abiotic stress tolerance in transgenic Arabidopsis. Plant Sci 277:229-241. doi:10.1016/j.plantsci.2018.09.013

Yang X, Kim MY, Ha J, Lee SH (2019b) Overexpression of the Soybean NAC Gene GmNAC109 Increases Lateral Root Formation and Abiotic Stress Tolerance in Transgenic Arabidopsis Plants. Front Plant Sci 10:1036. doi:10.3389/fpls.2019.01036

Yang X, Wang X, Ji L, Yi Z, Fu C, Ran J, Hu R, Zhou G (2015) Overexpression of a Miscanthus lutarioriparius NAC gene MlNAC5 confers enhanced drought and cold tolerance in Arabidopsis. Plant Cell Rep 34 (6):943-958. doi:10.1007/s00299-015-1756-2

Yang Y, Dong C, Li X, Du J, Qian M, Sun X, Yang Y (2016b) A novel Ap2/ERF transcription factor from Stipa purpurea leads to enhanced drought tolerance in Arabidopsis thaliana. Plant Cell Rep 35 (11):2227-2239. doi:10.1007/s00299-016-2030-y

Yang Y, Luang S, Harris J, Riboni M, Li Y, Bazanova N, Hrmova M, Haefele S, Kovalchuk N, Lopato S (2018b) Overexpression of the class I homeodomain transcription factor TaHDZipI-5 increases drought and frost tolerance in transgenic wheat. Plant Biotechnol J 16 (6):1227-1240. doi:10.1111/pbi.12865

Yang Y, Yu TF, Ma J, Chen J, Zhou YB, Chen M, Ma YZ, Wei WL, Xu ZS (2020b) The Soybean bZIP Transcription Factor Gene GmbZIP2 Confers Drought and Salt Resistances in Transgenic Plants. Int J Mol Sci 21 (2). doi:10.3390/ijms21020670

Yang YY, Zheng PF, Ren YR, Yao YX, You CX, Wang XF, Hao YJ (2021) Apple MdSAT1 encodes a bHLHm1 transcription factor involved in salinity and drought responses. Planta 253 (2):46. doi:10.1007/s00425-020-03528-6

Yang Z, Chi X, Guo F, Jin X, Luo H, Hawar A, Chen Y, Feng K, Wang B, Qi J, Yang Y, Sun B (2020c) SbWRKY30 enhances the drought tolerance of plants and regulates a drought stress-responsive gene, SbRD19, in sorghum. J Plant Physiol 246-247:153142. doi:10.1016/j.jplph.2020.153142

Yao L, Jiang Y, Lu X, Wang B, Zhou P, Wu T (2016) A R2R3-MYB transcription factor from Lablab purpureus induced by drought increases tolerance to abiotic stress in Arabidopsis. Mol Biol Rep 43 (10):1089-1100. doi:10.1007/s11033-016-4042-7

Yao PF, Li CL, Zhao XR, Li MF, Zhao HX, Guo JY, Cai Y, Chen H, Wu Q (2017) Overexpression of a Tartary Buckwheat Gene, FtbHLH3, Enhances Drought/Oxidative Stress Tolerance in Transgenic Arabidopsis. Front Plant Sci 8:625. doi:10.3389/fpls.2017.00625

Ye Y, Lin R, Su H, Chen H, Luo M, Yang L, Zhang M (2019) The functional identification of glycine-rich TtASR from Tetragonia tetragonoides (Pall.) Kuntze involving in plant abiotic stress tolerance. Plant Physiol Biochem 143:212-223. doi:10.1016/j.plaphy.2019.09.013

Yin M, Wang Y, Zhang L, Li J, Quan W, Yang L, Wang Q, Chan Z (2017) The Arabidopsis Cys2/His2 zinc finger transcription factor ZAT18 is a positive regulator of plant tolerance to drought stress. J Exp Bot 68 (11):2991-3005. doi:10.1093/jxb/erx157

Yin Y, Jiang X, Ren M, Xue M, Nan D, Wang Z, Xing Y, Wang M (2018) AmDREB2C, from Ammopiptanthus mongolicus, enhances abiotic stress tolerance and regulates fatty acid composition in transgenic Arabidopsis. Plant Physiol Biochem 130:517-528. doi:10.1016/j.plaphy.2018.08.002

Ying S, Zhang DF, Fu J, Shi YS, Song YC, Wang TY, Li Y (2012) Cloning and characterization of a maize bZIP transcription factor, ZmbZIP72, confers drought and salt tolerance in transgenic Arabidopsis. Planta 235 (2):253-266. doi:10.1007/s00425-011-1496-7

Yong Y, Zhang Y, Lyu Y (2019a) A MYB-Related Transcription Factor from Lilium lancifolium L. (LlMYB3) Is Involved in Anthocyanin Biosynthesis Pathway and Enhances Multiple Abiotic Stress Tolerance in Arabidopsis thaliana. Int J Mol Sci 20 (13). doi:10.3390/ijms20133195

Yong Y, Zhang Y, Lyu Y (2019b) A Stress-Responsive NAC Transcription Factor from Tiger Lily (LlNAC2) Interacts with LlDREB1 and LlZHFD4 and Enhances Various Abiotic Stress Tolerance in Arabidopsis. Int J Mol Sci 20 (13). doi:10.3390/ijms20133225

Yoo CY, Pence HE, Jin JB, Miura K, Gosney MJ, Hasegawa PM, Mickelbart MV (2010) The Arabidopsis GTL1 transcription factor regulates water use efficiency and drought tolerance by modulating stomatal density via transrepression of SDD1. Plant Cell 22 (12):4128-4141. doi:10.1105/tpc.110.078691

Yoon JS, Kim JY, Kim DY, Seo YW (2021) A novel wheat ASR gene, TaASR2D, enhances drought tolerance in Brachypodium distachyon. Plant Physiol Biochem 159:400-414. doi:10.1016/j.plaphy.2020.11.014

Yoon JS, Kim JY, Lee MB, Seo YW (2019) Over-expression of the Brachypodium ASR gene, BdASR4, enhances drought tolerance in Brachypodium distachyon. Plant Cell Rep 38 (9):1109-1125. doi:10.1007/s00299-019-02429-7

Yoshida T, Fujita Y, Maruyama K, Mogami J, Todaka D, Shinozaki K, Yamaguchi-Shinozaki K (2015) Four Arabidopsis AREB/ABF transcription factors function predominantly in gene expression downstream of SnRK2 kinases in abscisic acid signalling in response to osmotic stress. Plant Cell Environ 38 (1):35-49. doi:10.1111/pce.12351

Yoshida T, Fujita Y, Sayama H, Kidokoro S, Maruyama K, Mizoi J, Shinozaki K, Yamaguchi-Shinozaki K (2010) AREB1, AREB2, and ABF3 are master transcription factors that cooperatively regulate ABRE-dependent ABA signaling involved in drought stress tolerance and require ABA for full activation. Plant J 61 (4):672-685. doi:10.1111/j.1365-313X.2009.04092.x

Yu H, Chen X, Hong YY, Wang Y, Xu P, Ke SD, Liu HY, Zhu JK, Oliver DJ, Xiang CB (2008) Activated expression of an Arabidopsis HD-START protein confers drought tolerance with improved root system and reduced stomatal density. Plant Cell 20 (4):1134-1151. doi:10.1105/tpc.108.058263

Yu L, Chen X, Wang Z, Wang S, Wang Y, Zhu Q, Li S, Xiang C (2013) Arabidopsis enhanced drought tolerance1/HOMEODOMAIN GLABROUS11 confers drought tolerance in transgenic rice without yield penalty. Plant Physiol 162 (3):1378-1391. doi:10.1104/pp.113.217596

Yu LH, Wu SJ, Peng YS, Liu RN, Chen X, Zhao P, Xu P, Zhu JB, Jiao GL, Pei Y, Xiang CB (2016a) Arabidopsis EDT1/HDG11 improves drought and salt tolerance in cotton and poplar and increases cotton yield in the field. Plant Biotechnol J 14 (1):72-84. doi:10.1111/pbi.12358

Yu M, Liu J, Du B, Zhang M, Wang A, Zhang L (2021) NAC Transcription Factor PwNAC11 Activates ERD1 by Interaction with ABF3 and DREB2A to Enhance Drought Tolerance in Transgenic Arabidopsis. Int J Mol Sci 22 (13). doi:10.3390/ijms22136952

Yu X, Liu Y, Wang S, Tao Y, Wang Z, Shu Y, Peng H, Mijiti A, Wang Z, Zhang H, Ma H (2016b) CarNAC4, a NAC-type chickpea transcription factor conferring enhanced drought and salt stress tolerances in Arabidopsis. Plant Cell Rep 35 (3):613-627. doi:10.1007/s00299-015-1907-5

Yu Y, Bi C, Wang Q, Ni Z (2019) Overexpression of TaSIM provides increased drought stress tolerance in transgenic Arabidopsis. Biochem Biophys Res Commun 512 (1):66-71. doi:10.1016/j.bbrc.2019.03.007

Yu YT, Wu Z, Lu K, Bi C, Liang S, Wang XF, Zhang DP (2016c) Overexpression of the MYB37 transcription factor enhances abscisic acid sensitivity, and improves both drought tolerance and seed productivity in Arabidopsis thaliana. Plant Mol Biol 90 (3):267-279. doi:10.1007/s11103-015-0411-1

Yuan X, Huang P, Wang R, Li H, Lv X, Duan M, Tang H, Zhang H, Huang J (2018) A Zinc Finger Transcriptional Repressor Confers Pleiotropic Effects on Rice Growth and Drought Tolerance by Down-Regulating Stress-Responsive Genes. Plant Cell Physiol 59 (10):2129-2142. doi:10.1093/pcp/pcy133

Yuan X, Wang H, Cai J, Bi Y, Li D, Song F (2019) Rice NAC transcription factor ONAC066 functions as a positive regulator of drought and oxidative stress response. BMC Plant Biol 19 (1):278. doi:10.1186/s12870-019-1883-y

Yue X, Zhang G, Zhao Z, Yue J, Pu X, Sui M, Zhan Y, Shi Y, Wang Z, Meng G, Zhao Z, An L (2019) A Cryophyte Transcription Factor, CbABF1, Confers Freezing, and Drought Tolerance in Tobacco. Front Plant Sci 10:699. doi:10.3389/fpls.2019.00699

Zhai Y, Zhang L, Xia C, Fu S, Zhao G, Jia J, Kong X (2016) The wheat transcription factor, TabHLH39, improves tolerance to multiple abiotic stressors in transgenic plants. Biochem Biophys Res Commun 473 (4):1321-1327. doi:10.1016/j.bbrc.2016.04.071

Zhang B, Su L, Hu B, Li L (2018a) Expression of AhDREB1, an AP2/ERF Transcription Factor Gene from Peanut, Is Affected by Histone Acetylation and Increases Abscisic Acid Sensitivity and Tolerance to Osmotic Stress in Arabidopsis. Int J Mol Sci 19 (5). doi:10.3390/ijms19051441

Zhang CY, Liu HC, Zhang XS, Guo QX, Bian SM, Wang JY, Zhai LL (2020a) VcMYB4a, an R2R3-MYB transcription factor from Vaccinium corymbosum, negatively regulates salt, drought, and temperature stress. Gene 757:144935. doi:10.1016/j.gene.2020.144935

Zhang D, Tong J, Xu Z, Wei P, Xu L, Wan Q, Huang Y, He X, Yang J, Shao H, Ma H (2016) Soybean C2H2-Type Zinc Finger Protein GmZFP3 with Conserved QALGGH Motif Negatively Regulates Drought Responses in Transgenic Arabidopsis. Front Plant Sci 7:325. doi:10.3389/fpls.2016.00325

Zhang G, Chen M, Li L, Xu Z, Chen X, Guo J, Ma Y (2009) Overexpression of the soybean GmERF3 gene, an AP2/ERF type transcription factor for increased tolerances to salt, drought, and diseases in transgenic tobacco. J Exp Bot 60 (13):3781-3796. doi:10.1093/jxb/erp214

Zhang G, Huang S, Zhang C, Li D, Wu Y, Deng J, Shan S, Qi J (2021) Overexpression of CcNAC1 gene promotes early flowering and enhances drought tolerance of jute (Corchorus capsularis L.). Protoplasma 258 (2):337-345. doi:10.1007/s00709-020-01569-y

Zhang H, Cui X, Guo Y, Luo C, Zhang L (2018b) Picea wilsonii transcription factor NAC2 enhanced plant tolerance to abiotic stress and participated in RFCP1-regulated flowering time. Plant Mol Biol 98 (6):471-493. doi:10.1007/s11103-018-0792-z

Zhang H, Gao X, Zhi Y, Li X, Zhang Q, Niu J, Wang J, Zhai H, Zhao N, Li J, Liu Q, He S (2019a) A non-tandem CCCH-type zinc-finger protein, IbC3H18, functions as a nuclear transcriptional activator and enhances abiotic stress tolerance in sweet potato. New Phytol 223 (4):1918-1936. doi:10.1111/nph.15925

Zhang JY, Broeckling CD, Blancaflor EB, Sledge MK, Sumner LW, Wang ZY (2005) Overexpression of WXP1, a putative Medicago truncatula AP2 domain-containing transcription factor gene, increases cuticular wax accumulation and enhances drought tolerance in transgenic alfalfa (Medicago sativa). Plant J 42 (5):689-707. doi:10.1111/j.1365-313X.2005.02405.x

Zhang JY, Broeckling CD, Sumner LW, Wang ZY (2007) Heterologous expression of two Medicago truncatula putative ERF transcription factor genes, WXP1 and WXP2, in Arabidopsis led to increased leaf wax accumulation and improved drought tolerance, but differential response in freezing tolerance. Plant Mol Biol 64 (3):265-278. doi:10.1007/s11103-007-9150-2

Zhang L, Cheng J, Sun X, Zhao T, Li M, Wang Q, Li S, Xin H (2018c) Overexpression of VaWRKY14 increases drought tolerance in Arabidopsis by modulating the expression of stress-related genes. Plant Cell Rep 37 (8):1159-1172. doi:10.1007/s00299-018-2302-9

Zhang L, Hu W, Wang Y, Feng R, Zhang Y, Liu J, Jia C, Miao H, Zhang J, Xu B, Jin Z (2015a) The MaASR gene as a crucial component in multiple drought stress response pathways in Arabidopsis. Funct Integr Genomics 15 (2):247-260. doi:10.1007/s10142-014-0415-y

Zhang L, Zhang L, Xia C, Zhao G, Jia J, Kong X (2015b) The Novel Wheat Transcription Factor TaNAC47 Enhances Multiple Abiotic Stress Tolerances in Transgenic Plants. Front Plant Sci 6:1174. doi:10.3389/fpls.2015.01174

Zhang L, Zhang L, Xia C, Zhao G, Liu J, Jia J, Kong X (2015c) A novel wheat bZIP transcription factor, TabZIP60, confers multiple abiotic stress tolerances in transgenic Arabidopsis. Physiol Plant 153 (4):538-554. doi:10.1111/ppl.12261

Zhang L, Zhao G, Xia C, Jia J, Liu X, Kong X (2012a) A wheat R2R3-MYB gene, TaMYB30-B, improves drought stress tolerance in transgenic Arabidopsis. J Exp Bot 63 (16):5873-5885. doi:10.1093/jxb/ers237

Zhang M, Liu Y, Cai H, Guo M, Chai M, She Z, Ye L, Cheng Y, Wang B, Qin Y (2020b) The bZIP Transcription Factor GmbZIP15 Negatively Regulates Salt- and Drought-Stress Responses in Soybean. Int J Mol Sci 21 (20). doi:10.3390/ijms21207778

Zhang S, Haider I, Kohlen W, Jiang L, Bouwmeester H, Meijer AH, Schluepmann H, Liu CM, Ouwerkerk PB (2012b) Function of the HD-Zip I gene Oshox22 in ABA-mediated drought and salt tolerances in rice. Plant Mol Biol 80 (6):571-585. doi:10.1007/s11103-012-9967-1

Zhang T, Zhang D, Liu Y, Luo C, Zhou Y, Zhang L (2015d) Overexpression of a NF-YB3 transcription factor from Picea wilsonii confers tolerance to salinity and drought stress in transformed Arabidopsis thaliana. Plant Physiol Biochem 94:153-164. doi:10.1016/j.plaphy.2015.05.001

Zhang X, Wang L, Meng H, Wen H, Fan Y, Zhao J (2011) Maize ABP9 enhances tolerance to multiple stresses in transgenic Arabidopsis by modulating ABA signaling and cellular levels of reactive oxygen species. Plant Mol Biol 75 (4-5):365-378. doi:10.1007/s11103-011-9732-x

Zhang Y, Li J, Chen S, Ma X, Wei H, Chen C, Gao N, Zou Y, Kong D, Li T, Liu Z, Yu S, Luo L (2020c) An APETALA2/ethylene responsive factor, OsEBP89 knockout enhances adaptation to direct-seeding on wet land and tolerance to drought stress in rice. Mol Genet Genomics 295 (4):941-956. doi:10.1007/s00438-020-01669-7

Zhang YL, Zhang CL, Wang GL, Wang YX, Qi CH, You CX, Li YY, Hao YJ (2019b) Apple AP2/EREBP transcription factor MdSHINE2 confers drought resistance by regulating wax biosynthesis. Planta 249 (5):1627-1643. doi:10.1007/s00425-019-03115-4

Zhang Z, Liu X, Wang X, Zhou M, Zhou X, Ye X, Wei X (2012c) An R2R3 MYB transcription factor in wheat, TaPIMP1, mediates host resistance to Bipolaris sorokiniana and drought stresses through regulation of defense- and stress-related genes. New Phytol 196 (4):1155-1170. doi:10.1111/j.1469-8137.2012.04353.x

Zhao BY, Hu YF, Li JJ, Yao X, Liu KD (2016a) BnaABF2, a bZIP transcription factor from rapeseed (Brassica napus L.), enhances drought and salt tolerance in transgenic Arabidopsis. Bot Stud 57 (1):12. doi:10.1186/s40529-016-0127-9

Zhao Q, Fan Z, Qiu L, Che Q, Wang T, Li Y, Wang Y (2020a) MdbHLH130, an Apple bHLH Transcription Factor, Confers Water Stress Resistance by Regulating Stomatal Closure and ROS Homeostasis in Transgenic Tobacco. Front Plant Sci 11:543696. doi:10.3389/fpls.2020.543696

Zhao Q, Hu RS, Liu D, Liu X, Wang J, Xiang XH, Li YY (2020b) The AP2 transcription factor NtERF172 confers drought resistance by modifying NtCAT. Plant Biotechnol J 18 (12):2444-2455. doi:10.1111/pbi.13419

Zhao X, Yang X, Pei S, He G, Wang X, Tang Q, Jia C, Lu Y, Hu R, Zhou G (2016b) The Miscanthus NAC transcription factor MlNAC9 enhances abiotic stress tolerance in transgenic Arabidopsis. Gene 586 (1):158-169. doi:10.1016/j.gene.2016.04.028

Zhao Y, Cheng X, Liu X, Wu H, Bi H, Xu H (2018) The Wheat MYB Transcription Factor TaMYB(31) Is Involved in Drought Stress Responses in Arabidopsis. Front Plant Sci 9:1426. doi:10.3389/fpls.2018.01426

Zhao Y, Ma Q, Jin X, Peng X, Liu J, Deng L, Yan H, Sheng L, Jiang H, Cheng B (2014) A novel maize homeodomain-leucine zipper (HD-Zip) I gene, Zmhdz10, positively regulates drought and salt tolerance in both rice and Arabidopsis. Plant Cell Physiol 55 (6):1142-1156. doi:10.1093/pcp/pcu054

Zheng L, Liu G, Meng X, Liu Y, Ji X, Li Y, Nie X, Wang Y (2013) A WRKY gene from Tamarix hispida, ThWRKY4, mediates abiotic stress responses by modulating reactive oxygen species and expression of stress-responsive genes. Plant Mol Biol 82 (4-5):303-320. doi:10.1007/s11103-013-0063-y

Zheng X, Chen B, Lu G, Han B (2009) Overexpression of a NAC transcription factor enhances rice drought and salt tolerance. Biochem Biophys Res Commun 379 (4):985-989. doi:10.1016/j.bbrc.2008.12.163

Zhong L, Chen D, Min D, Li W, Xu Z, Zhou Y, Li L, Chen M, Ma Y (2015) AtTGA4, a bZIP transcription factor, confers drought resistance by enhancing nitrate transport and assimilation in Arabidopsis thaliana. Biochem Biophys Res Commun 457 (3):433-439. doi:10.1016/j.bbrc.2015.01.009

Zhou ML, Ma JT, Zhao YM, Wei YH, Tang YX, Wu YM (2012) Improvement of drought and salt tolerance in Arabidopsis and Lotus corniculatus by overexpression of a novel DREB transcription factor from Populus euphratica. Gene 506 (1):10-17. doi:10.1016/j.gene.2012.06.089

Zhou QY, Tian AG, Zou HF, Xie ZM, Lei G, Huang J, Wang CM, Wang HW, Zhang JS, Chen SY (2008) Soybean WRKY-type transcription factor genes, GmWRKY13, GmWRKY21, and GmWRKY54, confer differential tolerance to abiotic stresses in transgenic Arabidopsis plants. Plant Biotechnol J 6 (5):486-503. doi:10.1111/j.1467-7652.2008.00336.x

Zhou Y, Zhang Y, Wang X, Han X, An Y, Lin S, Shen C, Wen J, Liu C, Yin W, Xia X (2020) Root-specific NF-Y family transcription factor, PdNF-YB21, positively regulates root growth and drought resistance by abscisic acid-mediated indoylacetic acid transport in Populus. New Phytol 227 (2):407-426. doi:10.1111/nph.16524

Zhou Y, Zhu H, He S, Zhai H, Zhao N, Xing S, Wei Z, Liu Q (2019) A Novel Sweetpotato Transcription Factor Gene IbMYB116 Enhances Drought Tolerance in Transgenic Arabidopsis. Front Plant Sci 10:1025. doi:10.3389/fpls.2019.01025

Zhu D, Wu Z, Cao G, Li J, Wei J, Tsuge T, Gu H, Aoyama T, Qu LJ (2014a) TRANSLUCENT GREEN, an ERF family transcription factor, controls water balance in Arabidopsis by activating the expression of aquaporin genes. Mol Plant 7 (4):601-615. doi:10.1093/mp/sst152

Zhu H, Zhou Y, Zhai H, He S, Zhao N, Liu Q (2020a) A Novel Sweetpotato WRKY Transcription Factor, IbWRKY2, Positively Regulates Drought and Salt Tolerance in Transgenic Arabidopsis. Biomolecules 10 (4). doi:10.3390/biom10040506

Zhu M, Chen G, Zhang J, Zhang Y, Xie Q, Zhao Z, Pan Y, Hu Z (2014b) The abiotic stress-responsive NAC-type transcription factor SlNAC4 regulates salt and drought tolerance and stress-related genes in tomato (Solanum lycopersicum). Plant Cell Rep 33 (11):1851-1863. doi:10.1007/s00299-014-1662-z

Zhu M, Meng X, Cai J, Li G, Dong T, Li Z (2018) Basic leucine zipper transcription factor SlbZIP1 mediates salt and drought stress tolerance in tomato. BMC Plant Biol 18 (1):83. doi:10.1186/s12870-018-1299-0

Zhu MD, Zhang M, Gao DJ, Zhou K, Tang SJ, Zhou B, Lv YM (2020b) Rice OsHSFA3 Gene Improves Drought Tolerance by Modulating Polyamine Biosynthesis Depending on Abscisic Acid and ROS Levels. Int J Mol Sci 21 (5). doi:10.3390/ijms21051857

Zhu Y, Wang Z, Jing Y, Wang L, Liu X, Liu Y, Deng X (2009) Ectopic over-expression of BhHsf1, a heat shock factor from the resurrection plant Boea hygrometrica, leads to increased thermotolerance and retarded growth in transgenic Arabidopsis and tobacco. Plant Mol Biol 71 (4-5):451-467. doi:10.1007/s11103-009-9538-2

Zhu Y, Yan J, Liu W, Liu L, Sheng Y, Sun Y, Li Y, Scheller HV, Jiang M, Hou X, Ni L, Zhang A (2016a) Phosphorylation of a NAC Transcription Factor by a Calcium/Calmodulin-Dependent Protein Kinase Regulates Abscisic Acid-Induced Antioxidant Defense in Maize. Plant Physiol 171 (3):1651-1664. doi:10.1104/pp.16.00168

Zhu Z, Sun B, Xu X, Chen H, Zou L, Chen G, Cao B, Chen C, Lei J (2016b) Overexpression of AtEDT1/HDG11 in Chinese Kale (Brassica oleracea var. alboglabra) Enhances Drought and Osmotic Stress Tolerance. Front Plant Sci 7:1285. doi:10.3389/fpls.2016.01285

Zhuang Y, Wang C, Zhang Y, Chen S, Wang D, Liu Q, Zhou G, Chai G (2019) Overexpression of PdC3H17 Confers Tolerance to Drought Stress Depending on Its CCCH Domain in Populus. Front Plant Sci 10:1748. doi:10.3389/fpls.2019.01748

Zuo ZF, Kang HG, Hong QC, Park MY, Sun HJ, Kim J, Song PS, Lee HY (2020) A novel basic helix-loop-helix transcription factor, ZjICE2 from Zoysia japonica confers abiotic stress tolerance to transgenic plants via activating the DREB/CBF regulon and enhancing ROS scavenging. Plant Mol Biol 102 (4-5):447-462. doi:10.1007/s11103-019-00957-0
